# Disentangling Electronic Interactions in f^7^ Systems: A Comparative Study on Gd**^III^**, Tb**^IV^**, Cm**^III^**, and Bk**^IV^**

**DOI:** 10.1021/acs.inorgchem.6c02332

**Published:** 2026-09-14

**Authors:** Chad M. Studvick, Ivan A. Popov, Cristian Celis-Barros

**Affiliations:** † Department of Chemistry, University of Akron, Akron, Ohio 44325-3601, United States; ‡ Department of Chemistry, 6760 Washington State University, Pullman, Washington 99164, United States; § Radioisotope Science and Technology Division, 6146 Oak Ridge National Laboratory, Oak Ridge, Tennessee 37830, United States

## Abstract

In this work, we demonstrate through computational spectroscopic analysis of four experimentally accessible f^7^ ions within complexes (Gd^III^, Tb^IV^, Cm^III^, and Bk^IV^) the extent to which electron–electron repulsion, spin–orbit coupling, and the identity of the ligand field influence the energy and intermediate coupling of an f^7^ ion’s low-lying excited states. As expected, the energy of excited states is primarily governed by electron–electron repulsion in the lanthanides (Gd^III^ and Tb^IV^), whereas spin–orbit coupling and ligand field effects play an increasingly important role in the actinides (Cm^III^ and Bk^IV^). Additionally, the stabilization caused by a ligand field is significantly greater for ions in higher oxidation states, as evidenced by the decreased energy of the emissive state and the nephelauxetic reduction. Finally, mixing Russell–Saunders terms in an intermediate coupling scheme is shown to be more significant in actinides and at higher oxidation states, with Tb^IV^ exhibiting signs of behaving spectroscopically like an actinide. These results reveal a clear correlation between composition, excited-state energy, and covalency, which supports a direct correlation between f-orbital covalency and the f–f multiplet structure. Furthermore, these findings contribute to spectroscopic studies on high-valent f-element complexes.

## Graphical abstract

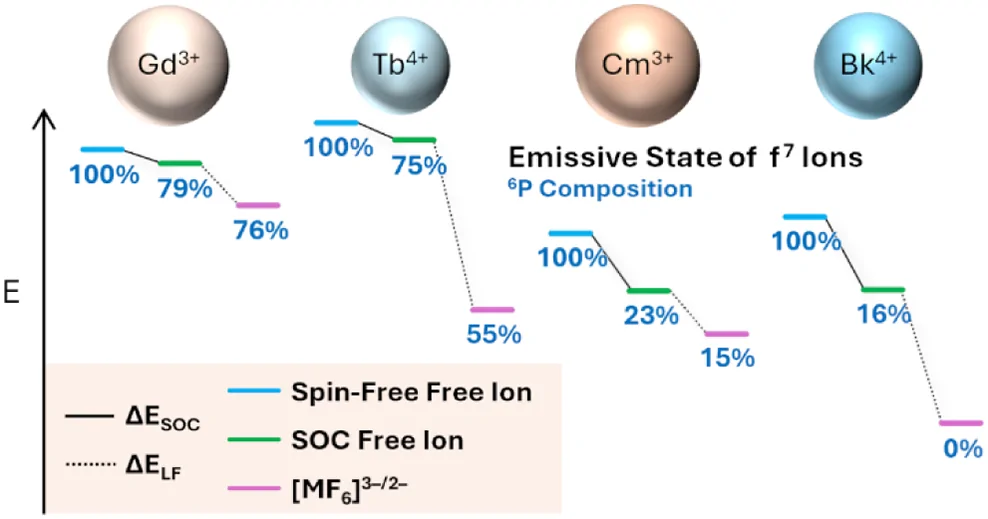

## Introduction

Identifying and separating actinides and lanthanides remain key areas of research that are integral to several fields. Identification and separation are necessary for partitioning and transmuting minor actinides in spent nuclear fuel, which aid in recycling and nonproliferation, as well as synthesizing pure samples of lanthanide and actinide isotopes that can then be used for many different purposes. Such applications include rich neutron sources primarily for the petroleum and mining industries,^1–5^ power sources for spacecraft,^6–8^ radiotherapy treatments,^9,10^ and nuclear batteries.^11,12^ With the vast array of technologies that incorporate these isotopes, active research is ongoing to identify features that can be leveraged to simplify their production processes.^13^

Spectroscopy offers an analytical approach to identify several different species in small quantities in a mixture. The spectroscopic signatures of lanthanides and actinides have been studied for several decades,^14–25^ with a specific focus on f–f transitions,^23^ for a variety of technological and medical applications,^26–28^ as well as speciation of actinide systems.^29,30^ For the mid- to late-actinides, particular interest has centered on Am and Cm because they make up the harmful minor actinides in spent nuclear fuel.^31–42^ To a lesser extent, heavier actinides such as Cf have also been characterized.^35,43–45^

However, spectroscopy involving formally Laporte forbidden f–f transitions of lanthanides and actinides is experimentally more difficult than allowed transitions because of their intricate electronic structures arising from the electrons that occupy the f-shell. With this, a large number of excited-state transitions can occur. An interesting and special case is the f^7^ electron configuration. It offers a simplified yet powerful electronic structure that allows researchers to infer fundamental chemical properties of metal–ligand interactions. Because of the larger energy gap between the ground state (*J* = 7/2) and the first excited multiplet (*J* = 7/2), f^7^ systems can be investigated not only using optical absorption but also emission spectroscopy. Regarding electronic structure, complexes with an f^7^ ion have potential for exhibiting intense photoluminescence, such as the characteristic orange emission observed in several Cm^III^ complexes.^22^

By far, Gd^III^ and Cm^III^ are the most common f^7^-configured ions in the lanthanide and actinide series, respectively, because of the proclivity of mid- to late lanthanides and actinides to favor the +3 oxidation state. Two other ions with an f^7^ configuration, Tb^IV^ and Bk^IV^, also have received interest in recent years because of developed ligands that can stabilize these higher oxidation states.^46–62^ All these ions have had spectroscopic studies performed in the past: Gd^III^ has been widely studied because it is nonradioactive and readily available in larger quantities than its radioactive isoelectronic actinide counterpart, Cm^III^. However, the luminescence of Cm^III^ has also been proven as a useful tool for speciation; it aids in identification in natural environments and separation from Am^III^ in spent nuclear fuel.^63^ In nonaqueous solutions, Cm^III^ has been studied extensively using time-resolved laser-induced fluorescence spectroscopy^36,64–69^ to determine stability constants and thermodynamic data of complexation reactions. Cm^III^ has also proven useful for elucidating the effects pressure can induce on spectroscopic signatures.^70,71^ Schmidt et al. reviewed several other recent developments in *Cm* ^III^ spectroscopy for species determination.^72^

Conversely, studies on Tb^IV^ are limited because of its inherent instability, but do exist.^51–56^ Likewise, because of its relative scarcity, only a few spectroscopic studies have been performed on Bk^IV^, such as being doped into a CeF_4_ matrix to simulate a free ion,^57^ complexed with iodates,^58^ and as the center of a ring sandwich,^59^ and recently with hydroxypyridinone derivatives,^73^ among others.^60–62^ For Tb and (to a lesser extent) Bk, the reduced stability of the tetravalent oxidation state is expected to result in broader ligand-to-metal charge transfer bands shifted into the visible region.^46–51^ These bands tend to dominate the spectrum and obscure underlying f–f transitions, which limit direct insight into the intrinsic electronic properties of these oxidation states.

The electronic structure of f-element complexes is governed by three principal factors: electron–electron repulsion, spin–orbit coupling (SOC), and ligand field effects. These effects dictate the energy and composition of the ground and excited states, which are labeled according to their spin (*S*), orbital angular momentum (*L*), and total angular momentum (*J*) values. The electron–electron repulsion energies are related to the Slater–Condon parameter (F^k^). For f valence shells, F^2^, F^4^, and F^6^ values determine the energy differences between the multiplet terms of the same configuration.^17^ The greatest sensitivity to changes in the ligand field is expressed in the F^2^ term. The identity and oxidation state of the central metal ion play a crucial role in determining the weight of these factors, even with the same electronic configuration, and need to be considered when interpreting spectroscopic data.^74,75^ The interplay of these factors is also used to address the highly debated role of f-electrons in covalency. The relationship between the nephelauxetic effect and covalency^76–78^ provides an interesting avenue to gauge how ligand fields affect the energies and composition of the multiplet structures for a given f-electron configuration. The so-called nephelauxetic reduction shows the reduction of the F^k^ value of an ion when it is in a ligand field environment compared to its value when that same ion is modeled as only a free ion with no ligand field environment, which clearly demonstrates a connection between spectroscopic properties and covalency.

It is widely accepted that lanthanides form predominantly ionic bonds with ligands while actinides tend to show an increased degree of covalency.^79–89^ However, the oxidation state of the ion also plays a major role in determining the covalency of a bond according to Fajans’ rules (i.e., increasing the metal’s oxidation state implies greater metal–ligand covalent interactions).^90,91^ Correlations between these factors are difficult to gauge using only experimental methods, especially with the scarcity of data for heavier actinides. Thus, theoretical methods are needed to model their electronic structures based on these effects and provide a deeper understanding of the fundamental forces that drive the factors that influence spectroscopic properties.

In this study, we used computational models to interrogate the properties that affect the energy and composition of the ground and excited states of four f^7^ ions: Gd^III^, Tb^IV^, Cm^III^, and Bk^IV^. Multiple ligand fields were tested, including aquo complexes ([M^III^(H_2_O)_9_]^3+^, M = Gd and Cm, and [M^IV^(H_2_O)_9_]^4+^, M = Tb and Bk) and three octahedral hexahalide complexes ([M^III^X_6_]^3^^–^, M = Gd and Cm, and [M^IV^X_6_]^2^^–^, M = Tb and Bk; X = F, Cl, Br). While some of these systems are only weakly accessible experimentally, they allow us to gauge the trends exhibited by the series of f^7^ ions in similar ligand field environments with differing ligand polarizability. To provide an additional comparison with these basic ligand fields, the triphenyl arsine oxide chemical environment was also considered ([M^III^(OAsPh_3_)_4_(NO_3_)_2_]^+^, M = Gd and Cm, and [M^IV^(OAsPh_3_)_4_(NO_3_)_2_]^2+^, M = Tb and Bk). This organic ligand was chosen because of its ability to have a relatively polarizable negative charge from the resonance structures retaining the electrostatic affinity as an O-donor ligand. Although phosphine oxide systems are more well-known than arsine oxides,^92,93^ previous work showed that the arsine oxide ligand forms more stable compounds than phosphine oxide ligands. The main goal of this study is to advance our ability to predict chemical properties from absorption and emission spectroscopy for f^7^ systems while providing commentary on the computational methods used to study f-element electronic structures.

## Computational Methods

Electronic structure calculations of f^7^ systems were conducted based on ligand field density functional theory (LFDFT)^94^ as implemented in Amsterdam Density Functional (ADF) version 2023.104.^95,96^ Calculations were performed at the PBE-D3BJ/TZP^97–100^ level of theory (LFDFT-PBE) using COSMO^101^ for the implicit solvent (water) modeling and the zeroth-order regular approximation (ZORA) for the inclusion of spin–orbit coupling. See the Supporting Information (SI) for details on geometry optimizations. Notably, the PBE0-D3BJ functional was also investigated (LFDFT-PBE0), and although it yielded relatively consistent results compared with previous experimental data (Table S3), it was not able to reproduce the expected electronic structure for the Tb^IV^ hexabromide complex (Figure S3, Tables S1–S2). The larger Hartree–Fock (HF) exchange contribution (25%) to the GGA functional in PBE0 produces an artificial inversion of the ligand field which is corrected by lower HF exchange functional such as TPSSh (10%) or a plain GGA functional. Therefore, only results from LFDFT-PBE are shown in the main text, while results from LFDFT-PBE0 are shown in the SI for comparison.

*Ab initio* ligand field theory (AILFT) was also explored via the complete active space self-consistent field (CASSCF) and the *N*-electron valence state second-order perturbation theory (NEVPT2) calculations but showed little to no difference between ligand fields (Figures S1–S2). It has been noted before that only a limited amount of dynamic electron correlation is captured with NEVPT2 calculations only considering the metal-based orbitals and it is difficult to capture the ligand orbitals in a practical active space for an f^7^ species.^71,102^ While the overlocalization of HF and, consequently, post-HF methods such as CASSCF and NEVPT2 can be addressed by approaching the full-CI limit, this is unattainable with the available computational resources and size of the systems. For this analysis, LFDFT becomes a more practical tool as it relies on the minimal active space based on the average-of-configuration (AOC) orbitals to calculate the multiplet structure providing a more transferrable methodology to a broader variety of systems. Due to this, results from AILFT are only discussed further in the SI, but it should be noted that the conclusions drawn are only applicable to the minimal CAS­(7,7) active space.

## Results and Discussion

### Free Ion

To pinpoint the role of 4f vs. 5f valence electrons dictating the optical properties, we first considered the model free ion without including SOC (i.e., spin-free) of Gd^III^, Cm^III^, Tb^IV^, and Bk^IV^. Of the three factors that influence the luminescence of a compound, electron–electron repulsion drives the main splitting of electronic states and can be isolated when looking at a spin-free ion. The f^7^ configuration produces a highly stable ^8^S (*L* = 0, *S* = 7/2) ground state, with excited states being located considerably higher in energy compared with other f^n^ configurations. This higher energy is caused by the high energy required to break the half-filled shell and make spin-flipped configurations that involve electron pairing.

The lowest-lying excited states predicted here when considering only interelectronic repulsion in an f^7^ system include ^6^P (*L* = 1, *S* = 5/2), ^6^I (*L* = 6, *S* = 5/2), and ^6^D (*L* = 2, *S* = 5/2) (Figure 1). The large energy gap that is caused by the separation of a configuration with just unpaired electrons (octuplet) from those with paired electrons makes the f^7^ multiplet structure unique. For simplicity, throughout this article, we refer to the emissive state as the lowest-lying excited state regardless of its composition. In this case, the nature of the emissive state for all four ions is the ^6^P multiplet, as shown in Figure 1 and Table S4. Although interelectronic repulsion is the main electronic effect that f-electrons are subject to, it does not sufficiently provide good insight into the energy gaps of these ions. The energy gaps all fall outside the visible range, which is to say that they are all less than 380 nm (26,315 cm^–1^).

**Figure 1:**
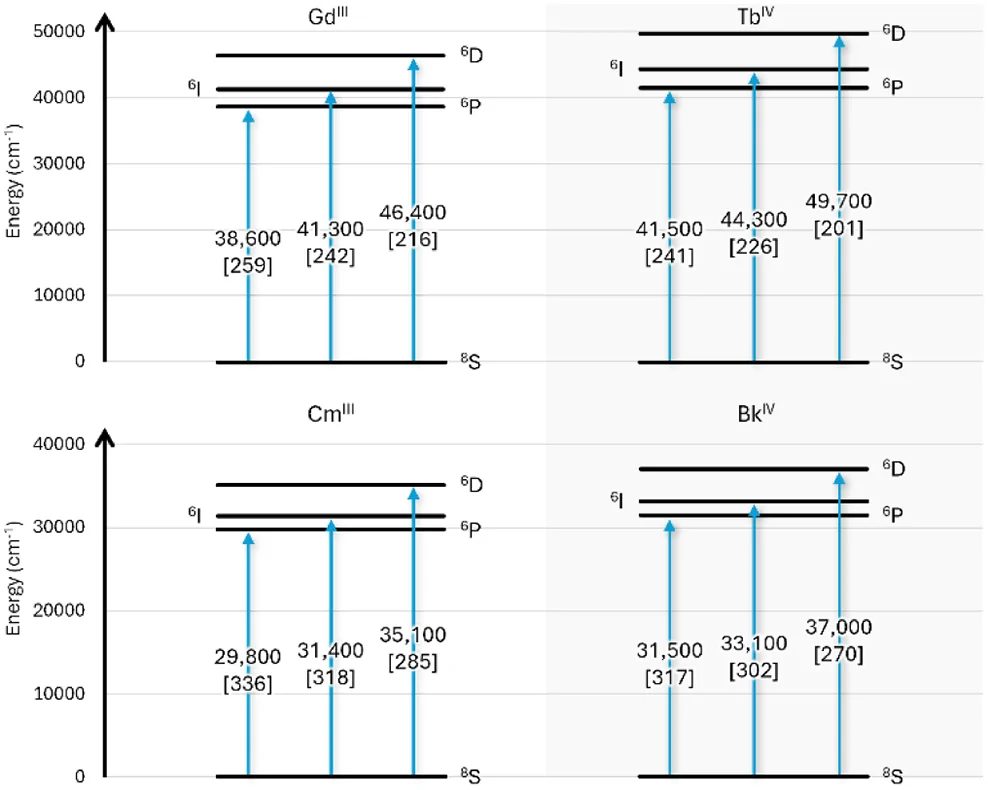
Energy levels of the three lowest-energy spin-free free ion states. Spectroscopic term symbols for the states are shown to the side. Blue arrows indicate energy gap (cm^–1^ on top, nm on bottom in brackets) between ^8^S and the indicated state. Calculated at the LFDFT-PBE level of theory.

Interestingly, the spin-free model alone predicts a lower-energy emissive state for the actinides compared with their lanthanide counterparts (i.e., 29,800 cm^–1^/31,500 cm^–1^ vs. 38,600 cm^–1^/41,500 cm^–1^), with a slight increase moving from the trivalent to the tetravalent ion (Figure 1 and Table S4). This observation is consistent with the radial extent of the 4f/5f shells using the spin-free model ions (Figure 2), in which the lanthanides Gd^III^ and Tb^IV^ have a more contracted valence f-shell, which translates to greater energy gaps for the excited states. Likewise, the difference between 3+ and 4+ ions shows that 4+ ions have slightly more contracted valence f-orbitals in correlation with greater energy ^8^S–^6^P energy gaps (i.e., An^Δ3+/4+^ = 1,700; Ln^Δ3+/4+^ = 2,900 cm^–1^).

**Figure 2:**
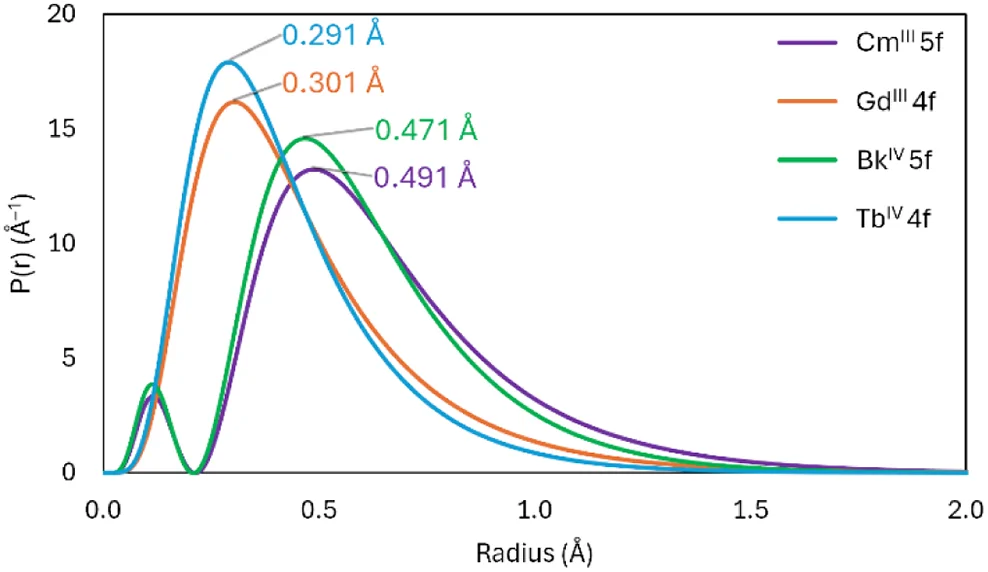
Normalized radial distribution functions (RDFs) for the valence f-electrons in Gd^III^, Tb^IV^, Cm^III^, and Bk^IV^, based on the electron density of the 7 f-orbitals in the free ions (∫P­(r)­dr = 7). Calculated at the DKH2-PBE0/cc-pVTZ-DK3 level of theory.

The energies of the next-highest excited states, ^6^I (41,300 cm^–1^/44,300 cm^–1^ vs. 31,400 cm^–1^/33,100 cm^–1^) and ^6^D (46,400 cm^–1^/49,700 cm^–1^ vs. 35,100 cm^–1^/37,000 cm^–1^), exhibit similarities between actinides and lanthanides, with the actinides having the lower-energy excited states. Upon closer inspection, the gap between the excited states becomes smaller in the actinides than in the lanthanides. For example, the gap between the ^6^I and ^6^P states is 2,700 cm^–1^/2,800 cm^–1^ for Gd^III^/Tb^IV^ and 1,600 cm^–1^/1,600 cm^–1^ Cm^III^/Bk^IV^, suggesting a weaker effect from electron–electron repulsion. Likewise, the Slater–Condon parameter, which can be used to quantify the electron–electron repulsion, becomes lower for the actinides (Table S6). For example, the F^2^ parameter goes from 112,000 cm^–1^/119,700 cm^–1^ in Gd^III^ and Tb^IV^ to 82,500 cm^–1^/86,700 cm^–1^ in Cm^III^ and Bk^IV^.

As mentioned earlier, despite electron–electron repulsion being the largest electronic effect operating in f-element systems, it cannot explain the observed electronic properties of these ions. In the case of Cm^III^, the energy of the emissive state deviates significantly from the well-known orange luminescence commonly observed in many Cm^III^ complexes (∼16,400 cm^–1^ or 610 nm). Based on this model, Cm^III^ would emit in the ultraviolet region (335 nm), indicating that other factors must be included.

The second most prominent factor in influencing the luminescence of a compound is typically SOC.^74^ It describes the direct coupling of orbital angular momentum and spin angular momentum that is predominantly observed in atoms with a large effective nuclear charge. By including first- and second-order SOC, electronic states can further mix and split depending on their *S*, *L*, and *J* values. The SOC ground state for an f^7^ configuration produces only one *J* value of 7/2, with a degeneracy of eight states and potential to mix with other *J* = 7/2 excited states.

This mixing enables multiple Russell–Saunders terms to combine in what is called an intermediate coupling scheme.^103–105^ Intermediate coupling affects the composition of the excited states by combining higher-energy states with lower-energy states (Tables S7–S10), which is similar to the effect seen in actinocene complexes.^106^ Analysis of the ^8^S_7/2_ ground state for the four f^7^ ions shows that the lanthanides Gd^III^ and Tb^IV^ keep consistently pure ground states (^8^S_7/2_ = 97–98%), but the actinides Cm^III^ and Bk^IV^ show a slightly less pure ground state (^8^S_7/2_ = 81–84%). The SOC increases as the effective nuclear charge increases. Naturally, the tetravalent species exert an even greater effective charge on the valence electrons, further increasing the effects of SOC. Likewise, this is reflected in their SOC constant (ζ), which quantifies this feature (i.e., 1,600–1,900 cm^–1^ vs. 3,100–3,700 cm^–1^; Table S6).

As expected, including SOC in the model considerably decreases the energy of the emissive state (Δ*E*_SOC_), with the decrease in energy being significantly greater for the actinides (1,000–1,300 cm^–1^ vs. 3,900–4,900 cm^–1^) because of the relatively smaller amount of shielding from the core electrons compared with the lanthanides (Table S5). The lanthanide species Gd^III^ and Tb^IV^ have a majority of the composition of their emissive state coming from the ^6^P term (75% or more) and no more than three other *J* = 7/2 Russell–Saunders terms contributing more than 5% to one excited state. Conversely, the actinides have up to eight Russell–Saunders terms contributing more than 5% to one excited state (e.g., the first excited state of Bk^IV^) and consistently have more *J*-mixing than their lanthanide counterparts. Between the trivalent and tetravalent species, the mixing also increases, albeit to a lesser extent. For example, the second excited state of the Cm^III^ ion shows a term composition of ^6^P 53% + ^6^D 29% + ^4^D 10% + other 8%, but the same excited state of the Bk^IV^ ion shows a term composition of ^6^P 48% + ^6^D 30% + ^4^D 10% + other 12%, where the ^6^P character decreased by 5%.

If considering the most predominant Russell–Saunders term as the identity of the excited state, then the splitting of the ^6^P and ^6^I multiplets in the actinide free ions seems to overlap. The composition of the third excited state (*E* = 28,014 cm^–1^, *J* = 7/2) in Cm^III^ has majority ^6^I character (i.e., 54%), and the second and fourth excited states (*E* = 26,708 cm^–1^, *J* = 7/2 and *E* = 28,014 cm^–1^, *J* = 3/2) have majority ^6^P character (53% and 66%, respectively). This result starkly contrasts with the lanthanides, which follow the pattern of ^6^P_7/2_, ^6^P_5/2_, and ^6^P_3/2_ in order of increasing energy. The splitting of the ^6^I multiplet from the spin-free scheme in actinides seems to greatly stabilize the ^6^I_7/2_ multiplet—to the point of making it lower in energy than the ^6^P_3/2_ state in pure Russell–Saunders terms.

However, even when considering SOC effects, Cm^III^ is still far away from showing an emissive state (approximately 387 nm) that would emit light in the orange region of the visible light spectrum (585–620 nm). Additionally, the recent report by Nielsen and Sørensen about experimentally determined electronic energy levels of lanthanide­(III) aquo ions shows the emissive state of Gd^III^ at 32,189 cm^–1^,^24^ which is less than that of the free ion with SOC effects included (approximately 37,000 cm^–1^). Considering this, the last of the three factors affecting the electronic structure of f-elements—the ligand field—cannot be neglected to accurately describe and investigate electronic properties of these ions.

### Ligand Field

Because the intrinsic tendency of these f^7^ lanthanide and actinide systems is to have the lowest energy excited states exhibit strong mixing caused by SOC, adding a ligand field acts as a fine-tuner of the electronic structure. This is clearly seen in the emission of Cm in different chemical environments. For this reason, we considered several ligand environments with varying geometries, symmetry, and ligand field strength to show how much these systems can change their low-lying states considering their energy and composition (Figure 3). First, the ground-state ^8^S_7/2_ of each species, regardless of the ligand field applied, remains relatively unchanged for Gd^III^, Tb^IV^, and Cm^III^. For example, in the hexafluoride complex [Gd^III^F_6_]^3–^, the ground state shows 97% contribution coming from the ^8^S terms (Table S22), which is nearly unchanged compared to the 98% contribution exhibited in the ground state of the Gd^III^ free ion. Conversely, the ground state of Bk^IV^ shows much more ligand-field sensitivity. For example, in the hexafluoride complex [Bk^IV^F_6_]^2–^, the ground state shows 67% contribution coming from the ^8^S term (Table S25), which is significantly smaller than the 81% contribution exhibited in the ground state of the Bk^IV^ free ion. The identity of the ligand field seems to drive some of this behavior, with traditionally more covalent ligands instigating a smaller contribution from the ^8^S_7/2_ state. For example, [Bk^IV^Cl_6_]^2–^ and [Bk^IV^Br_6_]^2–^ have 58% and 55% ^8^S contribution, respectively (Tables S29 and S33). Small amounts of splitting for the four Kramers doublets of the ground state are also found for the actinides (i*.*e., up to 26 cm^–1^ for the Cm^III^ complexes and up to 281 cm^–1^ for the Bk^IV^ complexes). Further changes are noted for all species when considering excited states, which vary considerably between the applied ligand fields.

**Figure 3:**
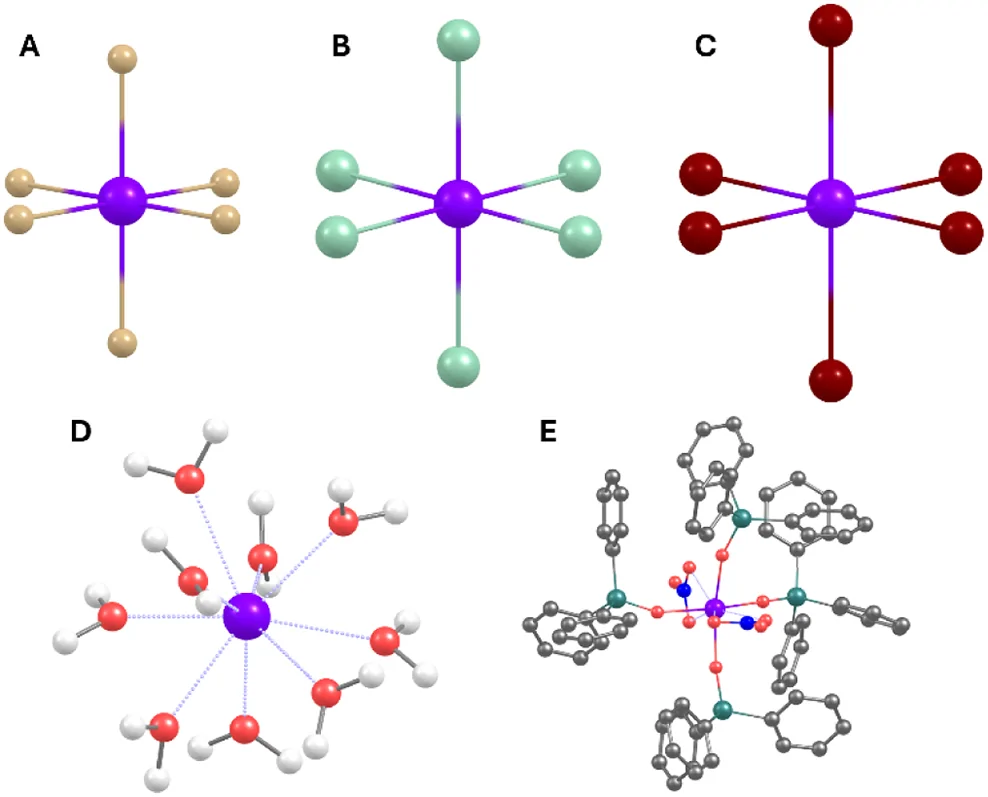
Optimized structures of the (A–C) [Cm^III^X_6_]^3–^ (X = F, Cl, Br), (D) [Cm^III^(H_2_O)_9_]^3+^, and (E) [Cm^III^(OAsPh_3_)_4_(NO_3_)_2_]^+^ complexes. H atoms are removed for clarity in [Cm^III^(OAsPh_3_)_4_(NO_3_)_2_]^+^. Similar structures are calculated for the Gd^III^, Tb^IV^, and Bk^IV^ congeners. See SI for details on optimization level of theory.

### Aquo Ligand Field

One of the simplest cases for a ligand field is the aquo complex. Although experimentally synthesized examples exist for the trivalent complexes, the tetravalent complexes in a water solution remain elusive because of their high oxidation potentials compared with water. Nevertheless, they provide an excellent model for probing ligand field effects. Formally, the aquo complex only features dipole–charge interactions between the water molecule and metal ion, which is why it is sometimes used as a surrogate for the free ion. However, as shown in Figure 4 and Table S5, significant stabilization of the excited states occurs compared with the free ion (Δ*E*_LF_), especially in the tetravalent complexes (approximate values: Gd^III^: 4,700 cm^–1^; Tb^IV^: 20,200 cm^–1^; Cm^III^: 5,300 cm^–1^; Bk^IV^: 14,500 cm^–1^), which greatly increases the wavelength of the emitted light. The stabilization is comparable with what is seen in the hexafluoride complex (*vide infra*). Likewise, a reduction is present in the Slater–Condon parameters, which can be used to quantify the nephelauxetic reduction when compared with the free ion. The nephelauxetic reduction for the tetravalent ions is significantly larger than their trivalent counterparts (e.g., F^2^ = 10.8% Cm^III^ vs. 40.0% Bk^IV^) because of the larger effective charge of the tetravalent ions and drive to obtain the more stable trivalent oxidation state from electron donation (Table S14). Comparison of the lanthanide with actinide aquo complexes shows that Tb^IV^ has a slightly higher nephelauxetic reduction than Bk^IV^ (e.g., F^2^ = 47.1% vs. 40.0%), suggesting that Tb^IV^ is slightly more unstable than Bk^IV^. In all cases, there is an identifiable difference between the free ion and aquo complex, so we advise against using these complexes as a surrogate for the free ion. For the experimentally available Gd^III^ and *Cm* ^III^ aquo complexes energy values for their emissive states differ by 769 cm^–1^ and 1,507 cm^–1^, respectively, from our calculations. Some of the discrepancy may arise from the fact that the coordination number for Gd^III^ and Cm^III^ aquo complexes range from 8 to 9 and another contributing factor could be that we did not explicitly model the second coordination sphere of water molecules.

**Figure 4:**
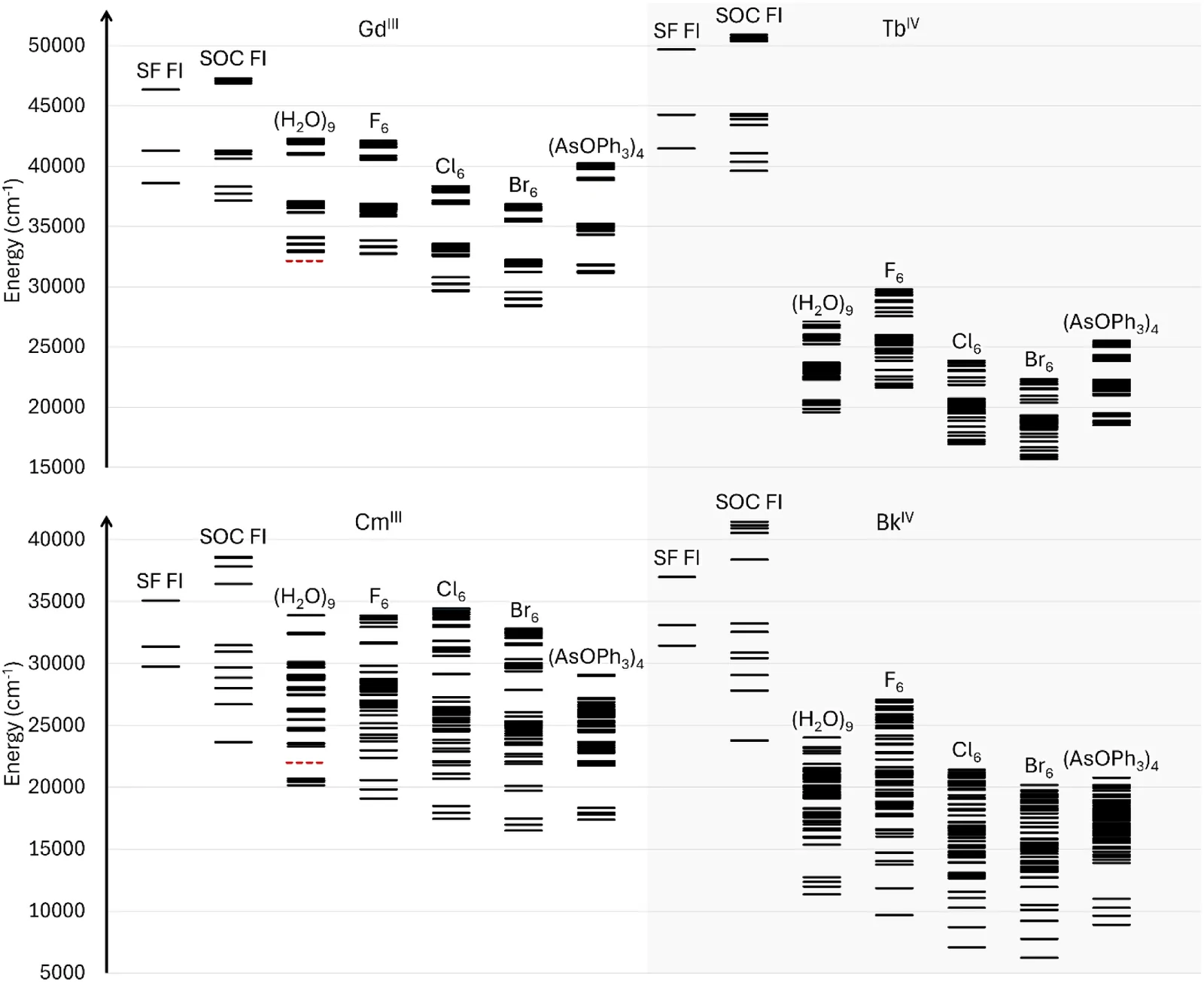
Energy levels of the three lowest-energy spin-free free ion (SF FI) states, 15 lowest-energy SOC free ion (SOC FI) states, and 50 lowest-energy states for the nona-aquo ((H_2_O)_9_), hexafluoride (F_6_), hexachloride (Cl_6_), hexabromide (Br_6_), and tetra-triphenyl arsine oxide (AsOPh_3_)_4_ complexes. Calculated at the LFDFT-PBE level of theory. Experimental values for the Gd^III^ aquo complex^24^ and *Cm* ^III^ aquo complex^107^ are indicated with a red dashed line.

### Hexahalide Ligand Fields

To diversify our study on the effect of ligand field environments, we modeled each lanthanide and actinide in three different octahedral hexahalide environments: hexafluoride, hexachloride, and hexabromide. This model provides an opportunity to note the changes that occur by systematically increasing the polarizability of the ligand. Much like the aquo complex, the stabilization of the excited states and nephelauxetic effect in the tetravalent complexes are significantly greater than their trivalent counterparts. The energy of the emissive state gradually decreases as the polarizability of the ligand increases (e.g., Cm^III^: 19,100 cm^–1^, 17,500 cm^–1^, and 16,500 cm^–1^ for hexafluorides, hexachlorides, and hexabromides, respectively) (Table S4). This gradual energy decrease is consistent for all f^7^ ions considered here, although the tetravalent species have a more significant change between different ligands. For example, the difference between the emissive state energy of hexafluoride and hexachloride complexes for Cm^III^ is approximately 1,900 cm^–1^, but for Bk^IV^, it is 3,100 cm^–1^. The same holds true for the Slater–Condon parameters and SOC constant, along with their nephelauxetic reduction (Tables S11–S13). These observations relate to differential metal–ligand interactions and allow us to connect the observed energy and composition of the emissive state in f^7^ systems with chemical concepts such as covalency. For instance, the stabilization of the lowest-lying multiplet in different halide environments follows Fajans’ rule,^90,91^ which states that the ligand polarization increases as the charge density of the metal increases.

This interaction can be further quantified using the quantum theory of atoms in molecules (QTAIM). The delocalization index (DI), which measures electron-pair sharing between two atomic basins, reveals a clear increase across the Bk­(IV) hexahalide series. Specifically, the DI increases from 0.58 for the hexafluoride to 0.75 and 0.79 for the corresponding hexachloride and hexabromide, respectively. This trend is consistent with the increasing nephelauxetic effect observed across the series. Since electronic factors other than covalency influence the DI values, the correlation between the DI and the nephelauxetic effect should not be interpreted as direct evidence that the observed trend arises exclusively from increased 5f–ligand orbital interactions.

To better understand this correlation, the metal–ligand orbital interactions were further analyzed through the natural localized molecular orbital (NLMO) formalism. Since the ligand systems selected for this study are not subject to backdonation, only β-spin NLMOs are relevant to elucidate the bonding situation (Figures S4–S6 and Table S17). This spin-dependent difference is consistent with the half-filled f^7^ configuration: the occupied α-spin f orbitals are strongly localized and contribute little to metal–ligand bonding, whereas the formally vacant β-spin f orbitals are more readily available to accept electron density from the ligands. For both spin manifolds, the metal contribution increases from the hexafluoride to the hexachloride and then to the hexabromide complexes, consistent with increasing metal–ligand covalency across the series. Within each halide series, the tetravalent ions also exhibit greater metal contributions than their trivalent counterparts. In the β-spin NLMOs, most of this oxidation-state-dependent increase arises from the f-shell contribution, highlighting the increasing role of f orbitals in the bonding of high-valent lanthanide and actinide complexes.

A closely related oxidation-state dependence was identified in the recent AdNDP study of U, Np, and Pu imidophosphorane complexes.^108^ At the PBE level, in the trivalent species, the actinide contributions to the An–N bonding were dominated by 6d character, with populations of 0.096–0.125 |e|, compared with only 0.038–0.063 |e| from the 5f shell. Upon oxidation to the tetravalent state, the 5f contribution increased substantially, producing more comparable 6d and 5f populations of 0.122–0.159 |e| and 0.100–0.158 |e|, respectively. Consequently, the total actinide contribution to these bonding orbitals increased from 0.139–0.191 |e| for An^III^ to 0.221–0.308 |e| for An^IV^. This increase is consistent with the greater metal–ligand covalency expected at higher oxidation states according to Fajans” rules.

### Arsine Oxide Ligand Field

Finally, we used the same methods to predict the qualities of a more complicated system, such as the arsine oxide ligand system [M­(OAsPh_3_)_4_(NO_3_)_2_]^*q*+^ (M = Gd^III^, Cm^III^; *q* = 1; M = Tb^IV^, Bk^IV^; *q* = 2), applied to each of the f^7^ ions (Figure 3E). For complexes with these ligands, the energy of the emissive state and covalency seem to be between the hexafluoride and hexachloride ligands (e.g., Bk^IV^: nephelauxetic reduction of F^2^ = 37.6%, 51.6%, and 47.9% for hexafluoride, hexachloride, and arsine oxide, respectively) (Table S15). The DI shows a similar picture for Bk^IV^ (e.g., DI = 0.58, 0.75, and 0.61 for hexafluoride, hexachloride, and arsine oxide, respectively), as well as Gd^III^. This is not the case for the Tb^IV^ and Cm^III^ complexes, which show that the arsine oxide ligand has a lower DI than the fluoride ligand (Table S16). Notably, the NLMOs also seem to downplay the covalency of the M–O interaction in the complex, with the population on the metal in these interactions being the lowest of all the studied ligand fields (Table S17).

The term mixing of the emissive state of the complexes compared with the free ions (Figure 5) shows a tipping point in the division between lanthanide-like and actinide-like behavior. In Cm^III^ and Bk^IV^, more Russell–Saunders terms are added to the state, the overall contribution from states that contribute less than 5% individually (labeled as “other” in the graph) becomes greater, and the ^6^P term contribution decreases. Among the lanthanides, the effects of the ligand field are practically negligible for Gd^III^ but apparent for Tb^IV^. The ^6^P contribution decreases (75% to 59%), and the contribution from other terms increases (4% to 13%). This result suggests that the behavior of Tb^IV^ complexes is closer to that of the actinides, in terms of intermediate coupling, when compared with Gd^III^.

**Figure 5:**
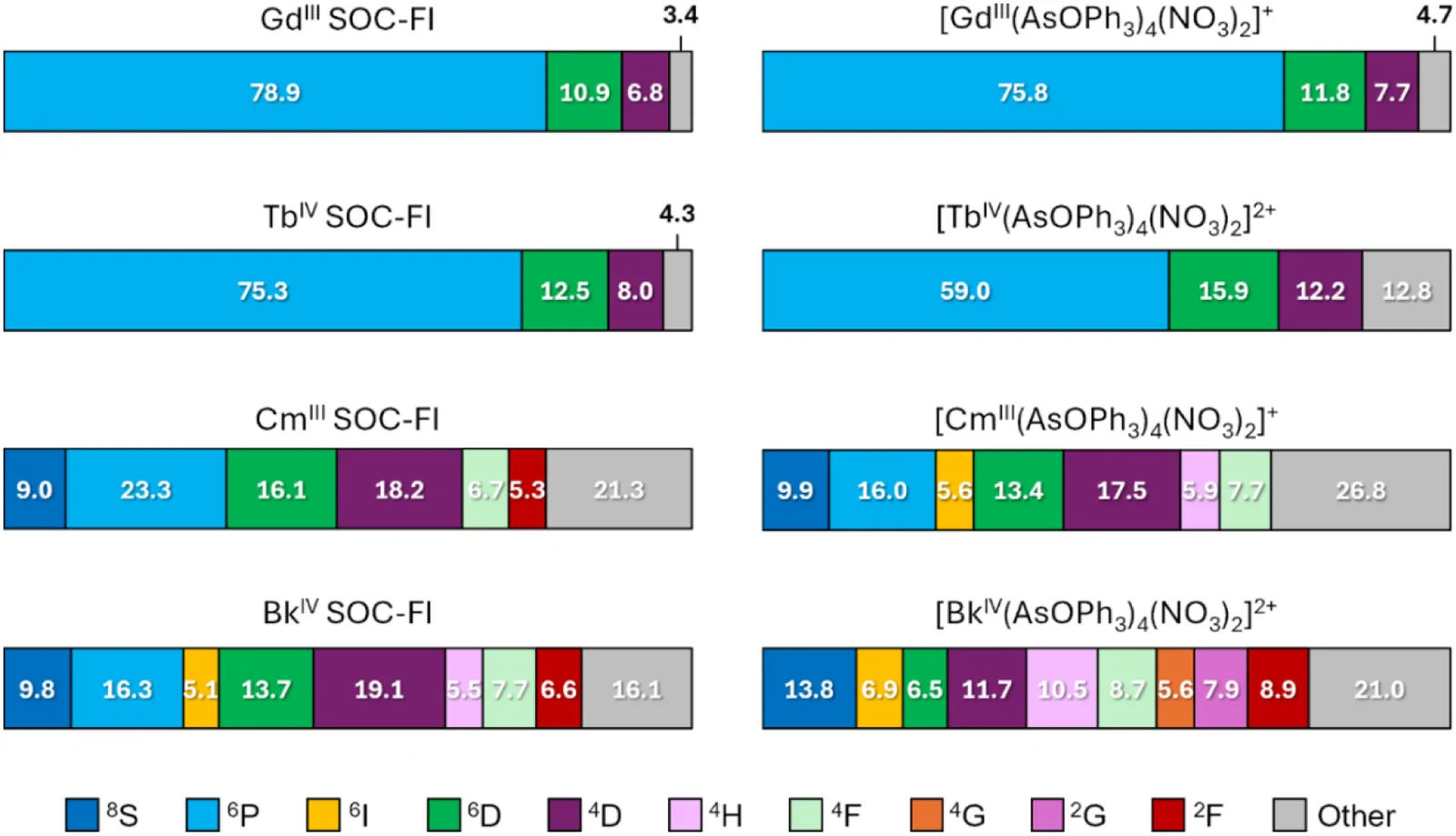
Emissive state Russell–Saunders term composition in arsine oxide complexes. Terms that constitute less than 5% of the overall composition are summed and labeled collectively as “other”. The percentage of each term’s contribution to the overall state is shown within its respective portion of the bar. Calculated at the LFDFT-PBE level of theory.

## Conclusions

Spectroscopic studies of f–f transitions are often complicated by numerous low-lying excited states, yet f^7^ systems offer computational insights into the subtle nuances of excited and emissive states critical for lanthanide and actinide speciation. By modeling f^7^ ions (Gd^III^, Tb^IV^, Cm^III^, Bk^IV^) in a range of ligand fields (aquo, hexahalide, and arsine oxide), we track the evolution of electron–electron repulsion, SOC, and ligand field stabilization through the lens of LFDFT. While similar conclusions were not drawn from the AILFT results, this discrepancy simply arises from the limitations of the minimal (n,7) active space used in the CASSCF/NEVPT2 calculations. However, from a practical standpoint, LFDFT reproduces the experimental behavior of these f^7^ systems particularly well and does so at substantially lower computational cost than CASSCF/NEVPT2 or AILFT calculations. This is not to suggest that LFDFT outperforms CASSCF/NEVPT2, but rather that it provides an accessible and cost-effective route for modeling the electronic structures of more complex f-element systems.

In the free ion case, lanthanides exhibit stronger electron–electron repulsion and weaker SOC compared with actinides, and M^IV^ ions (Tb^IV^, Bk^IV^) show enhanced interactions relative to their M^III^ counterparts (Gd^III^, Cm^III^). Introducing a ligand field generally lowers the energy of the emissive state—an effect more pronounced in M^IV^ species—and increases covalency as the ligand polarizability rises, leading to a reduction in the ground–emissive state energy gap. This reduced gap manifests as a shift in the emission wavelength. The hexahalide complexes via nephelauxetic reduction reveal marked changes in the Slater–Condon parameters and SOC constants, thereby illustrating the sensitivity of electronic structure to ligand covalency. The arsine oxide field produces an intermediate stabilization and covalency compared with hexafluoride and hexachloride fields.

Of particular note, Tb^IV^ in a ligand field displays an intermediate coupling behavior more akin to actinides, with its emissive state composition (formally ^6^P) showing significant contributions from multiple Russell–Saunders terms. This contrasts with the more dominant ^6^P character observed in Gd^III^ complexes. These findings elucidate the delicate interplay between electron interactions and ligand effects in f-element systems as well as encourage further spectroscopic studies and explorations into high-valent f-element complexes to better understand their properties and potential applications.

## Supplementary Material



## Data Availability

The optimized geometries of the complexes are uploaded to a figshare repository which can be accessed via the link: 10.6084/m9.figshare.33010970.

## Notes

Computational methodology details, emissive-state energy, spectroscopic information and Slater–Condon parameters, QTAIM data, and energy and composition of the low-lying excited states for f^7^ species (PDF)

## Abbreviations

SOC: Spin–orbit coupling
DFT: Density functional theory
LFDFT: Ligand field DFT
AILFT: ab initio ligand field theory
