# Supplementary material for: Disentangling Electronic Interactions in f7 Systems: A Comparative Study on Gd III , Tb IV , Cm III , and Bk IV
Source: Inorg Chem. 2026 Sep 14;65(38):22276–86. doi: 10.1021/acs.inorgchem.6c02332 (PMC13630343; doi:10.1021/acs.inorgchem.6c02332)
Supplement: Supplementary file 1 [file ic6c02332_si_001.pdf]

ELECTRONIC SUPPORTING INFORMATION

Disentangling Electronic Interactions in f^7 Systems: A Comparative study on Gd^{III}, Tb^{IV}, Cm^{III}, and Bk^{IV}

Chad M. Studvick,[§] Ivan A. Popov,[‡] and Cristian Celis-Barros*[#]

[§] Department of Chemistry, University of Akron, Akron, Ohio 44325-3601, USA.

[‡] Department of Chemistry, Washington State University, Pullman, Washington 99164, USA.

[#] Radioisotope Science and Technology Division, Oak Ridge National Laboratory, Oak Ridge, TN 37830, USA. E-mail: celisbarroca@ornl.gov

Table of Contents

Additional Details for Computational Methods 1

Computational considerations: CASSCF/NEVPT2 versus LFDFT 3

LFDFT: PBE0 vs. PBE 8

Emissive State Energy 10

Spectroscopic information for f^7 free ions 12

Slater-Condon parameters for f^7 complexes 15

QTAIM data 18

NLMO Data 20

Energy and composition of the low-lying excited states for f^7 complexes 25

Structural Metrics 65

Optimized Geometries 67

References 87

Additional Details for Computational Methods

DFT gas phase optimizations of the hexahalide and aquo models were performed in ORCA version 6.1.0[1,2] using the PBE0 functional[3], SARC-DKH-TZVP basis set[4,5] on lanthanides and actinides, and the DKH-DEF2-TZVP[6] basis set on all other atoms. Scalar relativistic effects were included via the 2-component Douglas–Kroll–Hess (DKH2) Hamiltonian.[7–9] The autoaux[10] feature was used to generate auxiliary basis sets for all atoms and the RIJCOSX approximation[11–13] was used to speed up calculations. Options for no

symmetry constraints, no frozen core, and a tight SCF were used. For the tetra-arsine oxides, a slightly different optimization method was used to better simulate a real solvent environment. Optimizations for the tetra-arsine oxide complexes were performed in ADF 2023.104[14,15] at the PBE-D3BJ/TZP[16–19] level of theory with a COSMO solvation model of acetonitrile to better reflect experimental condition under which it could be synthesized. However, it should be noted that the inclusion of solvent in the optimization has only a minor effect on the overall geometry of the complexes (Tables S41 – S44).

After optimization, CASSCF and NEVPT2 calculations[20,21] were performed on the structures in ORCA 6.1.0 with the same basis sets as previously mentioned. Spin-orbit coupling was included in all calculations using the Douglas-Kroll-Hess (DKH) approximation as implemented in ORCA. The AILFT module[22] with either 4f or 5f electrons was used for Ln and An systems, respectively. All CASSCF and NEVPT2 calculations considered a minimal active space of (n,7), where n is equal to the number of valence f electrons. All multiplicities (8, 6, 4, 2) and all roots (1, 48, 392, 784) were considered for the f^7 systems. All NEVPT2 calculations were performed using the CPCM with water as the solvent.[23,24] LFDFT calculations were performed in ADF 2023.104[14,15] at the ZORA/PBE0/TZP and ZORA/PBE/TZP levels of theory with a degeneracy threshold of 0.0005 eV for excited states. The COSMO[25] solvation model with water was used in all LFDFT calculations. Spin-orbit coupling was included in all calculations using the zeroth-order regular approximation (ZORA) as implemented in ADF.

Natural bond orbital (NBO) analysis was carried out using NBO6[26,27] as implemented in the ADF program. QTAIM[28,29] analysis was performed on all complexes via ADF at the ZORA/PBE/TZP level of theory. Radial distribution function plots were created through the use of Multiwfn.[30] Single point calculations were performed on the free ions at the DKH2-PBE0/cc-pVTZ-DK3 level of theory in Gaussian 16 rev A.03.[31]

Below is the raw input file of a geometry optimization in ORCA of $[Cm^{III}Cl_6]^{3-}$.

```
!RIJCOSX opt freq DKH tightSCF pbe0 DKH-DEF2-TZVP autoaux NoFrozenCore printmos
normalprint notrah UNO

%basis
  newgto Cm
    "SARC-DKH-TZVP"
end
  newauxgto Cm "AutoAux"
end
end

%MaxCore 7500
%pal nprocs 16
end

*xyz -3 8
96     0.000000000     0.000000000     0.000000000
17     0.000000000     2.248502961     0.000000000
17     0.000000000    -2.248502961     0.000000000
17     2.248502970     0.000000000     0.000000000
17    -2.248502970     0.000000000     0.000000000
17     0.000000000     0.000000000    -2.248502961
17     0.000000000     0.000000000     2.248502961
*
```

Computational considerations: CASSCF/NEVPT2 versus LFDFT

Modeling the electronic structures of f-elements is challenging due to the multiconfigurational — and in certain cases multireference — nature of their electronic states. Although CASSCF is the most widely used approach, its computational demands are significant. Moreover, incorporating dynamic correlation and the requirement for larger active spaces escalates the problem from merely challenging to practically infeasible. We deemed it pertinent to compare the LFDFT results with those from CASSCF to address possible methodological limitations or advantages inherent to these two approaches. To compare these two methods on an equal footing, the ab initio ligand field theory (AILFT) via CASSCF/NEVPT2 calculations[32,33] was considered. It is known that post-Hartree Fock methods (such as CASSCF) cannot fully capture the effects of covalency due to the overlocalization of orbitals.[34] LFDFT, on the other hand, does not have the same issue and has shown results that indicate there is more effective penetration of the ligand's electron cloud into that of the metal compared to AILFT.[35] Though, both have proven to be accurate in

modeling experimentally verified Slater-Condon parameters which is ultimately depend on the orbital set used for the ligand field analysis.[36]

To uncover the differences between these theories, we performed calculations on four f^7 configuration free ions (Gd^{III}, Cm^{III}, Tb^{IV}, Bk^{IV}) as well as a series of octahedral hexahalide complexes ($[MX_6]^{3-}$; M = Gd, Cm; $[MX_6]^{2-}$; M = Tb, Bk; X = F, Cl, Br) using CASSCF and NEVPT2 with the minimal (7,7) active space, LFDFT-PBE0, and LFDFT-PBE to test for the influence of Hartree Fock exchange on the spectroscopic properties. For simplicity, we will only discuss the results for the Cm^{III} free ion and hexahalide complexes (Figure S1).

Since the main goal is to understand the states derived from the first excited multiplet in f^7 systems, the attention will be centered around these low-lying states. The simplest splitting is observed in the spin-free free ion (SF FI), where electron-electron repulsion solely dictates the multiplet structure and their energies. As expected, CASSCF and NEVPT2 calculations show a significant energy difference (ΔE_{DC} = 4,033 cm^{-1}) in the ^6P state coming from the inclusion of dynamic correlation, E_{DC} (Table S5). Interestingly, the LFDFT results show this state even higher in energy than the CASSCF results, which could be due to the absence of a ligand field environment.

The addition of spin-orbit coupling to the free ion (SOC FI) allows the ^6P state to mix and split with higher spin free states with the same J-value, J = 7/2. This stabilizes the energy of the emissive state and decreases the gap with the ground state. If we consider the stabilization of the split multiplet (ΔE_{SOC}) with respect to the SF FI, the energy difference is slightly greater for LFDFT compared to the multireference results (3800-3900 vs. 3300-3400 cm^{-1}). A significantly greater difference is observed in the decrease in energy of the lowest-lying SOC FI state brought about by the ligand field splitting (ΔE_{LF}). This also correlates with the reduction of the Slater-Condon parameters due to the nephelauxetic effect, which is much smaller in the multireference results compared to the LFDFT results (Figure S2). It is noteworthy that the Slater-Condon parameters in CASSCF results are much higher in energy than NEVPT2 results even in the free ion (i.e., F^2 = 80,200 cm^{-1} *vs.* 62,300 cm^{-1}), likely due to the stabilization offered by dynamic correlation. Although both NEVPT2 and LFDFT provide similar results in the molecular system, the recovery of electronic effects are completely different. While NEVPT2 corrects the energy and composition of the electronic states by inclusion of dynamic correlation to the free ion, the LFDFT stabilizes the electronic states due to the interactions with the chemical environment of the metal center.

This difference is better understood when considering the other halide systems. Chemical intuition suggests that an increase in covalency moving from fluoride to chloride, and then to bromide should be expected. This would decrease the interelectronic repulsion of f-electrons which ultimately should reflect in a gradual decrease of the Slater-Condon parameters from lighter to heavier halides. However, CASSCF and NEVPT2 fail to reflect any significant difference between the ligand field environments, whereas LFDFT results show considerable differences between them (Tables S11–S13). Overall, these results suggest that CASSCF and NEVPT2 struggle to capture fundamental ligand field effects when a minimal (n,7) active space is used. This can be only partially solved by expanding the active space, which would necessitate a higher computational cost. With the computational cost of LFDFT being lower than CASSCF and NEVPT2 even at the minimal active space, it appears to be the best choice for the analysis of these systems.

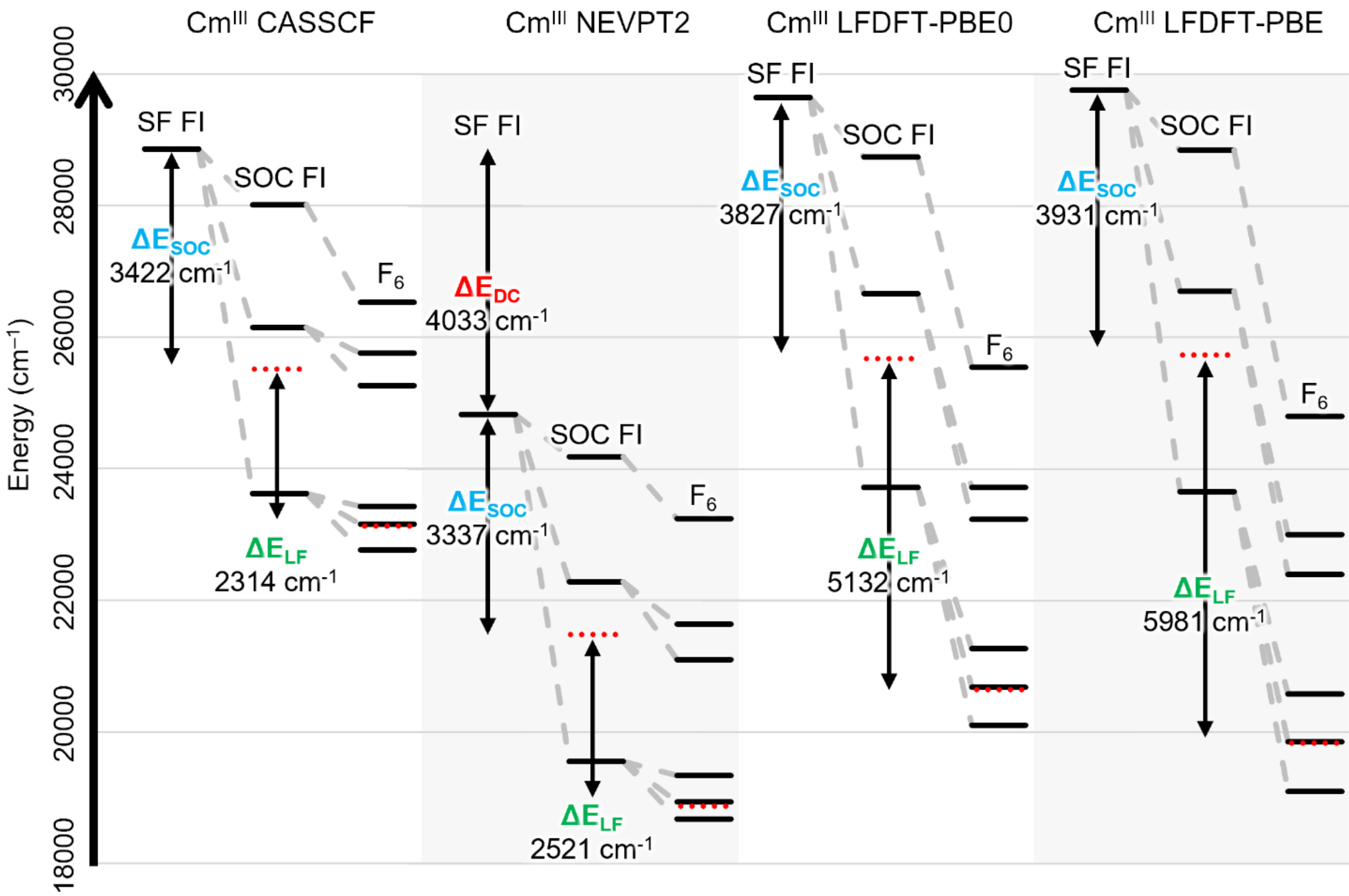

Figure S1. Comparison of CASSCF, NEVPT2, LFDFT-PBE0, and LFDFT-PBE results for the spin-free free ion (SF FI), spin-orbit coupled free ion (SOC FI), and hexafluoride complex (F_6) of Cm^{III}. The barycenter of the spin-orbit coupled states is shown with the dotted red line. The change in energy due to spin-orbit coupling (ΔE_{SOC}), the change in energy due to dynamic correlation (ΔE_{DC}), and the change in energy due to the ligand field (ΔE_{LF}) are show for each level of theory. See Table S4 for the exact energy values of these states.

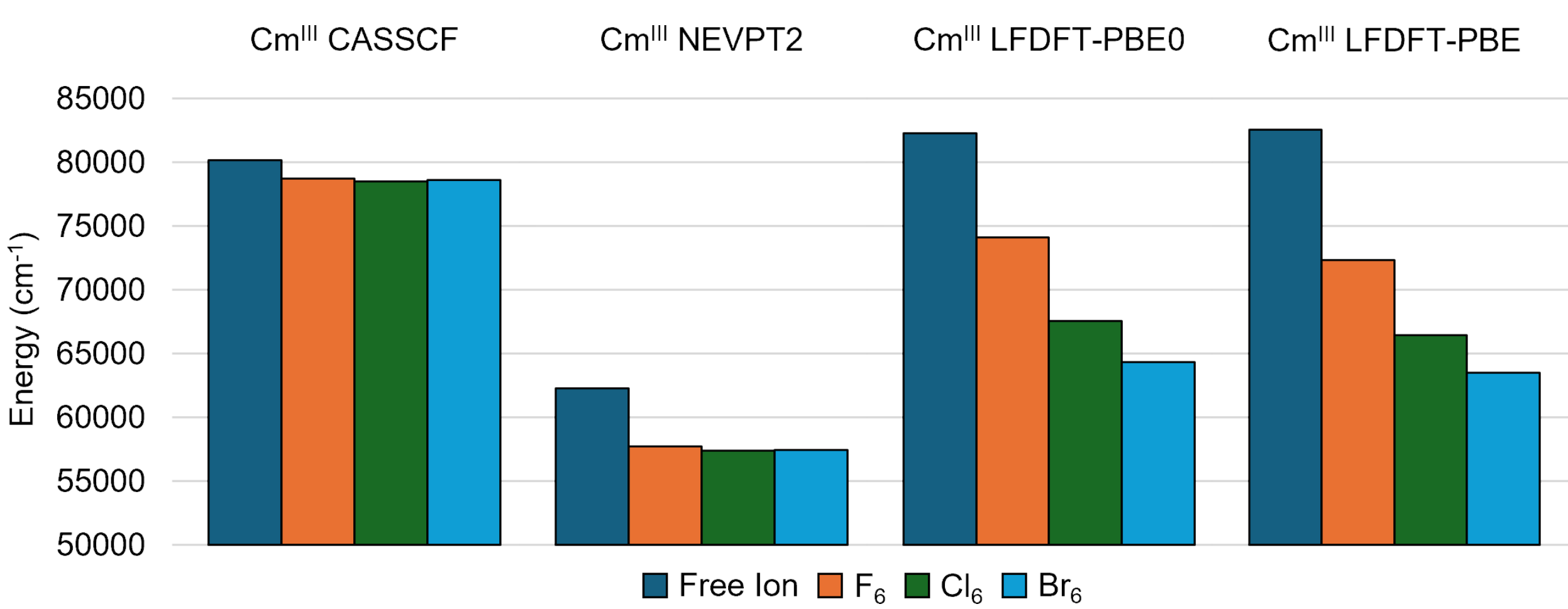

Figure S2. Bar graph of the F^2 Slater-Condon parameter for Cm^{III} free ion and hexahalides at different levels of theory. See Tables S6 and S11-S13 for exact energy values.

Below is the raw input file of a CASSCF/NEVPT2 calculation in ORCA of $[Cm^{III}Cl_6]^{3-}$.

```
!RIJCOSX DKH tightSCF DKH-DEF2-TZVP autoaux printmos normalprint notrah cpcm(water)
!moread
%moinp "CmIII_hexachloride.qro"

%rel
  method DKH
  order 2
  Picturechange true
end

%scf
  UHFNO true
  TolG 1.0e-04
  TolE 1.0e-06
  maxiter 500
end

%casscf
  printwf true
  nel 7
  norb 7
  mult 8,6,4,2
  nroots 1,48,392,784
  PTMethod SC_NEVPT2
  PTSettings
  D4Tpre 1e-12
  D4Step efficient
  end
  LFTCase 5f
  rel
  dosoc true
  printlevel 3
  end
  etol 1e-6
  gtol 1e-4
  maxiter 300
  trafostep ri
end

%basis
 newgto Cm
  "SARC-DKH-TZVP"
```

```
end
  newauxgto Cm "AutoAux"
end
end

%MaxCore 7500
%pal nprocs 16
        end

*xyzfile -3 8 CmIII_hexachloride.xyz
```

Below is the raw input file of a LFDFT calculation in ADF of $[Tb^{IV}Br_6]^{3-}$.

```
Task SinglePoint
System
  Atoms
    Tb 0.0 0.0 0.0 adf.R=2.250
    Br 1e-06 2.712317 2e-06 adf.R=1.850
    Br -2e-06 -2.712318 -2e-06 adf.R=1.850
    Br 2.712319 -1e-06 0.0 adf.R=1.850
    Br -2.712317 -5e-06 2e-06 adf.R=1.850
    Br -1e-06 6e-06 2.712317 adf.R=1.850
    Br 1e-06 2e-06 -2.712318 adf.R=1.850
  End
End

Engine ADF
  Basis
    Type TZP
    Core None
  End
  XC
    GGA PBE
    DISPERSION GRIMME3 BJDAMP
  End
  Symmetry NOSYM
  Solvation
    Surf Delley
    Solv name=Water cav0=0.0 cav1=0.0067639
    Charged method=CONJ
    C-Mat POT
    SCF VAR ALL
    CSMRSP
  End
```

```
  Irrepoccupations
    A 270 1 1 1 1 1 1 1
  End
EndEngine
eor

"$AMSBIN/lfdft" << eor
  nshel 1
  nlval 4 3
  MOind 136 137 138 139 140 141 142
  soc   1
eor
```

LFDFT: PBE0 vs. PBE

Within LFDFT, the two functionals chosen, PBE and PBE0, only show a slight difference in their ΔE_{LF} values (5,100 vs. 6,000 cm^{-1}), with PBE having the greater stabilization. For experimental validation of the LFDFT methods, we took examples from three different Cm^{III} complexes and used both LFDFT-PBE0 and LFDFT-PBE to calculate the energy of the emissive state (Table S3). Both functionals are accurate (reproducing energies within 60 nm), with PBE consistently predicting slightly longer wavelengths than PBE0. However, the PBE0 method fails to reproduce the expected trends in the molecular orbital energy ordering in Tb^{IV} hexahalides, precluding its use as our primary method of analysis. An octahedral f-block element complex is expected to have an ordering of orbital energies with a single A_{2u} orbital as the lowest in energy, triply degenerate T_{2u} orbitals as intermediate in energy to the lowest and highest energy orbitals, and triply degenerate T_{1g} orbitals as the highest in energy.[33] This is correctly seen when analyzing the Tb^{IV} hexabromide complex, $[Tb^{IV}Br_6]^{2-}$, and the Bk^{IV} hexabromide complex, $[Bk^{IV}Br_6]^{2-}$ using the PBE functional. However, the amount of E_{HF} allowed in the hybrid functional can affect this ordering (Figure S3). For an additional comparison point, we included calculations performed with the TPSSh functional (E_{HF} = 10%) as it has an E_{HF} less than PBE0 (E_{HF} = 25%). While TPSSh shows an increase in energy of the A_{2u} and T_{2u} orbitals and a decrease in energy for the T_{1g} orbitals in both $[Tb^{IV}Br_6]^{2-}$ and $[Bk^{IV}Br_6]^{2-}$, maintaining the same orbital energy ordering, the PBE0 functional shows a different orbital energy ordering only for the Tb^{IV} complex.

Furthermore, it appears that the composition of the molecular orbitals is also affected by this reordering of the relative orbital energies, with the T_{2u} orbital calculated using the PBE0 functional showing only ~50% 4f character, suggesting mixing with the Br orbitals, while the same symmetry orbital calculated using the PBE functional shows > 80% 4f character (Table S1). The Russell-Saunders composition of the multiplets for $[Tb^{IV}Br_6]^{2-}$ is also affected by the functional used. The PBE0 functional shows 7% less ^6P character and 14% more ^6I character within the first excited state, with several other Russell-Saunders terms also showing different contributions (Table S2).

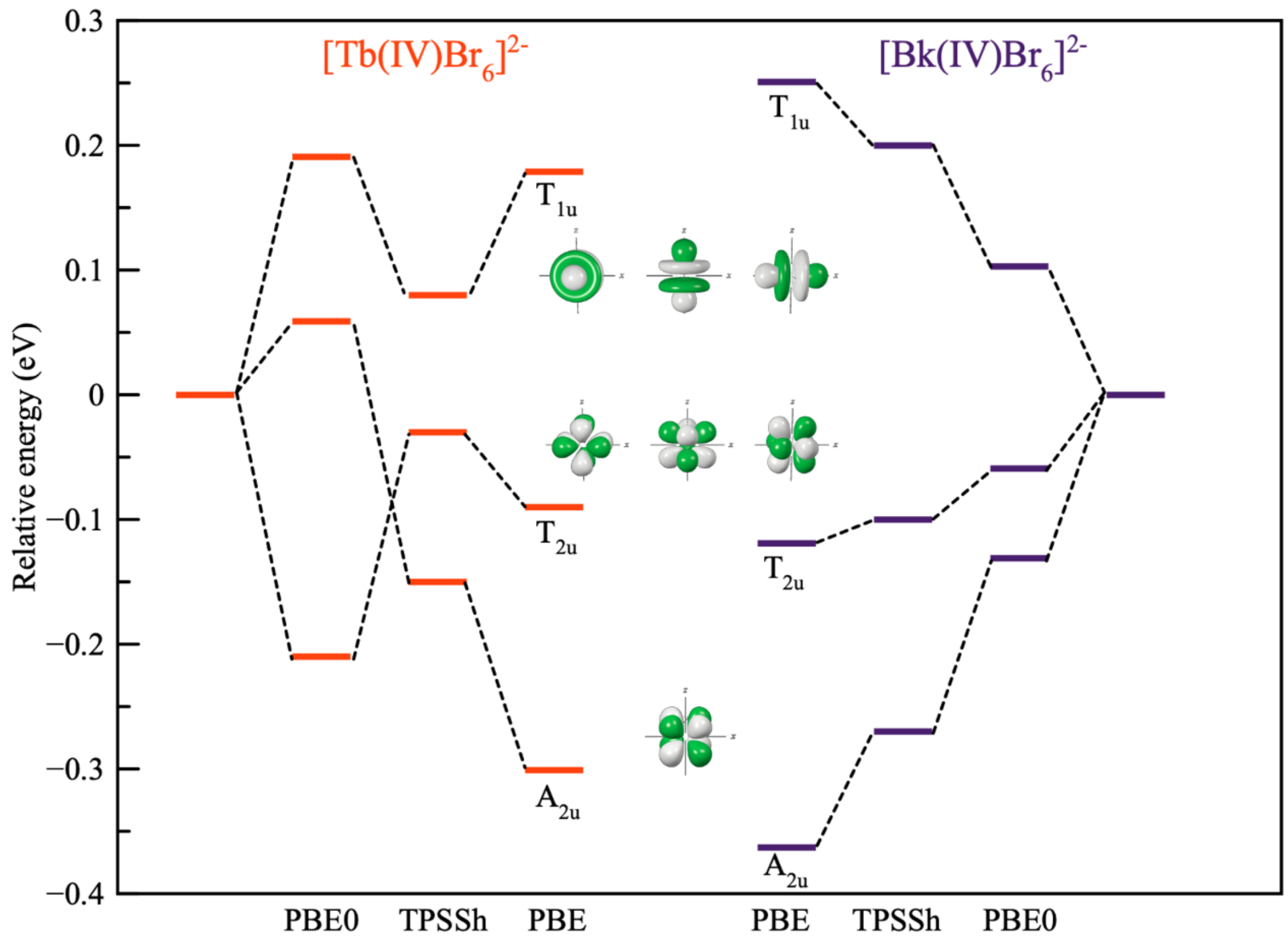

Figure S3. Molecular orbital energy diagram for $[Tb^{IV}Br_6]^{2-}$ and $[Bk^{IV}Br_6]^{2-}$ using the PBE, TPSSh, and PBE0 functionals. Each calculation also included D3 dispersion corrections with Becke-Johnson damping (BJ). The energies are presented as being relative to the barycenter of the PBE0 calcualtions.

Table S1. Relative orbital energies (E; eV), octahedral ligand-field splitting (Symm.) and composition (Comp.) for the complex, $[Tb^{IV}Br_6]^{2-}$, calculated with the PBE, TPSSh, and PBE0 functionals. The splitting reflects the LFDFT splitting.

**PBE**			**TPSSh**			**PBE0**		
Symm.	E (eV)	Comp.	Symm.	E (eV)	Comp.	Symm.	E (eV)	Comp.
A_{2u}	0.00	100% 4f	A_{2u}	0.00	100% 4f	T_{2u}	0.00	52% 4f
T_{2u}	0.21	82% 4f	T_{2u}	0.12	86% 4f	A_{2u}	0.27	100% 4f
T_{1u}	0.48	71% 4f	T_{1u}	0.23	67% 4f	T_{1u}	0.40	93% 4f

Table S2. Orbital energy (E; cm^{-1}) and Russell-Saunders composition (Comp.) for the ground and first excited multiplits calculations for the complex, $[Tb^{IV}Br_6]^{2-}$, using LFDFT with the PBE, TPSSh, and PBE0 functionals.

**PBE**	**TPSSh**	**PBE0**

E (cm^{-1})	Comp.	E (cm^{-1})	Comp.	E (cm^{-1})	Comp.
0	93% 8S; 6% 6P	0	93% 8S; 6% 6P	0	93% 8S; 6% 6P
15,707	48% 6P; 17% 6D; 13% 4D; 4% 8S; 4% 6I; 3% 4F	16,276	41% 6P; 10% 6D; 12% 4D; 4% 8S; 18% 6I; 2% 4F	14,272	41% 6P; 18% 6I; 12% 4D; 10% 6D; 4% 8S; 3% 4H

Emissive State Energy

Table S3. Comparison of experimentally determined emissive state energy (give in wavelength nm) with PBE0 and PBE LFDFT results.

	**Exp.**	**LFDFT-PBE0**	**LFDFT-PBE**	**Ref**
**$[Cm^{III}H_2O_9]^{3+}$**	455	474	490	[37]
**$Cm(S_2CNEt_2)_3(N_2C_{12}H_8)$**	611	622	667	[38]
**Cm^{III} Terpy 1:1**	614	542	606	[39]

Table S4. Energy of the lowest lying excited state (cm^{-1}) in the **free ion** f^7 elements, **aquo**, **hexahalide**, and **tetra-arsine oxide** complexes. The top text corresponds to LFDFT-PBE results, the text in parentheses to LFDFT-PBE0, the text in square brackets to CASSCF, and the text in curly braces to NEVPT2. Experimental values are written in italics when available. Likewise, the LFDFT-PBE0 calculations for Tb^{IV} with the tetra-arsine oxide ligand could not converge properly, so the values are omitted here and elsewhere in the SI.

	**Gd^{III}**	**Tb^{IV}**	**Cm^{III}**	**Bk^{IV}**
**Free Ion Spin-Free**	38,597<br>(38,409)<br>[39,106]<br>{35,789}	41,451<br>(41,362)<br>[41,683]<br>{38,540}	29,758<br>(29,648)<br>[28,864]<br>{24,831}	31,463<br>(31,443)<br>[31,664]<br>{27,585}
**Free Ion SOC**	37,156<br>(36,998)<br>[37,952]<br>{34,675}	39,593<br>(39,536)<br>[40,374]<br>{37,285}	23,655<br>(23,724)<br>[23,621]<br>{19,554}	23,792<br>(23,978)<br>[24,935]<br>{20,858}
**$[M(H_2O)_9]^q$**	32,885<br>(33,816)<br>*32,189*[a]	19,593<br>(-)	20,181<br>(20,934)<br>*21,978*[b]	11,343<br>(12,767)
**$[MF_6]^q$**	32,718<br>(33,416)<br>[37,516]<br>{34,073}	21,634<br>(22,951)<br>[39,949]<br>{36,310}	19,100<br>(20,104)<br>[22,762]<br>{18,678}	9,663<br>(11,738)<br>[22,365]<br>{18,483}

**[MCl_6]^q**	29,655<br>(29,922)<br>[37,522]<br>{34,119}	16,957<br>(16,677)<br>[39,984]<br>{36,524}	17,471<br>(18,250)<br>[22,955]<br>{18,712}	7,060<br>(8,525)<br>[23,938]<br>{18,767}
**[MBr_6]^q**	28,420<br>(28,466)<br>[37,536]<br>{34,138}	15,708<br>(14,273)<br>[40,000]<br>{36,591}	16,515<br>(17,236)<br>[23,023]<br>{18,644}	6,227<br>(7,755)<br>[24,075]<br>{18,775}
**[$M(AsOPh_3)_4(NO_3)$]^q**	31,167<br>(32,045)	18,527<br>(-)	17,398<br>(18,661)	8,886<br>(10,329)

a. See ref [40]
b. See ref [37]

Table S5. Change in energy of the emissive state's barycenter (cm^{-1}) between spin free free ion and spin-orbit coupling free ion calculations (ΔE_{SOC}), spin-orbit coupling free ion and various ligand fields (ΔE_{LF}), and CASSCF and NEVPT2 calculations which accounts for the dynamic correlation in the system (ΔE_{DC}). The top text corresponds to LFDFT-PBE results, the text in parentheses to LFDFT-PBE0, the text in square brackets to CASSCF, and the text in curly braces to NEVPT2.

	**Gd^{III}**	**Tb^{IV}**	**Cm^{III}**	**Bk^{IV}**
**ΔE_{SOC} (Free Ion)**	988<br>(969)<br>[791]<br>{749}	1,276<br>(1,254)<br>[896]<br>{841}	3,931<br>(3,827)<br>[3,422]<br>{3,337}	4,852<br>(4,738)<br>[4,552]<br>{4,203}
**ΔE_{DC} Free Ion**	3,317	3,143	4,033	4,080
**ΔE_{LF} ([$M(H_2O)_9$]^q)**	4,650<br>(3,584)	20,198<br>(-)	5,349<br>(4,639)	14,506<br>(13,312)
**ΔE_{LF} ([MF_6]^q)**	4,855<br>(4,004)<br>[798]<br>{965}	18,372<br>(17,096)<br>[829]<br>{1,373}	5,981<br>(5,132)<br>[2,314]<br>{2,521}	14,832<br>(13,401)<br>[2,989]<br>{4,205}
**ΔE_{LF} ([MCl_6]^q)**	7,923<br>(7,519)<br>[793]<br>{920}	23,041<br>(23,298)<br>[798]<br>{1,166}	7,863<br>(7,271)<br>[2,350]<br>{2,487}	17,930<br>(17,371)<br>[2,828]<br>{4,147}
**ΔE_{LF} ([MBr_6]^q)**	9,157<br>(8,966)<br>[778]<br>{902}	24,283<br>(25,575)<br>[783]<br>{1,100}	8,844<br>(8,351)<br>[2,326]<br>{2,706}	18,874<br>(18,542)<br>[2,779]<br>{4,258}

**ΔE_{LF}**<br>**($[M(AsOPh_3)_4(NO_3)_2]^q$)**	6,369<br>(5,343)	21,459<br>(-)	7,960<br>(6,758)	16,673<br>(15,647)

Spectroscopic information for f^7 free ions

Table S6. Slater-Condon parameters (cm^{-1}), F^2, F^4, and F^6 describing 4f-4f/5f-5f inter-electronic repulsion, and spin-orbit coupling constant (cm^{-1}), ζ for each of the **free ion** f^7 elements. The top text corresponds to LFDFT-PBE results, the text in parentheses to LFDFT-PBE0, the text in square brackets to CASSCF, and the text in curly braces to NEVPT2.

	F^2	F^4	F^6	ζ
**Gd^{III}**	112,047<br>(111,547)<br>[113,770]<br>{97,800}	70,027<br>(69,687)<br>[71,276]<br>{63,750}	50,295<br>(50,087)<br>[50,799]<br>{50,265}	1,595<br>(1,613)<br>[1,430]<br>{-}
**Tb^{IV}**	119,651<br>(119,371)<br>[121,213]<br>{106,894}	75,200<br>(75,010)<br>[76,194]<br>{69,253}	54,128<br>(54,040)<br>[54,073]<br>{53,882}	1,887<br>(1,855)<br>[1,579]<br>{-}
**Cm^{III}**	82,549<br>(82,269)<br>[80,158]<br>{62,279}	53,813<br>(53,636)<br>[52,206]<br>{43,966}	39,471<br>(39,360)<br>[38,267]<br>{37,137}	3,140<br>(3,065)<br>[2,843]<br>{-}
**Bk^{IV}**	86,723<br>(86,705)<br>[87,295]<br>{69,307}	56,867<br>(56,862)<br>[57,236]<br>{48,937}	41,835<br>(41,780)<br>[42,097]<br>{40,934}	3,659<br>(3,630)<br>[3,400]<br>{-}

Table S7. Energy and composition of the 15 lowest energy excited states of the free ion with spin orbit coupling for **Gd^{III}**. Energy values are in cm^{-1}. The top text corresponds to LFDFT-PBE results and the text in parentheses to LFDFT-PBE0.

E (cm^{-1})	J	^{2S+1}L Composition
0<br>(0)	3.5<br>(3.5)	^{8}S 98% + Other 2%<br>(^{8}S 98% + Other 2%)
37,156<br>(36,998)	3.5<br>(3.5)	^{6}P 79% + ^{6}D 11% + ^{4}D 7% + Other 3%<br>(^{6}P 79% + ^{6}D 11% + ^{4}D 7% + Other 3%)
37,744<br>(37,574)	2.5<br>(2.5)	^{6}P 86% + ^{6}D 11% + Other 3%<br>(^{6}P 86% + ^{6}D 11% + Other 3%)
38,309<br>(38,127)	1.5<br>(1.5)	^{6}P 92% + ^{6}D 6% + Other 2%<br>(^{6}P 92% + ^{6}D 6% + Other 2%)
40,629<br>(40,444)	3.5<br>(3.5)	^{6}I 93% + ^{4}H 6% + Other 1%<br>(^{6}I 94% + ^{4}H 6% + Other 1%)

41,005<br>(40,811)	4.5<br>(4.5)	^{6}I 95% + Other 5%<br>(^{6}I 95% + Other 5%)
41,224<br>(41,026)	8.5<br>(8.5)	^{6}I 96% + Other 4%<br>(^{6}I 96% + Other 4%)
41,318<br>(41,118)	5.5<br>(5.5)	^{6}I 97% + Other 3%<br>(^{6}I 97% + Other 3%)
41,512<br>(41,308)	6.5<br>(6.5)	^{6}I 98% + Other 2%<br>(^{6}I 98% + Other 2%)
41,519<br>(41,315)	7.5<br>(7.5)	^{6}I 98% + Other 2%<br>(^{6}I 98% + Other 2%)
46,042<br>(45,825)	4.5<br>(4.5)	^{6}D 94% + Other 6%<br>(^{6}D 94% + Other 6%)
46,885<br>(46,649)	0.5<br>(0.5)	^{6}D 99% + Other 1%<br>(^{6}D 99% + Other 1%)
47,034<br>(46,798)	3.5<br>(3.5)	^{6}P 10% + ^{6}D 83% + Other 7%<br>(^{6}P 10% + ^{6}D 83% + Other 7%)
47,140<br>(46,900)	1.5<br>(1.5)	^{6}P 6% + ^{6}D 91% + Other 3%<br>(^{6}P 6% + ^{6}D 91% + Other 2%)
47,296<br>(47,054)	2.5<br>(2.5)	^{6}P 12% + ^{6}D 84% + Other 4%<br>(^{6}P 11% + ^{6}D 84% + Other 4%)

Table S8. Energy and composition of the 15 lowest energy excited states of the free ion with spin orbit coupling for **Tb^{IV}**. Energy values are in cm^{-1}. The top text corresponds to LFDFT-PBE results and the text in parentheses to LFDFT-PBE0.

E (cm^{-1})	J	$^{2S+1}$L Composition
0<br>(0)	3.5<br>(3.5)	^{8}S 97% + Other 3%<br>(^{8}S 97% + Other 3%)
39,593<br>(39,536)	3.5<br>(3.5)	^{6}P 75% + ^{6}D 12% + ^{4}D 8% + Other 4%<br>(^{6}P 76% + ^{6}D 12% + ^{4}D 8% + Other 4%)
40,352<br>(40,281)	2.5<br>(2.5)	^{6}P 83% + ^{6}D 13% + Other 3%<br>(^{6}P 84% + ^{6}D 13% + Other 3%)
41,077<br>(40,994)	1.5<br>(1.5)	^{6}P 90% + ^{6}D 8% + Other 2%<br>(^{6}P 90% + ^{6}D 8% + Other 2%)
43,395<br>(43,317)	3.5<br>(3.5)	^{6}I 92% + ^{4}H 7% + Other 1%<br>(^{6}I 92% + ^{4}H 7% + Other 1%)
43,889<br>(43,802)	4.5<br>(4.5)	^{6}I 94% + Other 6%<br>(^{6}I 94% + Other 6%)
44,175<br>(44,082)	8.5<br>(8.5)	^{6}I 96% + Other 4%<br>(^{6}I 96% + Other 4%)
44,298<br>(44,202)	5.5<br>(5.5)	^{6}I 96% + Other 4%<br>(^{6}I 96% + Other 4%)
44,548<br>(44,448)	6.5<br>(6.5)	^{6}I 97% + Other 3%<br>(^{6}I 97% + Other 3%)
44,559<br>(44,458)	7.5<br>(7.5)	^{6}I 97% + Other 3%<br>(^{6}I 97% + Other 3%)

49,259<br>(49,161)	4.5<br>(4.5)	^{6}D 92% + Other 8%<br>(^{6}D 93% + Other 7%)
50,362<br>(50,243)	0.5<br>(0.5)	^{6}D 99% + Other 1%<br>(^{6}D 99% + Other 1%)
50,528<br>(50,409)	3.5<br>(3.5)	^{6}P 12% + ^{6}D 80% + Other 8%<br>(^{6}P 12% + ^{6}D 80% + Other 8%)
50,693<br>(50,568)	1.5<br>(1.5)	^{6}P 8% + ^{6}D 89% + Other 3%<br>(^{6}P 8% + ^{6}D 89% + Other 3%)
50,884<br>(50,757)	2.5<br>(2.5)	^{6}P 13% + ^{6}D 81% + Other 5%<br>(^{6}P 13% + ^{6}D 81% + Other 5%)

Table S9. Energy and composition of the 15 lowest energy excited states of the free ion with spin orbit coupling for **Cm^{III}**. Energy values are in cm^{-1}. The top text corresponds to LFDFT-PBE results and the text in parentheses to LFDFT-PBE0.

E (cm^{-1})	J	$^{2S+1}$L Composition
0<br>(0)	3.5<br>(3.5)	^{8}S 84% + ^{6}P 14% + Other 2%<br>(^{8}S 85% + ^{6}P 13% + Other 2%)
23,655<br>(23,724)	3.5<br>(3.5)	^{8}S 9% + ^{6}P 23% + ^{6}D 16% + ^{4}D 20% + ^{4}F 7% + ^{2}F 5% + Other 20%<br>(^{8}S 9% + ^{6}P 24% + ^{6}D 16% + ^{4}D 20% + ^{4}F 7% + ^{2}F 5% + Other 19%)
26,708<br>(26,664)	2.5<br>(2.5)	^{6}P 53% + ^{6}D 29% + ^{4}D 10% + Other 8%<br>(^{6}P 53% + ^{6}D 29% + ^{4}D 10% + Other 8%)
28,014<br>(27,986)	3.5<br>(3.5)	^{6}P 6% + ^{6}I 54% + ^{4}H 23% + Other 16%<br>(^{6}P 6% + ^{6}I 55% + ^{4}H 23% + Other 16%)
28,847<br>(28,748)	1.5<br>(1.5)	^{6}P 66% + ^{6}D 21% + ^{4}S 6% + Other 6%<br>(^{6}P 67% + ^{6}D 21% + ^{4}S 6% + Other 6%)
29,700<br>(29,649)	4.5<br>(4.5)	^{6}I 63% + ^{4}H 19% + Other 18%<br>(^{6}I 64% + ^{4}H 19% + Other 17%)
30,950<br>(30,852)	8.5<br>(8.5)	^{6}I 76% + ^{4}K 19% + Other 5%<br>(^{6}I 77% + ^{4}K 18% + Other 5%)
31,475<br>(31,358)	5.5<br>(5.5)	^{6}I 78% + ^{4}H 12% + Other 10%<br>(^{6}I 79% + ^{4}H 12% + Other 9%)
32,319<br>(32,178)	6.5<br>(6.5)	^{6}I 82% + ^{4}H 6% + Other 12%<br>(^{6}I 82% + ^{4}H 6% + Other 12%)
32,506<br>(32,354)	7.5<br>(7.5)	^{6}I 84% + ^{4}K 10% + Other 5%<br>(^{6}I 85% + ^{4}K 10% + Other 5%)
33,640<br>(33,538)	4.5<br>(4.5)	^{6}I 11% + ^{6}D 58% + ^{6}F 12% + ^{4}F 11% + Other 8%<br>(^{6}I 10% + ^{6}D 59% + ^{6}F 12% + ^{4}F 11% + Other 8%)
36,440<br>(36,310)	3.5<br>(3.5)	^{6}P 12% + ^{6}D 42% + ^{6}F 12% + ^{4}D 18% + Other 15%<br>(^{6}P 13% + ^{6}D 43% + ^{6}F 12% + ^{4}D 17% + Other 15%)
37,840<br>(37,621)	0.5<br>(0.5)	^{6}D 93% + Other 7%<br>(^{6}D 93% + Other 7%)
38,532<br>(38,322)	2.5<br>(2.5)	^{6}P 32% + ^{6}D 39% + ^{6}F 11% + ^{4}D 10% + Other 8%<br>(^{6}P 32% + ^{6}D 40% + ^{6}F 10% + ^{4}D 9% + Other 8%)
38,626<br>(38,395)	1.5<br>(1.5)	^{6}P 22% + ^{6}D 63% + ^{6}F 6% + Other 10%<br>(^{6}P 21% + ^{6}D 63% + ^{6}F 6% + Other 10%)

Table S10. Energy and composition of the 15 lowest energy excited states of the free ion with spin orbit coupling for **Bk^{IV}**. Energy values are in cm^{-1}. The top text corresponds to LFDFT-PBE results and the text in parentheses to LFDFT-PBE0.

E (cm^{-1})	J	$^{2S+1}$L Composition
0<br>(0)	3.5<br>(3.5)	^{8}S 81% + ^{6}P 16% + Other 3%<br>(^{8}S 82% + ^{6}P 16% + Other 3%)
23,792<br>(23,978)	3.5<br>(3.5)	^{8}S 10% + ^{6}P 16% + ^{6}I 5% + ^{6}D 14% + ^{4}D 17% + ^{4}H 6% + ^{4}F 8% + ^{2}F 7% + Other 18%<br>(^{8}S 10% + ^{6}P 17% + ^{6}D 14% + ^{4}D 19% + ^{4}H 5% + ^{4}F 8% + ^{2}F 6% + Other 20%)
27,814<br>(27,860)	2.5<br>(2.5)	^{6}P 48% + ^{6}D 30% + ^{4}D 10% + Other 12%<br>(^{6}P 49% + ^{6}D 30% + ^{4}D 11% + Other 10%)
29,076<br>(29,133)	3.5<br>(3.5)	^{6}P 8% + ^{6}I 47% + ^{6}D 6% + ^{4}H 23% + Other 15%<br>(^{6}P 8% + ^{6}I 49% + ^{6}D 6% + ^{4}H 23% + Other 15%)
30,446<br>(30,428)	1.5<br>(1.5)	^{6}P 63% + ^{6}D 23% + ^{4}S 7% + Other 7%<br>(^{6}P 63% + ^{6}D 23% + ^{4}S 7% + Other 7%)
30,896<br>(30,954)	4.5<br>(4.5)	^{6}I 55% + ^{6}D 5% + ^{4}H 20% + Other 20%<br>(^{6}I 56% + ^{4}H 20% + Other 23%)
32,552<br>(32,551)	8.5<br>(8.5)	^{6}I 71% + ^{4}K 22% + Other 7%<br>(^{6}I 72% + ^{4}K 21% + Other 7%)
33,236<br>(33,212)	5.5<br>(5.5)	^{6}I 73% + ^{4}H 14% + Other 13%<br>(^{6}I 74% + ^{4}H 14% + Other 12%)
34,279<br>(34,229)	6.5<br>(6.5)	^{6}I 77% + ^{4}H 7% + ^{4}K 5% + Other 10%<br>(^{6}I 78% + ^{4}H 7% + ^{4}K 5% + Other 10%)
34,572<br>(34,504)	7.5<br>(7.5)	^{6}I 81% + ^{4}K 12% + Other 7%<br>(^{6}I 82% + ^{4}K 11% + Other 7%)
35,382<br>(35,370)	4.5<br>(4.5)	^{6}I 15% + ^{6}D 51% + ^{6}F 13% + Other 21%<br>(^{6}I 15% + ^{6}D 52% + ^{6}F 13% + ^{4}F 11% + Other 10%)
38,418<br>(38,402)	3.5<br>(3.5)	^{6}P 11% + ^{6}D 37% + ^{6}F 13% + ^{4}D 20% + ^{2}F 5% + Other 14%<br>(^{6}P 11% + ^{6}D 38% + ^{6}F 13% + ^{4}D 19% + ^{2}F 5% + Other 14%)
40,574<br>(40,435)	0.5<br>(0.5)	^{6}D 92% + Other 8%<br>(^{6}D 92% + Other 8%)
40,952<br>(40,923)	3.5<br>(3.5)	^{6}P 14% + ^{6}I 23% + ^{6}D 8% + ^{6}G 8% + ^{4}F 8% + ^{4}G 9% + ^{2}G 5% + ^{2}F 7% + Other 18%<br>(^{6}P 14% + ^{6}I 22% + ^{6}D 8% + ^{6}G 8% + ^{4}F 8% + ^{4}G 9% + ^{2}G 6% + ^{2}F 8% + Other 18%)
41,187<br>(41,077)	2.5<br>(2.5)	^{6}P 36% + ^{6}D 28% + ^{6}G 6% + ^{6}F 12% + ^{4}D 5% + Other 13%<br>(^{6}P 35% + ^{6}D 30% + ^{6}G 6% + ^{6}F 12% + ^{4}D 9% + Other 8%)

Slater-Condon parameters for f^7 complexes

Table S11. Slater-Condon parameters (cm^{-1}), F^2, F^4, and F^6 describing 4f-4f/5f-5f inter-electronic repulsion, and spin-orbit coupling constant (cm^{-1}), ζ for each of the **hexafluoride** f^7 complexes. Values after the vertical pipe represent the nephelauxetic reduction (%). The top text corresponds to LFDFT-PBE results, the text in parentheses to LFDFT-PBE0, the text in square brackets to CASSCF, and the text in curly braces to NEVPT2.

	F^2	F^4	F^6	ζ

**$[Gd^{III}F_6]^{3-}$**	99,588 | 11.1<br>(101,385 | 9.1)<br>[112,572 | 1.1]<br>{94,857 | 3.0}	62,104 | 11.3<br>(63,234 | 9.3)<br>[70,480 | 1.1]<br>{62,589 | 1.8}	44,569 | 11.4<br>(45,329 | 9.5)<br>[50,231 | 1.1]<br>{49,493 | 1.5}	1,494 | 6.4<br>(1,532 | 5.0)<br>[1,417 | 0.9]<br>{-}
**$[Tb^{IV}F_6]^{3-}$**	70,035 | 41.5<br>(71,686 | 39.9)<br>[120,092 | 0.9]<br>{100,799 | 5.7}	43,786 | 41.8<br>(44,829 | 40.2)<br>[75,478 | 0.9]<br>{66,894 | 3.4}	31,453 | 41.9<br>(32,206 | 40.4)<br>[53,570 | 0.9]<br>{52,515| 2.5}	1,401 | 25.7<br>(1,406 | 24.2)<br>[1,567 | 0.8]<br>{-}
**$[Cm^{III}F_6]^{3-}$**	72,351 | 12.4<br>(74,091 | 9.9)<br>[78,717 | 1.8]<br>{57,710 | 7.3}	46,979 | 12.7<br>(48,107 | 10.3)<br>[51,452 | 1.4]<br>{42,502 | 3.3}	34,401 | 12.8<br>(35,225 | 10.5)<br>[37,698 | 1.5]<br>{35,682 | 3.9}	2,904 | 7.5<br>(2,894 | 5.6)<br>[2,776 | 2.4]<br>{-}
**$[Bk^{IV}F_6]^{3-}$**	54,112 | 37.6<br>(57,007 | 34.3)<br>[85,265 | 2.3]<br>{59,049 | 14.8}	35,276 | 38.0<br>(37,174 | 34.6)<br>[56,172 | 1.9]<br>{44,678 | 8.7}	25,882 | 38.1<br>(27,278 | 34.7)<br>[41,308 | 1.9]<br>{37,531 | 8.3}	2,799 | 23.5<br>(2,837 | 21.8)<br>[3,303 | 2.9]<br>{-}

Table S12. Slater-Condon parameters (cm^{-1}), F^2, F^4, and F^6 describing 4f-4f/5f-5f inter-electronic repulsion, and spin-orbit coupling constant (cm^{-1}), ζ for each of the **hexachloride** f^7 complexes. Values after the vertical pipe represent the nephelauxetic reduction (%). The top text corresponds to LFDFT-PBE results, the text in parentheses to LFDFT-PBE0, the text in square brackets to CASSCF, and the text in curly braces to NEVPT2.

	**F^2**	**F^4**	**F^6**	**ζ**
**$[Gd^{III}Cl_6]^{3-}$**	90,578 | 19.2<br>(91,059 | 18.4)<br>[112,575 | 1.1]<br>{94,613 | 3.3}	56,452 | 19.4<br>(56,756 | 18.6)<br>[70,485 | 1.1]<br>{62,300 | 2.3}	40,503 | 19.5<br>(40,722 | 18.7)<br>[50,223 | 1.1]<br>{49,389 | 1.7}	1,419 | 11.1<br>(1,407 | 12.8)<br>[1,418 | 0.8]<br>{-}
**$[Tb^{IV}Cl_6]^{3-}$**	55,690 | 53.5<br>(54,592 | 54.3)<br>[120,147 | 0.9]<br>{100,557 | 5.9}	34,768 | 53.8<br>(34,081 | 54.6)<br>[75,496 | 0.9]<br>{66,756 | 3.6}	24,962 | 53.9<br>(24,469 | 54.7)<br>[53,568 | 0.9]<br>{52,641 | 2.3}	1,236 | 34.5<br>(1,212 | 34.7)<br>[1,568 | 0.7]<br>{-}
**$[Cm^{III}Cl_6]^{3-}$**	66,465 | 19.5<br>(67,535 | 17.9)<br>[78,515 | 2.0]<br>{57,410 | 7.8}	43,104 | 19.9<br>(43,796 | 18.3)<br>[51,197 | 1.9]<br>{42,146 | 4.1}	31,548 | 20.1<br>(32,052 | 18.6)<br>[37,460 | 2.1]<br>{35,800 | 3.6}	2,773 | 11.7<br>(2,752 | 10.2)<br>[2,784 | 2.1]<br>{-}
**$[Bk^{IV}Cl_6]^{3-}$**	41,935 | 51.6<br>(42,686 | 50.8)<br>[85,289 | 2.3]<br>{56,699 | 18.2}	27,271 | 52.0<br>(27,761 | 51.2)<br>[56,015 | 2.1]<br>{43,630 | 10.8}	19,987 | 52.2<br>(20,346 | 51.3)<br>[41,120 | 2.3]<br>{37,600 | 8.1}	2,434 | 33.5<br>(2,421 | 33.3)<br>[3,318 | 2.4]<br>{-}

Table S13. Slater-Condon parameters (cm^{-1}), F^2, F^4, and F^6 describing 4f-4f/5f-5f inter-electronic repulsion, and spin-orbit coupling constant (cm^{-1}), ζ for each of the **hexabromide** f^7 complexes. Values after the vertical pipe represent the nephelauxetic reduction (%). The top text corresponds to

LFDFT-PBE results, the text in parentheses to LFDFT-PBE0, the text in square brackets to CASSCF, and the text in curly braces to NEVPT2.

	F^2	F^4	F^6	ζ
**$[Gd^{III}Br_6]^{3-}$**	86,969 | 22.4<br>(86,891 | 22.1)<br>[112,615 | 1.0]<br>{94,900 | 3.0}	54,188 | 22.6<br>(54,141 | 22.3)<br>[70,510 | 1.1]<br>{62,564 | 1.9}	38,875 | 22.7<br>(38,842 | 22.5)<br>[50,240 | 1.1]<br>{49,553 | 1.4}	1,387 | 13.0<br>(1,372 | 14.9)<br>[1,418 | 0.8]<br>{-}
**$[Tb^{IV}Br_6]^{3-}$**	51,961 | 56.6<br>(50,026 | 58.1)<br>[120,185 | 0.8]<br>{99,879 | 6.6}	32,424 | 56.9<br>(31,211 | 58.4)<br>[75,517 | 0.9]<br>{66,415 | 4.1}	23,274 | 57.0<br>(22,402 | 58.5)<br>[53,582 | 0.9]<br>{52,531 | 2.5}	1,190 | 36.9<br>(1,155 | 37.7)<br>[1,568 | 0.7]<br>{-}
**$[Cm^{III}Br_6]^{3-}$**	63,534 | 23.0<br>(64,322 | 21.8)<br>[78,586 | 2.0]<br>{57,424 | 7.8}	41,200 | 23.4<br>(41,708 | 22.2)<br>[51,215 | 1.9]<br>{42,119 | 4.2}	30,152 | 23.6<br>(30,522 | 22.5)<br>[37,470 | 2.1]<br>{35,842 | 3.5}	2,707 | 13.8<br>(2,682 | 12.5)<br>[2,787 | 2.0]<br>{-}
**$[Bk^{IV}Br_6]^{3-}$**	38,220 | 55.9<br>(38,407 | 55.7)<br>[85,390 | 2.2]<br>{56,639 | 18.3}	24,843 | 56.3<br>(24,964 | 56.1)<br>[56,039 | 2.1]<br>{44,410 | 9.3}	18,203 | 56.5<br>(18,291 | 56.2)<br>[41,133 | 2.3]<br>{38,210 | 6.7}	2,314 | 36.8<br>(2,287 | 37.0)<br>[3,322 | 2.3]<br>{-}

Table S14. Slater-Condon parameters (cm^{-1}), F^2, F^4, and F^6 describing 4f-4f/5f-5f inter-electronic repulsion, and spin-orbit coupling constant (cm^{-1}), ζ for each of the **nona-aquo** f^7 complexes. Values after the vertical pipe represent the nephelauxetic reduction (%). The top text corresponds to LFDFT-PBE results and the text in parentheses to LFDFT-PBE0.

	F^2	F^4	F^6	ζ
**$[Gd^{III}(H_2O_9)]^{3+}$**	100,059 | 10.7<br>(102,540 | 8.1)	62,404 | 10.9<br>(63,965 | 8.2)	44,785 | 11.0<br>(45,909 | 8.3)	1,498 | 6.1<br>(1,502 | 6.9)
**$[Tb^{IV}(H_2O_9)]^{4+}$**	63,289 | 47.1<br>(-)	39,538 | 47.4<br>(-)	28,394 | 47.5<br>(-)	1,324 | 29.8<br>(-)
**$[Cm^{III}(H_2O_9)]^{3+}$**	73,611 | 10.8<br>(75,151 | 8.7)	47,806 | 11.2<br>(48,803 | 9.0)	35,010 | 11.3<br>(35,736 | 9.2)	2,935 | 6.5<br>(2,920 | 4.7)
**$[Bk^{IV}(H_2O_9)]^{4+}$**	51,997 | 40.0<br>(55,565 | 35.9)	33,895 | 40.4<br>(36,236 | 36.3)	24,868 | 40.6<br>(26,589 | 36.4)	2,741 | 25.1<br>(2,800 | 22.8)

Table S15. Slater-Condon parameters (cm^{-1}), F^2, F^4, and F^6 describing 4f-4f/5f-5f inter-electronic repulsion, and spin-orbit coupling constant (cm^{-1}), ζ for each of the **tetra-arsine oxide** f^7 complexes.

Values after the vertical pipe represent the nephelauxetic reduction (%). The top text corresponds to LFDFT-PBE results and the text in parentheses to LFDFT-PBE0.

	F^2	F^4	F^6	ζ
**$[Gd^{III}(AsOPh_3)_4]^+$**	95,088 | 15.1<br>(97,438 | 12.6)	59,281 | 15.3<br>(60,755 | 12.8)	42,537 | 15.4<br>(43,598 | 13.0)	1,457 | 8.6<br>(1,462 | 9.4)
**$[Tb^{IV}(AsOPh_3)_4]^{2+}$**	59,513 | 50.3<br>(-)	37,165 | 50.6<br>(-)	26,686 | 50.7<br>(-)	1,282 | 32.1<br>(-)
**$[Cm^{III}(AsOPh_3)_4]^+$**	66,174 | 19.8<br>(69,125 | 16.0)	42,951 | 20.2<br>(44,860 | 16.4)	31,446 | 20.3<br>(32,841 | 16.6)	2,779 | 11.5<br>(2,795 | 8.8)
**$[Bk^{IV}(AsOPh_3)_4]^{2+}$**	45,220 | 47.9<br>(48,232 | 44.4)	29,444 | 48.2<br>(31,415 | 44.8)	21,592 | 48.4<br>(23,039 | 44.9)	2,541 | 30.6<br>(2,592 | 28.6)

QTAIM data

Table S16. Calculated average electron density (ρ), delocalization index (DI), kinetic energy (G), potential energy (V), total energy (H) and Laplacian ($\nabla^2\rho$) at the bond critical point. Optimized bond lengths are from the level of theory specified in the methods section of the main text. For QTAIM results, PBE results are shown on top, with PBE0 results below in parentheses.

		Gd^{III}	Tb^{IV}	Cm^{III}	Bk^{IV}
**$[M(H_2O)_9]^q$<br>M–O**	Length (Å)	2.476	2.495	2.354	2.323
	ρ(r)<br>(|e| $Å^{-3}$)	0.2708<br>(0.2632)	0.3637<br>(-)	0.3025<br>(0.2969)	0.4515<br>(0.4521)
	$\nabla^2\rho$	0.1680<br>(0.1715)	0.2217<br>(-)	0.1923<br>(0.1967)	0.3002<br>(0.2861)
	G(r)<br>(kcal $mol^{-1}Å^{-3}$)	175.8<br>(176.6)	250.2<br>(-)	204.5<br>(159.5)	346.4<br>(335.6)
	V(r)<br>(kcal $mol^{-1}Å^{-3}$)	-173.7<br>(-171.6)	-265.7<br>(-)	-205.5<br>(-156.9)	-375.0<br>(-368.3)
	H(r)<br>(kcal $mol^{-1}Å^{-3}$)	2.0<br>(5.0)	-15.5<br>(-)	-1.0<br>(2.6)	-28.6<br>(-32.7)
	DI	0.2241<br>(0.2020)	0.3898<br>(-)	0.2582<br>(0.2340)	0.1273<br>(0.3370)
**$[MF_6]^q$<br>M–F**	Length (Å)	2.289	2.344	2.094	2.148
	ρ(r)<br>(|e| $Å^{-3}$)	0.3400<br>(0.3307)	0.3610<br>(0.3644)	0.4108<br>(0.4049)	0.4245<br>(0.4251)
	$\nabla^2\rho$	0.2344<br>(0.2409)	0.2186<br>(0.2140)	0.2691<br>(0.2120)	0.2617<br>(0.2616)
	G(r)<br>(kcal $mol^{-1}Å^{-3}$)	249.0<br>(250.1)	246.7<br>(245.3)	304.5<br>(285.2)	305.6<br>(305.5)

	V(r) (kcal mol^{-1}Å^{-3})	-249.9 (-245.2)	-261.9 (-264.1)	-324.1 (-345.9)	-334.3 (-334.0)
	H(r) (kcal mol^{-1}Å^{-3})	-0.8 (4.9)	-15.2 (-18.8)	-19.6 (-9.5)	-28.6 (-28.5)
	DI	0.2879 (0.2470)	0.5709 (0.4770)	0.4746 (0.3800)	0.5833 (0.4810)
**$[MCl_6]^q$ M–Cl**	Length (Å)	2.758	2.796	2.563	2.592
	ρ(r) (|e| Å^{-3})	0.2733 (0.2699)	0.4054 (0.4116)	0.2910 (0.2902)	0.4430 (0.4454)
	$\nabla^2\rho$	0.1115 (0.1138)	0.1609 (0.1548)	0.1207 (0.1238)	0.1726 (0.1704)
	G(r) (kcal mol^{-1}Å^{-3})	136.8 (137.4)	225.6 (225.8)	149.6 (150.3)	251.7 (252.4)
	V(r) (kcal mol^{-1}Å^{-3})	-155.5 (-154.2)	-280.8 (-287.6)	-171.5 (-169.6)	-320.7 (-324.4)
	H(r) (kcal mol^{-1}Å^{-3})	-18.7 (-16.8)	-55.3 (-61.9)	-21.9 (-19.3)	-69.0 (-72.0)
	DI	0.4350 (0.3890)	0.7145 (0.6390)	0.4773 (0.4260)	0.7539 (0.6720)
**$[MBr_6]^q$ M–Br**	Length (Å)	2.910	2.947	2.712	2.741
	ρ(r) (|e| Å^{-3})	0.2439 (0.2429)	0.3564 (0.3644)	0.2582 (0.2564)	0.3844 (0.3914)
	$\nabla^2\rho$	0.0819 (0.0841)	0.1145 (0.1082)	0.0877 (0.0904)	0.1228 (0.1191)
	G(r) (kcal mol^{-1}Å^{-3})	105.8 (106.8)	171.2 (171.6)	114.7 (115.8)	189.2 (190.0)
	V(r) (kcal mol^{-1}Å^{-3})	-125.0 (-124.5)	-221.2 (-228.6)	-136.6 (-135.9)	-248.4 (-253.8)
	H(r) (kcal mol^{-1}Å^{-3})	-19.2 (-17.7)	-50.0 (-57.0)	-21.9 (-20.1)	-59.2 (-63.9)
	DI	0.4632 (0.4170)	0.7639 (0.6930)	0.5039 (0.4570)	0.7911 (0.7240)
**$[M(AsOPh_3)_4(NO_3)_2]^q$ M–O**	Length (Å)	2.285	2.328	2.172	2.207
	ρ(r) (|e| Å^{-3})	0.4265 (0.4218)	0.5520 (-)	0.4517 (0.4461)	0.5960 (0.5982)
	$\nabla^2\rho$	0.2737 (0.2790)	0.3532 (-)	0.2882 (0.2953)	0.3767 (0.3767)
	G(r) (kcal mol^{-1}Å^{-3})	315.2 (316.7)	436.7 (-)	337.6 (339.9)	478.9 (480.2)
	V(r) (kcal mol^{-1}Å^{-3})	-340.6 (-338.0)	-499.5 (-)	-370.1 (-367.1)	-558.9 (-561.6)
	H(r) (kcal mol^{-1}Å^{-3})	-25.4 (-21.3)	-62.8 (-)	-32.5 (-27.3)	-80.1 (-81.4)
	DI	0.3622 (0.3303)	0.5675 (-)	0.4061 (0.3705)	0.6072 (0.5437)

NLMO Data

Below is the raw input file of a NBO calculation in ADF of $[Tb^{IV}Br_6]^{3-}$.

```
"$AMSBIN/ams" << eor

Task SinglePoint

System

  Atoms

    Tb 0.0 0.0 0.0 adf.R=2.250

    Br 1e-06 2.712317 2e-06 adf.R=1.850

    Br -2e-06 -2.712318 -2e-06 adf.R=1.850

    Br 2.712319 -1e-06 0.0 adf.R=1.850

    Br -2.712317 -5e-06 2e-06 adf.R=1.850

    Br -1e-06 6e-06 2.712317 adf.R=1.850

    Br 1e-06 2e-06 -2.712318 adf.R=1.850

  End

  Charge -2

  BondOrders

     1 2 1.0

     1 3 1.0

     1 4 1.0

     1 5 1.0

     1 6 1.0

     1 7 1.0

  End

End

Engine ADF

  Basis

    Type TZP
```

```
    Core None
  End
  SpinPolarization 7
  Save TAPE15
  XC
    GGA PBE
    DISPERSION GRIMME3 BJDAMP
  End
  Unrestricted Yes
  Solvation
    Surf Delley
    Solv name=Water cav0=0.0 cav1=0.0067639
    Charged method=CONJ
    C-Mat POT
    SCF VAR ALL
    CSMRSP
  End
  FullFock Yes
  AOMat2File Yes
EndEngine
eor
# ============
# NBO Analysis
# ============
cp "${AMS_RESULTSDIR-${AMS_JOBNAME-ams}.results}/adf.rkf" TAPE21
cp "${AMS_RESULTSDIR-${AMS_JOBNAME-ams}.results}/TAPE15" TAPE15
"$AMSBIN/adfnbo" << eor
write
```

```
spherical
eor
"$AMSBIN/gennbo6" FILE47
"$AMSBIN/adfnbo" << eor
spherical
fock
read
eor
mv TAPE21 "${AMS_RESULTSDIR-${AMS_JOBNAME-ams}.results}/adf.rkf"
rm -f TAPE15
```

Table S17. Average population of the metal 5d/4f (Gd^{III}, Tb^{IV}) or 6d/5f (Cm^{III}, Bk^{IV}) orbital within bonding and ligand NLMOs using the PBE functional. Values are averaged over all ligands.

		**Gd^{III}**		**Tb^{IV}**		**Cm^{III}**		**Bk^{IV}**	
		d	f	d	f	d	f	d	f
**$[M(H_2O)_9]^q$**	α	0.017	0.000	0.029	0.000	0.019	0.000	0.034	0.000
	β	0.019	0.003	0.030	0.040	0.021	0.007	0.032	0.039
**$[MF_6]^q$**	α	0.016	0.000	0.024	0.000	0.014	0.000	0.021	0.000
	β	0.014	0.003	0.024	0.028	0.013	0.007	0.020	0.030
**$[MCl_6]^q$**	α	0.025	0.000	0.036	0.000	0.026	0.000	0.037	0.000
	β	0.023	0.004	0.035	0.037	0.023	0.008	0.036	0.042
**$[MBr_6]^q$**	α	0.028	0.000	0.038	0.000	0.029	0.000	0.040	0.000
	β	0.025	0.004	0.038	0.040	0.025	0.008	0.039	0.045
**$[M(AsOPh_3)_4(NO_3)]^q$**	α	0.008	0.000	0.012	0.000	0.005	0.000	0.012	0.000
	β	0.008	0.003	0.012	0.025	0.005	0.006	0.011	0.028

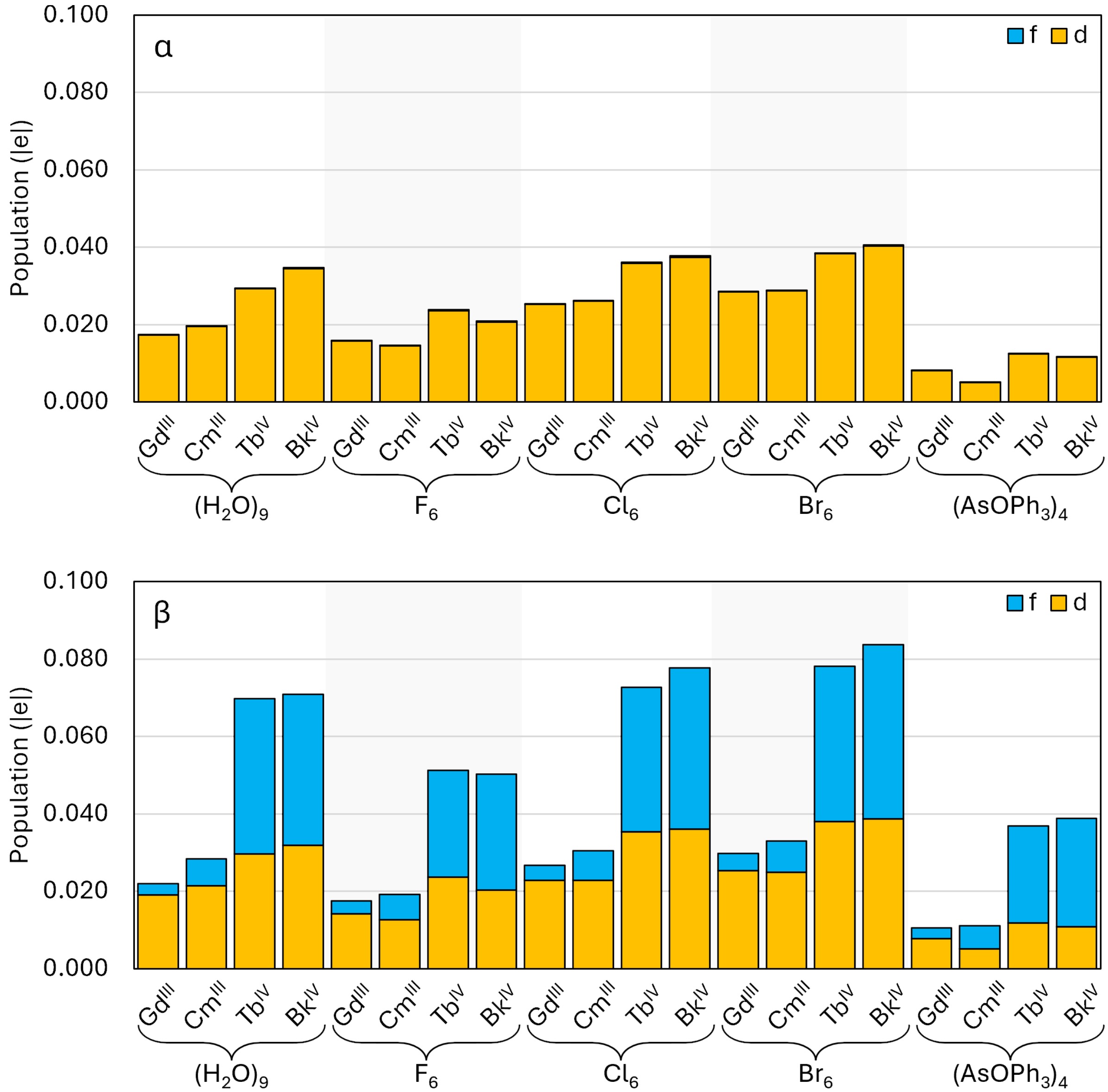

Figure S4. Graphical representation of the average population of the metal 5d/4f (Gd^{III}, Tb^{IV}) or 6d/5f (Cm^{III}, Bk^{IV}) orbital within bonding and ligand NLMOs using the PBE functional. Values are averaged over all ligands.

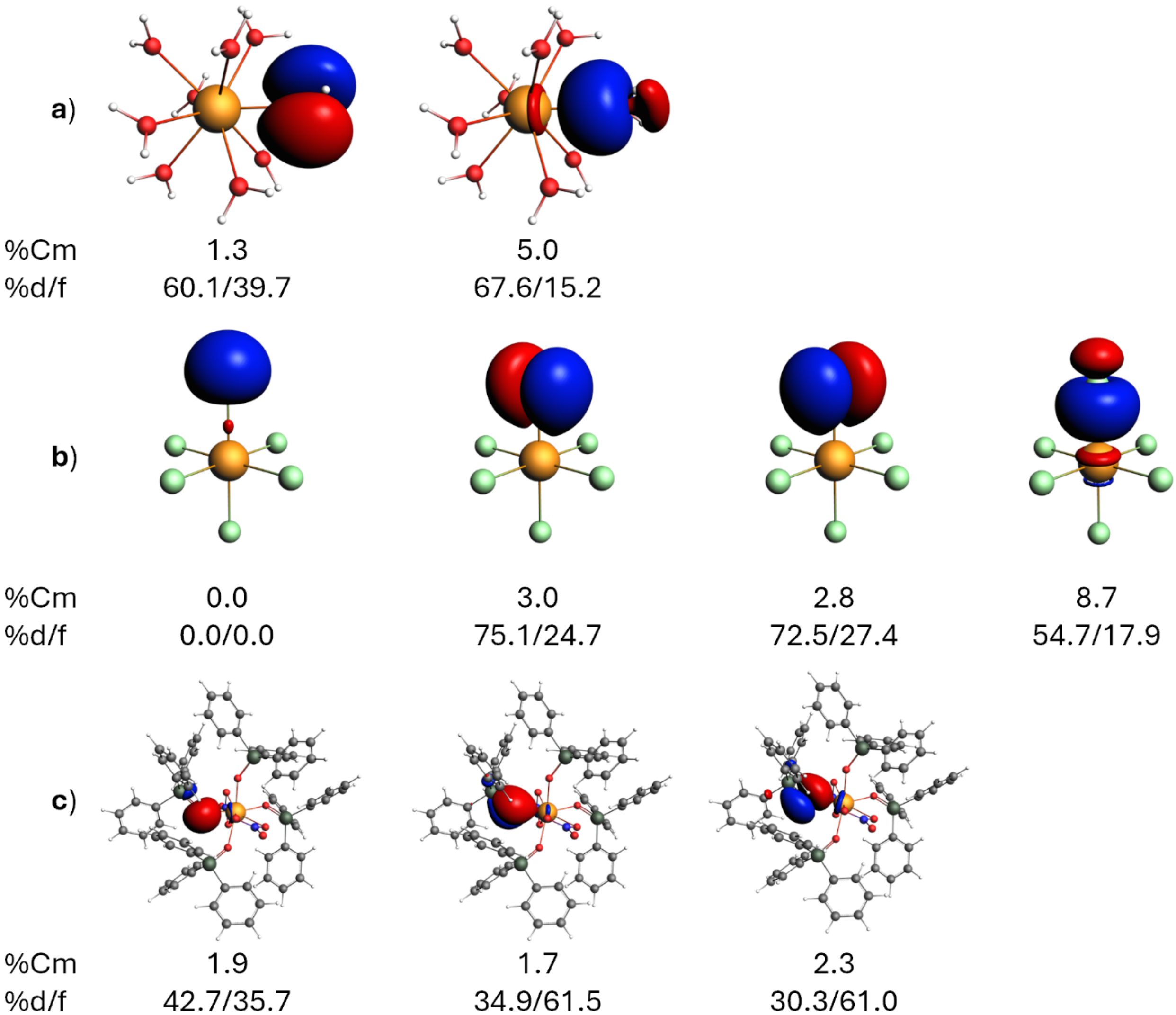

Figure S5. Selected beta NLMOs using the PBE functional in **a**) $[Cm^{III}(H_2O)_9]^{3+}$, **b**) $[Cm^{III}Cl_6]^{3-}$, and **c**) $[Cm^{III}(OAsPh_3)_4(NO_3)_2]^{+}$. Below the figures are the total metal contributions and individual 6d and 5f orbital contributions to the hybrid. For simplicity, orbitals from only one ligand are shown. Orbitals from other hexahalides are qualitatively similar to those in the hexachloride.

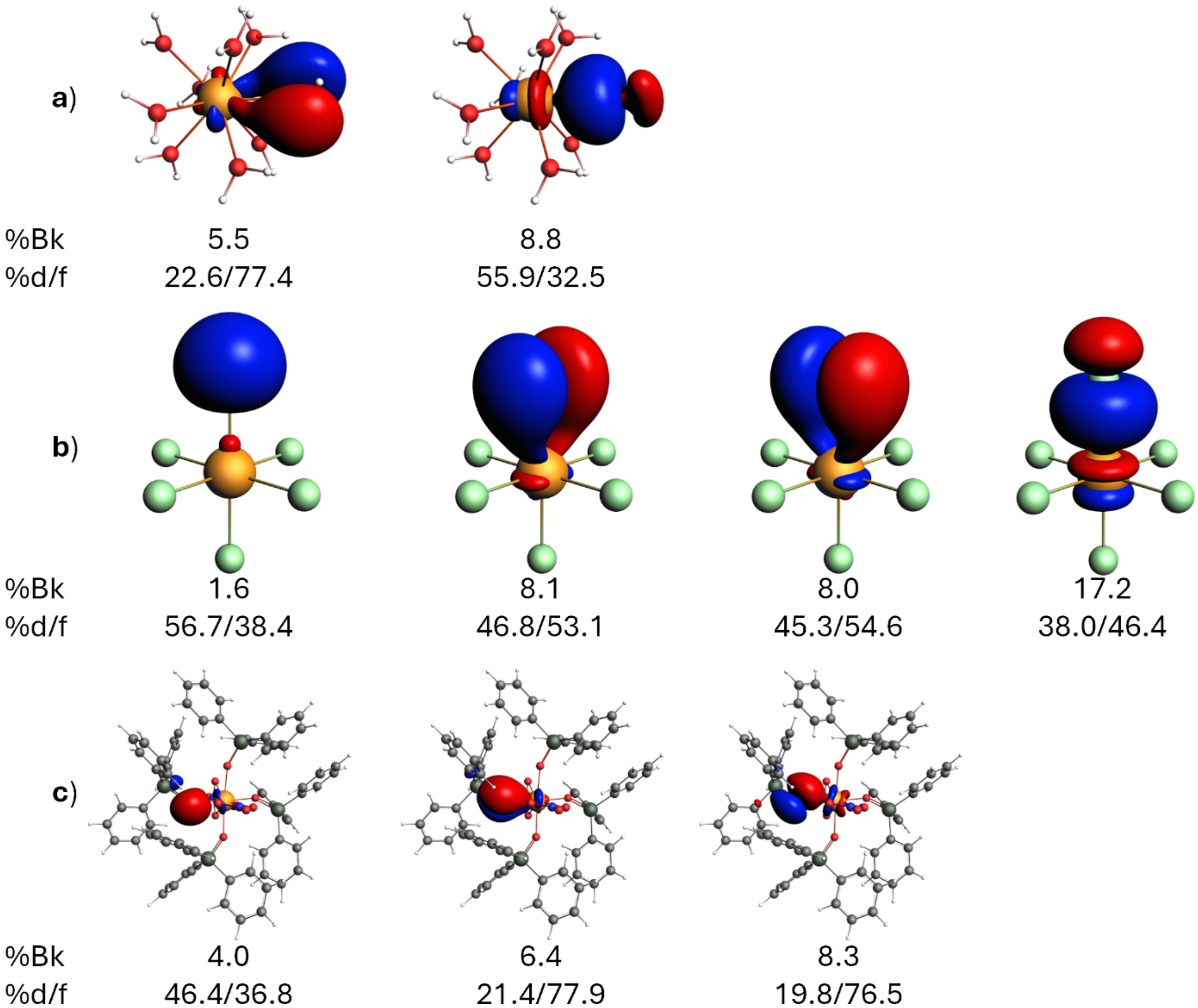

Figure S6. Selected beta NLMOs using the PBE functional in **a**) $[Bk^{IV}(H_2O)_9]^{4+}$, **b**) $[Bk^{IV}Cl_6]^{2-}$, and **c**) $[Bk^{IV}(OAsPh_3)_4(NO_3)_2]^{2+}$. Below the figures are the total metal contributions and individual 6d and 5f orbital contributions to the hybrid. For simplicity, orbitals from only one ligand are shown. Orbitals from other hexahalides are qualitatively similar to those in the hexachloride.

Energy and composition of the low-lying excited states for f^7 complexes

Table S18. Energy (cm^{-1}) and composition of the excited states less than **42,000 cm^{-1}** for **$[Gd^{III}(AsOPh_3)_4(NO_3)_2]^+$**. The top text corresponds to LFDFT-PBE results and the text in parentheses to LFDFT-PBE0.

E (cm^{-1})	J	$^{2S+1}$L Composition
1<br>(1)	3.5<br>(3.5)	^{8}S 97% + Other 3%<br>(^{8}S 97% + Other 3%)
31,167<br>(32,045)	3.5<br>(3.5)	^{6}P 75% + ^{6}D 12% + ^{4}D 8% + Other 5%<br>(^{6}P 77% + ^{6}D 12% + ^{4}D 7% + Other 4%)

31,224<br>(32,078)	3.5<br>(3.5)	^{6}P 76% + ^{6}D 12% + ^{4}D 8% + Other 5%<br>(^{6}P 77% + ^{6}D 12% + ^{4}D 7% + Other 4%)
31,269<br>(32,109)	3.5<br>(3.5)	^{6}P 76% + ^{6}D 12% + ^{4}D 8% + Other 5%<br>(^{6}P 77% + ^{6}D 11% + ^{4}D 7% + Other 4%)
31,295<br>(32,158)	3.5<br>(3.5)	^{6}P 76% + ^{6}D 11% + ^{4}D 8% + Other 5%<br>(^{6}P 77% + ^{6}D 11% + ^{4}D 7% + Other 4%)
31,774<br>(32,637)	2.6<br>(2.5)	^{6}P 83% + ^{6}D 13% + Other 4%<br>(^{6}P 84% + ^{6}D 12% + Other 3%)
31,810<br>(32,656)	2.6<br>(2.5)	^{6}P 83% + ^{6}D 12% + Other 4%<br>(^{6}P 84% + ^{6}D 12% + Other 3%)
31,844<br>(32,692)	2.5<br>(2.5)	^{6}P 84% + ^{6}D 12% + Other 4%<br>(^{6}P 85% + ^{6}D 12% + Other 3%)
32,335<br>(33,185)	1.6<br>(1.5)	^{6}P 89% + ^{6}D 7% + Other 3%<br>(^{6}P 90% + ^{6}D 7% + Other 2%)
32,379<br>(33,220)	1.6<br>(1.5)	^{6}P 90% + ^{6}D 7% + Other 3%<br>(^{6}P 91% + ^{6}D 7% + Other 2%)
34,265<br>(35,174)	3.5<br>(3.5)	^{6}I 92% + ^{4}H 7% + Other 1%<br>(^{6}I 92% + ^{4}H 7% + Other 1%)
34,301<br>(35,194)	3.5<br>(3.5)	^{6}I 92% + ^{4}H 7% + Other 1%<br>(^{6}I 93% + ^{4}H 7% + Other 1%)
34,330<br>(35,206)	3.5<br>(3.5)	^{6}I 92% + ^{4}H 7% + Other 1%<br>(^{6}I 93% + ^{4}H 7% + Other 1%)
34,353<br>(35,228)	3.5<br>(3.5)	^{6}I 92% + ^{4}H 7% + Other 1%<br>(^{6}I 93% + ^{4}H 7% + Other 1%)
34,633<br>(35,537)	4.5<br>(4.5)	^{6}I 94% + Other 6%<br>(^{6}I 94% + Other 6%)
34,657<br>(35,547)	4.5<br>(4.5)	^{6}I 94% + Other 6%<br>(^{6}I 94% + Other 6%)
34,688<br>(35,569)	4.5<br>(4.5)	^{6}I 94% + Other 6%<br>(^{6}I 95% + Other 5%)
34,718<br>(35,586)	4.5<br>(4.5)	^{6}I 94% + Other 6%<br>(^{6}I 95% + Other 5%)
34,733<br>(35,596)	4.5<br>(4.5)	^{6}I 94% + Other 6%<br>(^{6}I 95% + Other 5%)
34,880<br>(35,785)	8.3<br>(8.5)	^{6}I 96% + Other 4%<br>(^{6}I 96% + Other 4%)
34,888<br>(35,839)	8.4<br>(5.6)	^{6}I 96% + Other 4%<br>(^{6}I 96% + Other 4%)
34,896<br>(35,846)	8.4<br>(5.6)	^{6}I 96% + Other 4%<br>(^{6}I 96% + Other 4%)
34,908<br>(35,864)	8.5<br>(5.5)	^{6}I 96% + Other 4%<br>(^{6}I 96% + Other 4%)
34,923<br>(35,883)	8.3<br>(5.5)	^{6}I 95% + Other 5%<br>(^{6}I 96% + Other 4%)
34,944<br>(35,892)	5.9<br>(5.5)	^{6}I 96% + Other 4%<br>(^{6}I 96% + Other 4%)

34,962<br>(35,902)	5.8<br>(5.5)	^{6}I 96% + Other 4%<br>(^{6}I 96% + Other 4%)
34,983<br>(36,020)	5.6<br>(6.7)	^{6}I 96% + Other 4%<br>(^{6}I 97% + Other 3%)
35,012<br>(36,028)	5.6<br>(6.7)	^{6}I 96% + Other 4%<br>(^{6}I 97% + Other 3%)
35,037<br>(36,035)	5.5<br>(7.0)	^{6}I 96% + Other 4%<br>(^{6}I 97% + Other 3%)
35,044<br>(36,046)	5.5<br>(7.4)	^{6}I 96% + Other 4%<br>(^{6}I 97% + Other 3%)
35,128<br>(36,057)	6.6<br>(7.2)	^{6}I 97% + Other 3%<br>(^{6}I 97% + Other 3%)
35,143<br>(36,063)	6.8<br>(6.9)	^{6}I 97% + Other 3%<br>(^{6}I 97% + Other 3%)
35,153<br>(36,067)	7.3<br>(7.2)	^{6}I 97% + Other 3%<br>(^{6}I 97% + Other 3%)
35,161<br>(36,077)	6.9<br>(7.3)	^{6}I 97% + Other 3%<br>(^{6}I 97% + Other 3%)
35,165<br>(36,094)	7.2<br>(7.5)	^{6}I 97% + Other 3%<br>(^{6}I 97% + Other 3%)
35,176<br>(39,853)	7.3<br>(4.5)	^{6}I 97% + Other 3%<br>(^{6}D 92% + Other 8%)
35,188<br>(39,893)	6.9<br>(4.5)	^{6}I 97% + Other 3%<br>(^{6}D 93% + Other 7%)
35,199<br>(39,958)	7.1<br>(4.5)	^{6}I 97% + Other 3%<br>(^{6}D 93% + Other 7%)
35,207<br>(39,979)	7.3<br>(4.5)	^{6}I 97% + Other 3%<br>(^{6}D 93% + Other 7%)
35,215<br>(40,002)	6.7<br>(4.5)	^{6}I 97% + Other 3%<br>(^{6}D 93% + Other 7%)
35,223<br>(40,746)	7.2<br>(0.9)	^{6}I 97% + Other 3%<br>(^{6}D 98% + Other 2%)
35,227<br>(40,856)	7.0<br>(3.2)	^{6}I 97% + Other 3%<br>(^{6}P 10% + ^{6}D 83% + Other 7%)
35,241<br>(40,868)	7.3<br>(3.4)	^{6}I 97% + Other 3%<br>(^{6}P 11% + ^{6}D 82% + Other 7%)
35,250<br>(40,880)	7.2<br>(3.4)	^{6}I 97% + Other 3%<br>(^{6}P 11% + ^{6}D 82% + Other 7%)
38,815<br>(40,899)	4.5<br>(3.5)	^{6}D 92% + Other 8%<br>(^{6}P 11% + ^{6}D 81% + Other 8%)
38,876<br>(40,984)	4.5<br>(1.9)	^{6}D 92% + Other 8%<br>(^{6}P 7% + ^{6}D 89% + Other 3%)
38,933<br>(41,032)	4.5<br>(1.8)	^{6}D 92% + Other 8%<br>(^{6}P 8% + ^{6}D 89% + Other 3%)
39,017<br>(41,108)	4.5<br>(2.5)	^{6}D 93% + Other 7%<br>(^{6}P 12% + ^{6}D 83% + Other 5%)

39,038<br>(41,153)	4.5<br>(2.5)	^{6}D 93% + Other 7%<br>(^{6}P 12% + ^{6}D 83% + Other 5%)
39,752<br>(41,188)	1.3<br>(2.5)	^{6}D 96% + Other 4%<br>(^{6}P 12% + ^{6}D 83% + Other 5%)
39,853	3.1	^{6}P 10% + ^{6}D 83% + Other 7%
39,878	3.3	^{6}P 11% + ^{6}D 81% + Other 8%
39,888	3.3	^{6}P 10% + ^{6}D 82% + Other 7%
39,923	3.4	^{6}P 11% + ^{6}D 81% + Other 8%
40,013	2.2	^{6}P 8% + ^{6}D 88% + Other 4%
40,074	2.0	^{6}P 8% + ^{6}D 88% + Other 4%
40,109	2.5	^{6}P 12% + ^{6}D 82% + Other 5%
40,193	2.5	^{6}P 12% + ^{6}D 83% + Other 5%
40,253	2.5	^{6}P 12% + ^{6}D 83% + Other 5%

Table S19. Energy (cm^{-1}) and composition of the excited states less than **25,400 cm^{-1}** for **$[Tb^{IV}(AsOPh_3)_4(NO_3)_2]^{2+}$**. The text corresponds to LFDFT-PBE results.

E (cm^{-1})	J	$^{2S+1}$L Composition
5	3.5	^{8}S 94% + ^{6}P 5% + Other 0%
18,527	3.6	^{6}P 57% + ^{6}D 17% + ^{4}D 12% + Other 14%
18,691	3.6	^{6}P 59% + ^{6}D 16% + ^{4}D 12% + Other 12%
18,760	3.6	^{6}P 59% + ^{6}D 16% + ^{4}D 12% + Other 13%
18,888	3.6	^{6}P 61% + ^{6}D 15% + ^{4}D 12% + Other 12%
19,276	2.8	^{6}P 68% + ^{6}I 5% + ^{6}D 18% + Other 8%
19,376	2.8	^{6}P 71% + ^{6}D 18% + Other 12%
19,495	2.7	^{6}P 73% + ^{6}D 17% + Other 10%
19,966	2.1	^{6}P 78% + ^{6}I 5% + ^{6}D 11% + Other 5%
20,041	2.1	^{6}P 79% + ^{6}I 6% + ^{6}D 10% + Other 5%
20,858	3.6	^{6}I 80% + ^{4}H 12% + Other 8%
20,982	3.6	^{6}I 82% + ^{4}H 12% + Other 5%
21,051	3.6	^{6}I 83% + ^{4}H 12% + Other 5%
21,129	3.6	^{6}I 83% + ^{4}H 12% + Other 4%

21,344	4.6	^{6}I 85% + ^{4}H 9% + Other 6%
21,413	4.6	^{6}I 85% + ^{4}H 9% + Other 6%
21,524	4.8	^{6}I 88% + ^{4}H 8% + Other 4%
21,554	4.7	^{6}I 88% + ^{4}H 8% + Other 4%
21,591	5.3	^{6}I 89% + ^{4}H 7% + Other 4%
21,637	7.5	^{6}I 89% + ^{4}K 5% + Other 6%
21,665	7.6	^{6}I 90% + ^{4}K 5% + Other 5
21,679	8.0	^{6}I 90% + ^{4}K 6% + Other 4%
21,719	8.0	^{6}I 90% + ^{4}K 6% + Other 4%
21,736	7.7	^{6}I 90% + ^{4}K 6% + Other 4%
21,754	7.7	^{6}I 90% + ^{4}K 6% + Other 5%
21,768	7.8	^{6}I 90% + ^{4}K 6% + Other 4%
21,786	8.0	^{6}I 90% + ^{4}K 6% + Other 3%
21,801	7.9	^{6}I 90% + ^{4}K 6% + Other 4%
21,822	7.4	^{6}I 90% + Other 10%
21,849	7.5	^{6}I 89% + ^{4}K 5% + Other 6%
21,866	6.9	^{6}I 90% + Other 10%
21,925	6.0	^{6}I 92% + Other 8%
21,941	6.1	^{6}I 91% + Other 9%
21,961	6.3	^{6}I 91% + Other 9%
21,984	6.2	^{6}I 92% + Other 8%
22,018	7.1	^{6}I 92% + Other 8%
22,040	7.0	^{6}I 92% + Other 8%
22,055	6.7	^{6}I 92% + Other 8%
22,091	7.1	^{6}I 93% + Other 7%
22,116	6.9	^{6}I 93% + Other 7%
22,128	6.9	^{6}I 93% + Other 7%
22,142	7.0	^{6}I 93% + Other 7%
22,160	7.0	^{6}I 93% + Other 7%

22,178	6.9	^{6}I 93% + Other 7%
22,206	6.9	^{6}I 94% + Other 6%
22,221	7.0	^{6}I 94% + Other 6%
22,235	7.0	^{6}I 93% + Other 7%
22,278	6.9	^{6}I 91% + Other 9%
22,306	7.1	^{6}I 91% + Other 9%
23,845	4.6	^{6}D 79% + ^{6}F 8% + ^{4}F 6% + Other 7%
23,972	4.5	^{6}D 81% + ^{6}F 7% + ^{4}F 6% + Other 5%
24,097	4.5	^{6}D 83% + ^{6}F 6% + ^{4}F 6% + Other 5%
24,196	4.5	^{6}D 82% + ^{4}F 6% + Other 11%
24,329	4.5	^{6}D 85% + ^{4}F 7% + Other 8%
25,001	2.6	^{6}P 9% + ^{6}D 78% + ^{6}F 5% + Other 7%
25,031	3.1	^{6}P 13% + ^{6}D 71% + ^{6}F 6% + Other 10%
25,106	3.3	^{6}P 16% + ^{6}D 67% + ^{6}F 6% + Other 11%
25,147	3.2	^{6}P 14% + ^{6}D 70% + ^{6}F 6% + Other 10%
25,236	2.8	^{6}P 9% + ^{6}D 79% + Other 12%

Table S20. Energy (cm^{-1}) and composition of the excited states less than **29,000 cm^{-1}** for **$[Cm^{III}(AsOPh_3)_4(NO_3)_2]^+$**. The top text corresponds to LFDFT-PBE results and the text in parentheses to LFDFT-PBE0.

**E (cm^{-1})**	**J**	**$^{2S+1}$L Composition**
0<br>(0)	3.5<br>(3.5)	^{8}S 81% + ^{6}P 16% + Other 3%<br>(^{8}S 82% + ^{6}P 15% + Other 3%)
9<br>(7)	3.5<br>(3.5)	^{8}S 81% + ^{6}P 16% + Other 3%<br>(^{8}S 82% + ^{6}P 15% + Other 2%)
17<br>(16)	3.5<br>(3.5)	^{8}S 81% + ^{6}P 16% + Other 3%<br>(^{8}S 82% + ^{6}P 15% + Other 2%)
26<br>(25)	3.5<br>(3.5)	^{8}S 81% + ^{6}P 16% + Other 3%<br>(^{8}S 82% + ^{6}P 15% + Other 2%)
17,398<br>(18,661)	3.5<br>(3.5)	^{8}S 9% + ^{6}P 14% + ^{6}I 6% + ^{6}D 12% + ^{4}D 16% + ^{4}H 7% + ^{4}F 8% + ^{2}F 7% + Other 20%<br>(^{8}S 9% + ^{6}P 17% + ^{6}I 6% + ^{6}D 14% + ^{4}D 17% + ^{4}H 6% + ^{4}F 8% + ^{2}F 6% + Other 18%)
17,798<br>(18,981)	3.5<br>(3.5)	^{8}S 10% + ^{6}P 16% + ^{6}I 5% + ^{6}D 13% + ^{4}D 17% + ^{4}H 6% + ^{4}F 8% + ^{2}F 7% + Other 18%<br>(^{8}S 10% + ^{6}P 18% + ^{6}D 15% + ^{4}D 18% + ^{4}F 7% + ^{2}F 6% + Other 26%)
17,937<br>(19,105)	3.5<br>(3.5)	^{8}S 10% + ^{6}P 16% + ^{6}I 5% + ^{6}D 13% + ^{4}D 18% + ^{4}H 6% + ^{4}F 8% + ^{2}F 7% + Other 18%<br>(^{8}S 10% + ^{6}P 19% + ^{6}D 14% + ^{4}D 18% + ^{4}F 7% + ^{2}F 6% + Other 26%)
18,334<br>(19,501)	3.5<br>(3.5)	^{8}S 10% + ^{6}P 18% + ^{6}D 15% + ^{4}D 19% + ^{4}F 7% + ^{2}F 7% + Other 24%<br>(^{8}S 10% + ^{6}P 21% + ^{6}D 15% + ^{4}D 19% + ^{4}F 7% + ^{2}F 6% + Other 22%)

20,648<br>(21,779)	2.7<br>(2.6)	^{6}P 44% + ^{6}D 28% + ^{4}D 9% + Other 19%<br>(^{6}P 47% + ^{6}D 28% + ^{4}D 9% + Other 16%)
20,900<br>(21,952)	2.7<br>(2.6)	^{6}P 46% + ^{6}D 28% + ^{4}D 10% + Other 17%<br>(^{6}P 48% + ^{6}D 28% + ^{4}D 9% + Other 15%)
21,093<br>(22,173)	2.7<br>(2.6)	^{6}P 47% + ^{6}D 27% + ^{4}D 9% + Other 17%<br>(^{6}P 49% + ^{6}D 27% + ^{4}D 9% + Other 15%)
21,765<br>(22,914)	3.5<br>(3.5)	^{6}P 8% + ^{6}I 46% + ^{6}D 7% + ^{4}H 23% + Other 17%<br>(^{6}P 8% + ^{6}I 48% + ^{6}D 6% + ^{4}H 23% + Other 15%)
21,896<br>(23,044)	3.5<br>(3.5)	^{6}P 9% + ^{6}I 45% + ^{6}D 7% + ^{4}H 22% + Other 17%<br>(^{6}P 8% + ^{6}I 48% + ^{6}D 7% + ^{4}H 22% + Other 15%)
22,007<br>(23,114)	3.5<br>(3.5)	^{6}P 10% + ^{6}I 43% + ^{6}D 9% + ^{4}H 21% + Other 17%<br>(^{6}P 9% + ^{6}I 47% + ^{6}D 7% + ^{4}H 22% + Other 15%)
22,139<br>(23,266)	3.5<br>(3.5)	^{6}P 9% + ^{6}I 46% + ^{6}D 7% + ^{4}H 23% + Other 15%<br>(^{6}P 8% + ^{6}I 49% + ^{6}D 6% + ^{4}H 23% + Other 13%)
22,793<br>(23,885)	2.5<br>(2.0)	^{6}P 50% + ^{6}I 12% + ^{6}D 18% + ^{4}S 6% + Other 15%<br>(^{6}P 59% + ^{6}I 5% + ^{6}D 20% + ^{4}S 6% + Other 9%)
22,883<br>(23,972)	2.3<br>(2.1)	^{6}P 54% + ^{6}I 9% + ^{6}D 19% + ^{4}S 6% + Other 12%<br>(^{6}P 58% + ^{6}I 6% + ^{6}D 19% + ^{4}S 6% + Other 10%)
23,044<br>(24,251)	4.4<br>(4.4)	^{6}I 49% + ^{6}D 6% + ^{4}H 19% + Other 26%<br>(^{6}I 54% + ^{6}D 5% + ^{4}H 19% + Other 22%)
23,205<br>(24,385)	4.3<br>(4.5)	^{6}P 5% + ^{6}I 50% + ^{6}D 6% + ^{4}H 19% + Other 20%<br>(^{6}I 56% + ^{4}H 20% + Other 25%)
23,395<br>(24,559)	4.3<br>(4.4)	^{6}P 6% + ^{6}I 49% + ^{6}D 7% + ^{4}H 18% + Other 20%<br>(^{6}I 54% + ^{6}D 6% + ^{4}H 19% + Other 22%)
23,595<br>(24,746)	4.5<br>(4.5)	^{6}I 53% + ^{6}D 6% + ^{4}H 19% + Other 22%<br>(^{6}I 57% + ^{6}D 5% + ^{4}H 19% + Other 19%)
23,672<br>(24,833)	4.5<br>(4.5)	^{6}I 53% + ^{6}D 6% + ^{4}H 20% + Other 21%<br>(^{6}I 58% + ^{4}H 20% + Other 22%)
24,479<br>(25,648)	8.1<br>(8.2)	^{6}I 68% + ^{4}K 19% + Other 13%<br>(^{6}I 71% + ^{4}K 18% + Other 10%)
24,520<br>(25,679)	8.3<br>(8.4)	^{6}I 69% + ^{4}K 20% + Other 11%<br>(^{6}I 71% + ^{4}K 20% + Other 9%)
24,530<br>(25,686)	8.4<br>(8.4)	^{6}I 69% + ^{4}K 21% + Other 10%<br>(^{6}I 71% + ^{4}K 20% + Other 8%)
24,546<br>(25,696)	8.4<br>(8.4)	^{6}I 69% + ^{4}K 21% + Other 10%<br>(^{6}I 72% + ^{4}K 20% + Other 8%)
24,561<br>(25,704)	8.4<br>(8.4)	^{6}I 70% + ^{4}K 21% + Other 9%<br>(^{6}I 72% + ^{4}K 20% + Other 8%)
24,580<br>(25,723)	8.4<br>(8.4)	^{6}I 70% + ^{4}K 21% + Other 9%<br>(^{6}I 72% + ^{4}K 20% + Other 8%)
24,618<br>(25,754)	8.3<br>(8.3)	^{6}I 70% + ^{4}K 20% + Other 10%<br>(^{6}I 72% + ^{4}K 19% + Other 9%)
24,658<br>(25,788)	8.4<br>(8.5)	^{6}I 71% + ^{4}K 21% + Other 8%<br>(^{6}I 73% + ^{4}K 20% + Other 7%)
24,678<br>(25,811)	8.4<br>(8.5)	^{6}I 71% + Other 29%<br>(^{6}I 73% + ^{4}K 20% + Other 7%)
24,914<br>(26,053)	6.1<br>(5.9)	^{6}I 71% + ^{4}H 11% + ^{4}K 5% + Other 12%<br>(^{6}I 73% + ^{4}H 11% + Other 15%)
24,986<br>(26,107)	5.9<br>(5.8)	^{6}I 71% + ^{4}H 12% + Other 17%<br>(^{6}I 74% + ^{4}H 12% + Other 15%)

25,051<br>(26,183)	5.7<br>(5.7)	^{6}I 71% + ^{4}H 13% + Other 16%<br>(^{6}I 74% + ^{4}H 12% + Other 14%)
25,221<br>(26,336)	5.6<br>(5.6)	^{6}I 72% + ^{4}H 13% + Other 14%<br>(^{6}I 75% + ^{4}H 13% + Other 13%)
25,325<br>(26,406)	5.6<br>(5.6)	^{6}I 73% + ^{4}H 13% + Other 13%<br>(^{6}I 75% + ^{4}H 13% + Other 12%)
25,364<br>(26,466)	5.5<br>(5.5)	^{6}I 73% + ^{4}H 14% + Other 13%<br>(^{6}I 76% + ^{4}H 13% + Other 11%)
25,673<br>(26,798)	6.4<br>(6.5)	^{6}I 71% + ^{4}H 6% + ^{4}K 5% + Other 18%<br>(^{6}I 74% + ^{4}H 5% + ^{4}K 5% + Other 15%)
25,775<br>(26,901)	6.5<br>(6.5)	^{6}I 75% + ^{4}H 6% + ^{4}K 6% + Other 13%<br>(^{6}I 77% + ^{4}H 6% + ^{4}K 5% + Other 11%)
25,829<br>(26,936)	6.5<br>(6.5)	^{6}I 76% + ^{4}H 7% + ^{4}K 6% + Other 12%<br>(^{6}I 78% + ^{4}H 6% + ^{4}K 5% + Other 11%)
25,908<br>(27,032)	6.9<br>(6.7)	^{6}I 75% + ^{4}K 8% + Other 17%<br>(^{6}I 77% + ^{4}K 7% + Other 16%)
25,953<br>(27,074)	6.9<br>(7.2)	^{6}I 76% + ^{4}K 8% + Other 16%<br>(^{6}I 80% + ^{4}K 9% + Other 11%)
25,979<br>(27,079)	7.1<br>(6.9)	^{6}I 77% + ^{4}K 10% + Other 14%<br>(^{6}I 78% + ^{4}K 8% + Other 14%)
25,987<br>(27,109)	7.1<br>(7.2)	^{6}I 77% + ^{4}K 10% + Other 14%<br>(^{6}I 80% + ^{4}K 9% + Other 11%)
26,022<br>(27,134)	6.6<br>(6.7)	^{6}I 76% + ^{4}H 6% + ^{4}K 6% + Other 11%<br>(^{6}I 78% + ^{4}K 7% + Other 15%)
26,047<br>(27,150)	6.7<br>(6.8)	^{6}I 76% + ^{4}H 5% + ^{4}K 7% + Other 12%<br>(^{6}I 79% + ^{4}K 7% + Other 14%)
26,064<br>(27,166)	6.6<br>(6.6)	^{6}I 75% + ^{4}H 6% + ^{4}K 7% + Other 12%<br>(^{6}I 78% + ^{4}H 6% + ^{4}K 6% + Other 10%)
26,142<br>(27,238)	7.2<br>(7.3)	^{6}I 78% + ^{4}K 10% + Other 12%<br>(^{6}I 80% + ^{4}K 10% + Other 10%)
26,169<br>(27,259)	7.3<br>(7.3)	^{6}I 78% + ^{4}K 11% + Other 11%<br>(^{6}I 80% + ^{4}K 10% + Other 9%)
26,240<br>(27,338)	7.3<br>(7.3)	^{6}I 76% + ^{4}K 11% + Other 13%<br>(^{6}I 79% + ^{4}K 11% + Other 10%)
26,317<br>(27,365)	7.4<br>(7.4)	^{6}I 80% + ^{4}F 12% + Other 9%<br>(^{6}I 82% + ^{4}K 11% + Other 7%)
26,329<br>(27,397)	7.4<br>(7.4)	^{6}I 79% + ^{4}F 12% + Other 9%<br>(^{6}I 82% + ^{4}K 11% + Other 7%)
26,524<br>(27,704)	5.3<br>(5.0)	^{6}I 31% + ^{6}D 37% + ^{6}F 10% + ^{4}F 8% + Other 13%<br>(^{6}I 25% + ^{6}D 43% + ^{6}F 11% + ^{4}F 9% + Other 12%)
26,677<br>(27,900)	5.0<br>(4.8)	^{6}I 24% + ^{6}D 43% + ^{6}F 11% + ^{4}F 9% + Other 12%<br>(^{6}I 19% + ^{6}D 49% + ^{6}F 12% + ^{4}F 10% + Other 11%)
26,884<br>(28,128)	4.7<br>(4.6)	^{6}I 20% + ^{6}D 47% + ^{6}F 11% + ^{4}F 11% + Other 11%<br>(^{6}I 16% + ^{6}D 52% + ^{6}F 11% + ^{4}F 11% + Other 10%)
27,098<br>(28,228)	4.7<br>(4.7)	^{6}I 19% + ^{6}D 49% + ^{6}F 10% + ^{4}F 11% + Other 11%<br>(^{6}I 17% + ^{6}D 51% + ^{6}F 10% + ^{4}F 11% + Other 10%)
27,206<br>(28,388)	4.7<br>(4.6)	^{6}I 19% + ^{6}D 50% + ^{6}F 9% + ^{4}F 12% + Other 10%<br>(^{6}I 16% + ^{6}D 53% + ^{6}F 10% + ^{4}F 12% + Other 9%)

Table S21. Energy (cm^{-1}) and composition of the excited states less than **20,000 cm^{-1}** for [**Bk^{IV}(AsOPh$_3$)$_4$(NO$_3$)$_2$]$^{2+}$**. The top text corresponds to LFDFT-PBE results and the text in parentheses to LFDFT-PBE0.

E (cm^{-1})	J	$^{2S+1}$L Composition
0<br>(0)	3.5<br>(3.5)	^{8}S 64% + ^{6}P 25% + ^{4}D 6% + Other 5%<br>(^{8}S 68% + ^{6}P 24% + Other 8%)
53<br>(8)	3.5<br>(3.5)	^{8}S 65% + ^{6}P 25% + ^{4}D 5% + Other 5%<br>(^{8}S 68% + ^{6}P 24% + Other 8%)
115<br>(66)	3.5<br>(3.5)	^{8}S 66% + ^{6}P 25% + ^{4}D 5% + Other 4%<br>(^{8}S 69% + ^{6}P 24% + Other 8%)
177<br>(124)	3.5<br>(3.5)	^{8}S 67% + ^{6}P 25% + Other 8%<br>(^{8}S 70% + ^{6}P 23% + Other 7%)
8,886<br>(10,329)	3.6<br>(3.5)	^{8}S 14% + ^{6}I 8% + ^{6}D 5% + ^{4}D 11% + ^{4}H 12% + ^{4}F 9% + ^{4}G 6% + ^{2}G 8% + ^{2}F 8% + Other 19%<br>(^{8}S 13% + ^{6}I 7% + ^{6}D 7% + ^{4}D 12% + ^{4}H 11% + ^{4}F 9% + ^{4}G 6% + ^{2}G 7% + ^{2}F 8% + Other 21%)
9,624<br>(10,627)	3.6<br>(3.5)	^{8}S 14% + ^{6}I 7% + ^{6}D 6% + ^{4}D 13% + ^{4}H 11% + ^{4}F 9% + ^{4}G 5% + ^{2}G 8% + ^{2}F 9% + Other 19%<br>(^{8}S 13% + ^{6}I 7% + ^{6}D 7% + ^{4}D 12% + ^{4}H 10% + ^{4}F 9% + ^{4}G 5% + ^{2}G 7% + ^{2}F 9% + Other 20%)
10,261<br>(11,277)	3.6<br>(3.5)	^{8}S 14% + ^{6}I 7% + ^{6}D 7% + ^{4}D 14% + ^{4}H 10% + ^{4}F 9% + ^{4}G 5% + ^{2}G 8% + ^{2}F 9% + Other 18%<br>(^{8}S 13% + ^{6}I 7% + ^{6}D 8% + ^{4}D 13% + ^{4}H 10% + ^{4}F 9% + ^{2}G 8% + ^{2}F 9% + Other 24%)
10,983<br>(12,000)	3.6<br>(3.5)	^{8}S 14% + ^{6}I 6% + ^{6}D 8% + ^{4}D 15% + ^{4}H 9% + ^{4}F 9% + ^{2}G 8% + ^{2}F 9% + Other 21%<br>(^{8}S 13% + ^{6}P 6% + ^{6}I 6% + ^{6}D 9% + ^{4}D 15% + ^{4}H 9% + ^{4}F 9% + ^{2}G 7% + ^{2}F 9% + Other 18%)
12,892<br>(14,196)	2.9<br>(2.8)	^{6}P 27% + ^{6}D 24% + ^{6}F 7% + ^{4}D 14% + ^{4}F 7% + Other 21%<br>(^{6}P 31% + ^{6}D 26% + ^{6}F 7% + ^{4}D 11% + ^{4}F 6% + Other 18%)
13,407<br>(14,517)	3.2<br>(2.8)	^{6}P 24% + ^{6}I 11% + ^{6}D 21% + ^{6}F 5% + ^{4}D 12% + ^{4}H 6% + ^{4}F 5% + Other 15%<br>(^{6}P 32% + ^{6}D 26% + ^{6}F 6% + ^{4}D 12% + ^{4}F 6% + Other 18%)
13,768<br>(14,993)	3.8<br>(3.3)	^{6}P 11% + ^{6}I 22% + ^{6}D 14% + ^{4}D 6% + ^{4}H 15% + ^{4}F 5% + Other 27%<br>(^{6}P 23% + ^{6}I 15% + ^{6}D 19% + ^{4}D 9% + ^{4}H 10% + Other 24%)
13,871<br>(15,169)	3.9<br>(3.7)	^{6}P 7% + ^{6}I 26% + ^{6}D 12% + ^{4}H 19% + Other 36%<br>(^{6}P 10% + ^{6}I 28% + ^{6}D 13% + ^{4}H 19% + Other 31%)
14,137<br>(15,294)	3.3<br>(3.6)	^{6}P 25% + ^{6}I 12% + ^{6}D 21% + ^{4}D 11% + ^{4}H 7% + Other 24%<br>(^{8}S 5% + ^{6}P 10% + ^{6}I 28% + ^{6}D 13% + ^{4}H 19% + Other 25%)
14,378<br>(15,448)	3.7<br>(3.8)	^{6}P 11% + ^{6}I 25% + ^{6}D 14% + ^{4}D 6% + ^{4}H 17% + Other 26%<br>(^{6}P 12% + ^{6}I 24% + ^{6}D 15% + ^{4}H 16% + Other 33%)
14,558<br>(15,660)	4.2<br>(3.8)	^{6}P 5% + ^{6}I 28% + ^{6}D 11% + ^{4}H 19% + ^{4}F 6% + ^{4}G 6% + Other 25%<br>(^{6}P 8% + ^{6}I 31% + ^{6}D 12% + ^{4}H 20% + Other 29%)
14,795<br>(15,836)	4.0<br>(4.2)	^{6}P 7% + ^{6}I 29% + ^{6}D 12% + ^{4}H 19% + Other 32%<br>(^{6}I 34% + ^{6}D 8% + ^{4}H 21% + ^{4}F 5% + ^{4}G 6% + Other 25%)
15,023<br>(16,020)	4.0<br>(4.0)	^{6}P 9% + ^{6}I 28% + ^{6}D 12% + ^{4}H 18% + Other 33%<br>(^{6}P 6% + ^{6}I 34% + ^{6}D 10% + ^{4}H 21% + Other 29%)
15,172<br>(16,293)	4.2<br>(4.2)	^{6}P 7% + ^{6}I 29% + ^{6}D 11% + ^{4}H 18% + ^{4}F 5% + ^{4}G 5% + Other 24%<br>(^{6}P 8% + ^{6}I 30% + ^{6}D 11% + ^{4}H 18% + ^{4}F 6% + ^{4}G 6% + Other 22%)
15,504<br>(16,632)	3.8<br>(3.8)	^{6}P 23% + ^{6}I 19% + ^{6}D 15% + ^{4}H 10% + Other 33%<br>(^{6}P 20% + ^{6}I 23% + ^{6}D 14% + ^{4}H 13% + Other 30%)
15,641<br>(16,796)	4.6<br>(3.8)	^{6}P 17% + ^{6}I 26% + ^{6}D 12% + ^{4}H 11% + Other 35%<br>(^{6}P 21% + ^{6}I 23% + ^{6}D 14% + ^{4}H 12% + Other 30%)
15,782<br>(16,900)	4.8<br>(3.2)	^{6}P 9% + ^{6}I 31% + ^{6}D 10% + ^{4}H 15% + ^{4}F 5% + Other 30%<br>(^{6}P 33% + ^{6}I 15% + ^{6}D 17% + ^{4}H 8% + ^{4}S 6% + Other 21%)
15,874<br>(17,011)	4.5<br>(3.9)	^{6}P 20% + ^{6}I 24% + ^{6}D 12% + ^{4}H 10% + Other 34%<br>(^{6}P 19% + ^{6}I 26% + ^{6}D 13% + ^{4}H 14% + Other 28%)
16,073<br>(17,395)	7.0<br>(7.7)	^{6}I 41% + ^{4}H 6% + ^{4}K 16% + Other 36%<br>(^{6}I 49% + ^{4}K 20% + ^{2}L 5% + Other 25%)

16,124<br>(17,437)	7.2<br>(7.8)	^{6}I 41% + ^{4}H 5% + ^{4}K 18% + ^{2}L 5% + Other 31%<br>(^{6}I 50% + ^{4}K 22% + ^{2}L 6% + Other 23%)
16,206<br>(17,495)	7.5<br>(8.1)	^{6}I 43% + ^{4}H 5% + ^{4}K 20% + ^{2}L 6% + Other 26%<br>(^{6}I 50% + ^{4}K 25% + ^{2}L 7% + Other 19%)
16,270<br>(17,535)	7.9<br>(7.9)	^{6}I 45% + ^{4}K 24% + ^{2}L 7% + Other 24%<br>(^{6}I 51% + ^{4}K 23% + ^{2}L 6% + Other 21%)
16,322<br>(17,571)	8.0<br>(8.2)	^{6}I 46% + ^{4}K 24% + ^{2}L 7% + Other 22%<br>(^{6}I 52% + ^{4}K 25% + ^{2}L 7% + Other 16%)
16,394<br>(17,588)	8.2<br>(8.1)	^{6}I 47% + ^{4}K 26% + ^{2}L 8% + Other 20%<br>(^{6}I 52% + ^{4}K 24% + ^{2}L 6% + Other 17%)
16,496<br>(17,604)	8.2<br>(8.2)	^{6}I 49% + ^{4}K 26% + ^{2}L 7% + Other 18%<br>(^{6}I 52% + ^{4}K 25% + ^{2}L 7% + Other 17%)
16,649<br>(17,888)	7.4<br>(8.1)	^{6}I 49% + ^{4}H 5% + ^{4}K 18% + Other 28%<br>(^{6}I 55% + ^{4}K 24% + ^{2}L 6% + Other 15%)
16,755<br>(17,919)	7.7<br>(8.3)	^{6}I 48% + ^{4}K 22% + ^{2}L 6% + Other 24%<br>(^{6}I 55% + ^{4}K 26% + ^{2}L 7% + Other 11%)
16,871<br>(18,075)	7.2<br>(6.6)	^{6}I 50% + ^{4}H 6% + ^{4}K 17% + Other 27%<br>(^{6}I 55% + ^{4}H 10% + ^{4}K 11% + Other 24%)
17,003<br>(18,173)	6.7<br>(6.6)	^{6}I 51% + ^{4}H 9% + ^{4}K 13% + Other 27%<br>(^{6}I 56% + ^{4}H 11% + ^{4}K 11% + Other 22%)
17,098<br>(18,220)	6.5<br>(6.2)	^{6}I 50% + ^{4}H 9% + ^{4}K 12% + Other 29%<br>(^{6}I 55% + ^{4}H 12% + ^{4}K 8% + Other 24%)
17,303<br>(18,410)	6.0<br>(6.0)	^{6}I 51% + ^{4}H 13% + ^{4}K 7% + Other 29%<br>(^{6}I 54% + ^{4}H 13% + ^{4}K 6% + Other 26%)
17,432<br>(18,622)	5.8<br>(5.7)	^{6}I 49% + ^{6}D 5% + ^{4}H 12% + ^{4}K 6% + Other 28%<br>(^{6}I 55% + ^{4}H 14% + Other 31%)
17,583<br>(18,755)	5.9<br>(5.7)	^{6}I 52% + ^{4}H 13% + ^{4}K 6% + Other 29%<br>(^{6}I 52% + ^{6}D 7% + ^{4}H 12% + Other 29%)
17,631<br>(18,782)	6.0<br>(5.7)	^{6}I 51% + ^{4}H 12% + ^{4}K 7% + Other 31%<br>(^{6}I 56% + ^{4}H 14% + Other 30%)
17,738<br>(18,940)	6.3<br>(6.2)	^{6}I 52% + ^{4}H 9% + ^{4}K 8% + Other 31%<br>(^{6}I 57% + ^{4}H 10% + ^{4}K 7% + Other 27%)
17,829<br>(19,050)	6.3<br>(6.5)	^{6}I 53% + ^{4}H 10% + ^{4}K 8% + Other 29%<br>(^{6}I 59% + ^{4}H 9% + ^{4}K 8% + Other 24%)
17,881<br>(19,116)	6.3<br>(6.5)	^{6}I 52% + ^{4}H 9% + ^{4}K 8% + Other 31%<br>(^{6}I 57% + ^{4}H 8% + ^{4}K 9% + Other 27%)
17,941<br>(19,166)	6.5<br>(6.3)	^{6}I 54% + ^{4}H 9% + ^{4}K 9% + Other 29%<br>(^{6}I 56% + ^{4}H 9% + ^{4}K 7% + Other 28%)
18,009<br>(19,235)	6.5<br>(6.6)	^{6}I 53% + ^{4}H 9% + ^{4}K 9% + Other 30%<br>(^{6}I 58% + ^{4}H 8% + ^{4}K 9% + Other 25%)
18,071<br>(19,266)	6.5<br>(6.5)	^{6}I 52% + ^{4}H 7% + ^{4}K 10% + Other 31%<br>(^{6}I 56% + ^{4}H 8% + ^{4}K 9% + Other 27%)
18,113<br>(19,350)	6.4<br>(6.6)	^{6}I 55% + ^{4}H 10% + ^{4}K 8% + Other 27%<br>(^{6}I 58% + ^{4}H 7% + ^{4}K 9% + Other 25%)
18,187<br>(19,385)	6.6<br>(6.8)	^{6}I 55% + ^{4}H 8% + ^{4}K 10% + Other 27%<br>(^{6}I 61% + ^{4}H 5% + ^{4}K 11% + Other 23%)
18,247<br>(19,435)	6.7<br>(6.7)	^{6}I 56% + ^{4}H 5% + ^{4}K 11% + Other 28%<br>(^{6}I 59% + ^{4}H 6% + ^{4}K 10% + Other 25%)
18,345<br>(19,473)	6.3<br>(6.4)	^{6}I 51% + ^{6}D 7% + ^{4}H 7% + ^{4}K 9% + Other 27%<br>(^{6}I 55% + ^{6}D 7% + ^{4}H 7% + ^{4}K 8% + Other 23%)

18,414<br>(19,553)	6.4<br>(6.8)	^{6}I 49% + ^{6}D 10% + ^{4}K 9% + Other 32%<br>(^{6}I 58% + ^{6}D 6% + ^{4}K 11% + Other 24%)
18,510<br>(19,654)	6.8<br>(6.9)	^{6}I 57% + ^{4}K 13% + Other 31%<br>(^{6}I 61% + ^{4}K 13% + Other 27%)
18,698<br>(19,766)	6.7<br>(7.0)	^{6}I 52% + ^{6}D 9% + ^{4}K 12% + Other 26%<br>(^{6}I 60% + ^{6}D 5% + ^{4}K 14% + Other 21%)
18,734<br>(19,836)	6.8<br>(6.9)	^{6}I 55% + ^{6}D 6% + ^{4}K 14% + Other 25%<br>(^{6}I 60% + ^{6}D 6% + ^{4}K 13% + Other 21%)
18,809<br>(19,875)	6.7<br>(7.1)	^{6}I 53% + ^{6}D 8% + ^{4}K 13% + Other 26%<br>(^{6}I 63% + ^{4}K 15% + Other 23%)
18,881<br>(19,928)	7.1<br>(7.0)	^{6}I 61% + ^{4}K 15% + Other 24%<br>(^{6}I 61% + ^{6}D 6% + ^{4}K 14% + Other 19%)
18,959<br>(20,075)	5.8<br>(5.4)	^{6}I 40% + ^{6}D 20% + ^{6}F 7% + ^{4}K 7% + ^{4}F 6% + Other 20%<br>(^{6}I 33% + ^{6}D 28% + ^{6}F 9% + ^{4}F 8% + Other 22%)
19,216<br>(20,308)	5.2<br>(5.0)	^{6}I 30% + ^{6}D 28% + ^{6}F 8% + ^{4}F 9% + Other 25%<br>(^{6}I 29% + ^{6}D 33% + ^{6}F 9% + ^{4}F 10% + Other 19%)
19,416<br>(20,360)	5.2<br>(5.2)	^{6}I 31% + ^{6}D 30% + ^{6}F 6% + ^{4}F 10% + Other 22%<br>(^{6}I 32% + ^{6}D 32% + ^{6}F 8% + ^{4}F 10% + Other 19%)
19,723<br>(21,031)	4.3<br>(4.0)	^{6}P 6% + ^{6}I 12% + ^{6}D 17% + ^{6}H 8% + ^{6}F 10% + ^{4}D 16% + ^{2}F 7% + Other 25%<br>(^{6}P 7% + ^{6}I 7% + ^{6}D 18% + ^{6}G 9% + ^{6}F 12% + ^{4}D 14% + ^{2}F 7% + Other 26%)
19,916<br>(21,170)	4.2<br>(3.9)	^{6}P 6% + ^{6}I 11% + ^{6}D 20% + ^{6}H 6% + ^{6}F 10% + ^{4}D 18% + ^{2}F 8% + Other 21%<br>(^{6}P 6% + ^{6}I 6% + ^{6}D 24% + ^{6}G 6% + ^{6}F 12% + ^{4}D 20% + ^{2}F 8% + Other 17%)

Table S22. Energy (cm^{-1}) and composition of the excited states less than **43,000 cm^{-1}** for [**Gd^{III}F**$_6$]$^{3-}$. The top text corresponds to LFDFT-PBE results and the text in parentheses to LFDFT-PBE0.

**E (cm^{-1})**	**J**	**$^{2S+1}$L Composition**
0<br>(0)	3.5<br>(3.5)	^{8}S 97% + Other 3%<br>(^{8}S 97% + Other 3%)
32,718<br>(33,416)	3.5<br>(3.5)	^{6}P 76% + ^{6}D 12% + ^{4}D 7% + Other 5%<br>(^{6}P 77% + ^{6}D 11% + ^{4}D 7% + Other 4%)
32,756<br>(33,438)	3.5<br>(3.5)	^{6}P 76% + ^{6}D 12% + ^{4}D 7% + Other 5%<br>(^{6}P 78% + ^{6}D 11% + ^{4}D 7% + Other 4%)
32,783<br>(33,454)	3.5<br>(3.5)	^{6}P 77% + ^{6}D 11% + ^{4}D 7% + Other 4%<br>(^{6}P 78% + ^{6}D 11% + ^{4}D 7% + Other 4%)
33,293<br>(33,985)	2.6<br>(2.5)	^{6}P 83% + ^{6}D 13% + Other 4%<br>(^{6}P 85% + ^{6}D 12% + Other 3%)
33,343<br>(34,013)	2.6<br>(2.5)	^{6}P 84% + ^{6}D 12% + Other 4%<br>(^{6}P 85% + ^{6}D 12% + Other 3%)
33,875<br>(34,546)	1.6<br>(1.5)	^{6}P 90% + ^{6}D 7% + Other 3%<br>(^{6}P 91% + ^{6}D 7% + Other 2%)
35,836<br>(36,581)	3.5<br>(3.5)	^{6}I 92% + ^{4}H 7% + Other 2%<br>(^{6}I 93% + ^{4}H 6% + Other 1%)
35,948<br>(36,642)	3.5<br>(3.5)	^{6}I 92% + ^{4}H 7% + Other 1%<br>(^{6}I 93% + ^{4}H 6% + Other 1%)
36,013<br>(36,679)	3.5<br>(3.5)	^{6}I 93% + ^{4}H 7% + Other 1%<br>(^{6}I 93% + ^{4}H 6% + Other 1%)

36,224<br>(36,956)	4.5<br>(4.5)	^{6}I 94% + Other 6%<br>(^{6}I 95% + Other 5%)
36,352<br>(37,024)	4.6<br>(4.5)	^{6}I 94% + Other 6%<br>(^{6}I 95% + Other 5%)
36,393<br>(37,050)	4.6<br>(4.5)	^{6}I 95% + Other 5%<br>(^{6}I 95% + Other 5%)
36,464<br>(37,201)	6.9<br>(8.1)	^{6}I 95% + Other 5%<br>(^{6}I 96% + Other 4%)
36,497<br>(37,205)	8.4<br>(8.5)	^{6}I 96% + Other 4%<br>(^{6}I 96% + Other 4%)
36,530<br>(37,219)	8.4<br>(8.5)	^{6}I 96% + Other 4%<br>(^{6}I 96% + Other 4%)
36,544<br>(37,258)	8.4<br>(6.1)	^{6}I 95% + Other 5%<br>(^{6}I 96% + Other 4%)
36,559<br>(37,279)	7.5<br>(5.7)	^{6}I 96% + Other 4%<br>(^{6}I 96% + Other 4%)
36,578<br>(37,331)	6.1<br>(5.5)	^{6}I 96% + Other 4%<br>(^{6}I 96% + Other 4%)
36,666<br>(37,351)	5.6<br>(5.6)	^{6}I 96% + Other 4%<br>(^{6}I 96% + Other 4%)
36,696<br>(37,436)	5.8<br>(6.5)	^{6}I 96% + Other 4%<br>(^{6}I 97% + Other 3%)
36,708<br>(37,459)	6.6<br>(7.2)	^{6}I 96% + Other 4%<br>(^{6}I 97% + Other 3%)
36,759<br>(37,468)	7.1<br>(6.8)	^{6}I 97% + Other 3%<br>(^{6}I 97% + Other 3%)
36,782<br>(37,491)	6.7<br>(7.5)	^{6}I 96% + Other 4%<br>(^{6}I 97% + Other 3%)
36,815<br>(37,511)	7.5<br>(7.5)	^{6}I 97% + Other 3%<br>(^{6}I 97% + Other 3%)
36,825<br>(37,545)	6.6<br>(7.5)	^{6}I 97% + Other 3%<br>(^{6}I 97% + Other 3%)
36,855<br>(41,431)	7.4<br>(4.5)	^{6}I 97% + Other 3%<br>(^{6}D 92% + Other 8%)
36,895<br>(41,500)	7.5<br>(4.5)	^{6}I 97% + Other 3%<br>(^{6}D 93% + Other 7%)
36,900<br>(41,629)	7.2<br>(4.5)	^{6}I 97% + Other 3%<br>(^{6}D 93% + Other 7%)
40,546<br>(42,348)	4.5<br>(1.2)	^{6}D 91% + Other 9%<br>(^{6}D 97% + Other 3%)
40,673<br>(42,449)	4.5<br>(3.1)	^{6}D 92% + Other 8%<br>(^{6}P 10% + ^{6}D 84% + Other 7%)
40,880<br>(42,460)	4.5<br>(3.4)	^{6}D 92% + Other 8%<br>(^{6}P 11% + ^{6}D 81% + Other 7%)
41,552<br>(42,524)	1.7<br>(3.3)	^{6}D 95% + Other 5%<br>(^{6}P 9% + ^{6}D 84% + Other 6%)

41,614<br>(42,650)	3.0<br>(2.2)	^{6}P 10% + ^{6}D 83% + Other 7%<br>(^{6}P 8% + ^{6}D 89% + Other 4%)
41,626<br>(42,674)	3.2<br>(2.6)	^{6}P 12% + ^{6}D 79% + Other 8%<br>(^{6}P 12% + ^{6}D 82% + Other 5%)
41,780<br>(42,817)	3.1<br>(2.5)	^{6}P 9% + ^{6}D 85% + Other 6%<br>(^{6}P 12% + ^{6}D 84% + Other 4%)
41,879	2.9	^{6}P 12% + ^{6}D 82% + Other 6%
41,936	2.4	^{6}P 8% + ^{6}D 88% + Other 4%
42,103	2.6	^{6}P 12% + ^{6}D 84% + Other 5%

Table S23. Energy (cm^{-1}) and composition of the excited states less than **31,000 cm^{-1}** for **[$Tb^{IV}F_6$]$^{2-}$**. The top text corresponds to LFDFT-PBE results and the text in parentheses to LFDFT-PBE0.

E (cm^{-1})	J	$^{2S+1}$L Composition
0<br>(0)	3.5<br>(3.5)	^{8}S 95% + Other 5%<br>(^{8}S 95% + Other 5%)
21,634<br>(22,951)	3.7<br>(3.5)	^{6}P 55% + ^{6}D 16% + ^{4}D 11% + Other 18%<br>(^{6}P 64% + ^{6}D 15% + ^{4}D 11% + Other 9%)
21,811<br>(23,018)	3.8<br>(3.5)	^{6}P 57% + ^{6}D 17% + ^{4}D 10% + Other 16%<br>(^{6}P 66% + ^{6}D 15% + ^{4}D 11% + Other 8%)
21,957<br>(23,063)	3.9<br>(3.5)	^{6}P 60% + ^{6}D 16% + ^{4}D 10% + Other 14%<br>(^{6}P 66% + ^{6}D 15% + ^{4}D 11% + Other 7%)
22,286<br>(23,648)	3.1<br>(2.5)	^{6}P 64% + ^{6}I 5% + ^{6}D 19% + Other 12%<br>(^{6}P 76% + ^{6}D 18% + Other 6%)
22,578<br>(23,737)	3.1<br>(2.5)	^{6}P 69% + ^{6}I 7% + ^{6}D 15% + Other 8%<br>(^{6}P 78% + ^{6}D 17% +Other 5%)
23,110<br>(24,344)	2.5<br>(1.5)	^{6}P 74% + ^{6}I 8% + ^{6}D 10% + Other 8%<br>(^{6}P 86% + ^{6}D 10% + Other 4%)
23,858<br>(25,408)	3.7<br>(3.5)	^{6}I 76% + ^{4}H 10% + Other 14%<br>(^{6}I 85% + ^{4}H 11% + Other 4%)
24,144<br>(25,549)	4.4<br>(3.5)	^{6}I 79% + ^{4}H 8% + Other 13%<br>(^{6}I 87% + ^{4}H 11% + Other 2%)
24,456<br>(25,637)	5.3<br>(3.5)	^{6}I 79% + ^{6}D 5% + ^{6}H 5% + Other 10%<br>(^{6}I 88% + ^{4}H 11% + Other 1%)
24,635<br>(25,919)	6.3<br>(4.5)	^{6}I 82% + Other 18%<br>(^{6}I 89% + ^{4}H 8% + Other 3%)
24,685<br>(26,064)	6.8<br>(4.6)	^{6}I 82% + Other 18%<br>(^{6}I 91% + ^{4}H 7% + Other 2%)
24,761<br>(26,119)	4.6<br>(4.6)	^{6}I 82% + ^{4}H 8% + Other 11%<br>(^{6}I 91% + ^{4}H 7% + Other 1%)
24,821<br>(26,227)	4.1<br>(7.2)	^{6}I 82% + ^{4}H 9% + Other 9%<br>(^{6}I 92% + Other 8%)
24,859<br>(26,260)	7.9<br>(8.4)	^{6}I 86% + Other 14%<br>(^{6}I 93% + ^{4}K 6% + Other 1%)

25,161<br>(26,275)	6.1<br>(8.3)	^{6}I 88% + Other 12%<br>(^{6}I 93% + ^{4}K 6% + Other 1%)
25,178<br>(26,288)	7.0<br>(8.4)	^{6}I 85% + Other 15%<br>(^{6}I 93% + ^{4}K 6% + Other 1%)
25,316<br>(26,298)	7.2<br>(8.3)	^{6}I 86% + Other 14%<br>(^{6}I 93% + ^{4}K 6% + Other 1%)
25,348<br>(26,326)	7.5<br>(7.3)	^{6}I 85% + Other 15%<br>(^{6}I 93% + Other 7%)
25,388<br>(26,352)	5.9<br>(6.0)	^{6}I 87% + Other 13%<br>(^{6}I 93% + Other 7%)
25,437<br>(26,456)	7.2<br>(5.6)	^{6}I 90% + Other 10%<br>(^{6}I 94% + Other 6%)
25,510<br>(26,487)	7.6<br>(5.8)	^{6}I 89% + Other 11%<br>(^{6}I 94% + Other 6%)
25,591<br>(26,508)	6.7<br>(6.5)	^{6}P 6% + ^{6}I 84% + Other 10%<br>(^{6}I 94% + Other 6%)
25,643<br>(26,565)	7.2<br>(7.3)	^{6}I 90% + Other 10%<br>(^{6}I 94% + Other 6%)
25,724<br>(26,588)	6.0<br>(6.5)	^{6}I 92% + Other 8%<br>(^{6}I 94% + Other 6%)
25,835<br>(26,633)	7.3<br>(7.4)	^{6}I 91% + Other 9%<br>(^{6}I 95% + Other 5%)
25,870<br>(26,648)	7.1<br>(6.6)	^{6}I 93% + Other 7%<br>(^{6}I 95% + Other 5%)
25,891<br>(26,668)	6.8<br>(7.4)	^{6}I 91% + Other 9%<br>(^{6}I 95% + Other 5%)
25,908<br>(26,726)	7.1<br>(6.5)	^{6}I 92% + Other 8%<br>(^{6}I 95% + Other 5%)
25,917<br>(26,730)	7.0<br>(6.5)	^{6}I 92% + Other 8%<br>(^{6}I 95% + Other 5%)
25,968<br>(26,740)	7.2<br>(7.5)	^{6}I 93% + Other 7%<br>(^{6}I 96% + Other 4%)
26,011<br>(28,990)	6.4<br>(4.5)	^{6}I 91% + Other 9%<br>(^{6}D 85% + ^{4}N, ^{6}F 7% + ^{4}F 5% + Other 3%)
27,546<br>(29,117)	4.8<br>(4.5)	^{6}I 18% + ^{6}D 65% + ^{4}N, ^{6}F 8% + Other 9%<br>(^{6}D 86% + ^{4}N, ^{6}F 6% + ^{4}F 5% + Other 3%)
27,888<br>(29,339)	4.8<br>(4.5)	^{6}I 12% + ^{6}D 72% + ^{4}N, ^{6}F 6% + Other 10%<br>(^{6}D 87% + ^{4}N, ^{4}F 6% + Other 7%)
28,246<br>(30,170)	4.6<br>(2.3)	^{6}I 7% + ^{6}D 77% + ^{4}K 5% + Other 11%<br>(^{6}P 5% + ^{6}D 87% + Other 8%)
28,733<br>(30,201)	3.3<br>(3.2)	^{6}P 19% + ^{6}I 8% + ^{6}D 57% + ^{4}N, ^{6}F 8% + Other 8%<br>(^{6}P 14% + ^{6}D 74% + ^{4}N, ^{6}F 5% + Other 7%)
28,793<br>(30,215)	3.4<br>(3.3)	^{6}P 12% + ^{6}D 69% + ^{4}N, ^{6}F 6% + Other 14%<br>(^{6}P 16% + ^{6}D 70% + ^{4}N, ^{6}F 6% + Other 8%)
28,881<br>(30,362)	3.0<br>(2.7)	^{6}D 80% + Other 20%<br>(^{6}P 8% + ^{6}D 83% + Other 9%)

29,289<br>(30,529)	2.9<br>(2.8)	^{6}P 7% + ^{6}D 80% + Other 13%<br>(^{6}P 17% + ^{6}D 73% + Other 10%)
29,344<br>(30,569)	3.3<br>(2.2)	^{6}P 15% + ^{6}D 70% + Other 15%<br>(^{6}P 11% + ^{6}D 83% + Other 6%)
29,564<br>(30,730)	2.7<br>(2.5)	^{6}P 10% + ^{6}D 80% + Other 11%<br>(^{6}P 16% + ^{6}D 76% + Other 8%)
29,762	2.9	^{6}P 14% + ^{6}D 75% + Other 11%

Table S24. Energy (cm^{-1}) and composition of the excited states less than **31,000 cm^{-1}** for [**Cm^{III}F$_6$**]$^{3-}$. The top text corresponds to LFDFT-PBE results and the text in parentheses to LFDFT-PBE0.

E (cm^{-1})	J	$^{2S+1}$L Composition
0<br>(0)	3.5<br>(3.5)	^{8}S 82% + ^{6}P 15% + Other 3%<br>(^{8}S 83% + ^{6}P 15% + Other 2%)
13<br>(9)	3.5<br>(3.5)	^{8}S 82% + ^{6}P 15% + Other 2%<br>(^{8}S 83% + ^{6}P 15% + Other 2%)
22<br>(16)	3.5<br>(3.5)	^{8}S 82% + ^{6}P 15% + Other 2%<br>(^{8}S 83% + ^{6}P 15% + Other 2%)
19,100<br>(20,104)	3.5<br>(3.5)	^{8}S 9% + ^{6}P 15% + ^{6}I 6% + ^{6}D 13% + ^{4}D 18% + ^{4}H 6% + ^{4}F 8% + ^{2}F 6% + Other 19%<br>(^{8}S 9% + ^{6}P 18% + ^{6}I 6% + ^{6}D 14% + ^{4}D 17% + ^{4}H 5% + ^{4}F 8% + ^{2}F 6% + Other 19%)
19,851<br>(20,687)	3.5<br>(3.5)	^{8}S 9% + ^{6}P 19% + ^{6}I 5% + ^{6}D 15% + ^{4}D 19% + ^{4}F 7% + ^{2}F 6% + Other 20%<br>(^{8}S 9% + ^{6}P 21% + ^{6}D 15% + ^{4}D 18% + ^{4}F 7% + ^{2}F 6% + Other 25%)
20,580<br>(21,274)	3.6<br>(3.5)	^{8}S 11% + ^{6}P 23% + ^{6}D 17% + ^{4}D 21% + ^{4}F 7% + ^{2}F 6% + Other 17%<br>(^{8}S 10% + ^{6}P 24% + ^{6}D 17% + ^{4}D 19% + ^{4}F 6% + ^{2}F 5% + Other 17%)
22,395<br>(23,232)	2.7<br>(2.6)	^{6}P 44% + ^{6}D 29% + ^{4}D 11% + Other 16%<br>(^{6}P 47% + ^{6}D 29% + ^{4}D 9% + Other 15%)
23,001<br>(23,719)	2.8<br>(2.7)	^{6}P 45% + ^{6}I 6% + ^{6}D 25% + ^{4}D 10% + Other 13%<br>(^{6}P 48% + ^{6}D 26% + ^{4}D 9% + Other 16%)
23,714<br>(24,520)	3.5<br>(3.5)	^{6}P 10% + ^{6}I 46% + ^{6}D 6% + ^{4}H 21% + Other 17%<br>(^{6}P 9% + ^{6}I 48% + ^{6}D 5% + ^{4}H 21% + Other 15%)
24,008<br>(24,822)	3.5<br>(3.5)	^{6}P 11% + ^{6}I 42% + ^{6}D 10% + ^{4}H 20% + Other 17%<br>(^{6}P 10% + ^{6}I 46% + ^{6}D 8% + ^{4}H 21% + Other 15%)
24,260<br>(24,989)	3.6<br>(3.5)	^{6}I 55% + ^{4}H 25% + ^{2}G 5% + Other 14%<br>(^{6}I 56% + ^{4}H 25% + Other 19%)
24,798<br>(25,546)	2.7<br>(2.3)	^{6}P 48% + ^{6}I 14% + ^{6}D 17% + ^{4}S 5% + Other 16%<br>(^{6}P 56% + ^{6}I 8% + ^{6}D 19% + Other 17%)
25,183<br>(25,991)	4.1<br>(4.3)	^{6}P 11% + ^{6}I 47% + ^{6}D 6% + ^{4}H 17% + Other 19%<br>(^{6}P 5% + ^{6}I 54% + ^{4}H 18% + Other 23%)
25,839<br>(26,561)	4.6<br>(4.5)	^{6}I 56% + ^{6}D 5% + ^{4}H 18% + Other 21%<br>(^{6}I 59% + ^{4}H 18% + Other 23%)
26,183<br>(26,852)	4.8<br>(4.6)	^{6}I 59% + ^{6}D 6% + ^{4}H 18% + Other 17%<br>(^{6}I 62% + ^{4}H 19% + Other 19%)
26,468<br>(27,322)	6.8<br>(7.3)	^{6}I 67% + ^{4}H 8% + ^{4}K 9% + Other 16%<br>(^{6}I 71% + ^{4}H 6% + ^{4}K 12% + Other 12%)
26,661<br>(27,436)	7.8<br>(8.1)	^{6}I 69% + ^{4}K 16% + Other 15%<br>(^{6}I 72% + ^{4}K 17% + Other 11%)

26,721<br>(27,486)	8.3<br>(8.5)	^{6}I 70% + ^{4}K 19% + Other 11%<br>(^{6}I 73% + ^{4}K 19% + Other 8%)
26,727<br>(27,570)	8.4<br>(8.3)	^{6}I 72% + ^{4}K 20% + Other 8%<br>(^{6}I 73% + ^{4}K 18% + Other 9%)
26,834<br>(27,629)	8.2<br>(8.4)	^{6}I 71% + ^{4}K 18% + Other 11%<br>(^{6}I 74% + ^{4}K 19% + Other 7%)
26,919<br>(27,804)	8.4<br>(6.8)	^{6}I 72% + ^{4}K 19% + Other 9%<br>(^{6}I 74% + ^{4}H 7% + ^{4}K 9% + Other 11%)
27,016<br>(27,822)	6.4<br>(6.1)	^{6}I 69% + ^{4}H 7% + ^{4}K 6% + Other 17%<br>(^{6}I 72% + ^{4}H 9% + Other 19%)
27,026<br>(28,178)	7.0<br>(5.6)	^{6}I 70% + ^{4}H 5% + ^{4}K 10% + Other 15%<br>(^{6}I 76% + ^{4}H 12% + Other 11%)
27,485<br>(28,356)	5.6<br>(6.3)	^{6}I 74% + ^{4}H 13% + Other 13%<br>(^{6}I 69% + ^{6}D 7% + Other 23%)
27,517<br>(28,384)	6.4<br>(5.5)	^{6}I 67% + ^{6}D 8% + ^{4}H 5% + ^{4}K 5% + Other 15%<br>(^{6}I 77% + ^{4}H 12% + Other 11%)
27,748<br>(28,641)	5.5<br>(6.6)	^{6}I 75% + ^{4}H 12% + Other 13%<br>(^{6}I 77% + ^{4}K 6% + Other 18%)
27,901<br>(28,764)	6.6<br>(7.1)	^{6}I 75% + ^{4}K 6% + Other 19%<br>(^{6}I 79% + ^{4}K 8% + Other 13%)
28,031<br>(28,862)	7.0<br>(6.5)	^{6}I 77% + ^{4}K 8% + Other 15%<br>(^{6}I 80% + ^{4}H 6% + Other 14%)
28,196<br>(28,905)	6.5<br>(7.4)	^{6}I 78% + ^{4}H 7% + ^{4}K 5% + Other 10%<br>(^{6}I 80% + ^{4}K 10% + Other 11%)
28,208<br>(29,065)	7.3<br>(6.6)	^{6}I 77% + ^{4}K 10% + Other 13%<br>(^{6}I 80% + ^{4}H 6% + ^{4}K 6% + Other 9%)
28,411<br>(29,078)	6.7<br>(6.5)	^{6}I 78% + ^{4}H 6% + ^{4}K 7% + Other 10%<br>(^{6}I 79% + ^{4}H 7% + Other 14%)
28,424<br>(29,122)	6.5<br>(7.4)	^{6}I 77% + ^{4}H 7% + ^{4}K 5% + Other 11%<br>(^{6}I 83% + ^{4}K 10% + Other 8%)
28,481<br>(29,243)	7.3<br>(7.4)	^{6}I 81% + ^{4}K 10% + Other 9%<br>(^{6}I 82% + ^{4}K 11% + Other 7%)
28,588<br>(29,270)	7.4<br>(7.4)	^{6}I 80% + ^{4}K 11% + Other 9%<br>(^{6}I 82% + ^{4}K 11% + Other 7%)
28,638<br>(29,540)	7.4<br>(5.0)	^{6}I 81% + ^{4}K 11% + Other 8%<br>(^{6}I 24% + ^{6}D 43% + ^{6}F 11% + ^{4}F 9% + Other 13%)
28,752	5.2	^{6}I 30% + ^{6}D 37% + ^{6}F 10% + ^{4}F 8% + Other 15%<br>(^{6}I 21% + ^{6}D 49% + ^{6}F 10% + ^{4}F 10% + Other 10%)
29,318	5.1	^{6}I 25% + ^{6}D 45% + ^{6}F 9% + ^{4}F 9% + Other 11%<br>(^{6}I 17% + ^{6}D 53% + ^{6}F 8% + ^{4}F 12% + Other 10%)
29,824	4.8	^{6}I 19% + ^{6}D 51% + ^{6}F 8% + ^{4}F 12% + Other 10%<br>(^{6}P 10% + ^{6}D 44% + ^{6}F 12% + ^{4}D 14% + ^{2}F 6% + Other 15%)

Table S25. Energy (cm^{-1}) and composition of the excited states less than **24,000 cm^{-1}** for **[Bk^{IV}F$_6$]$^{2-}$**. The top text corresponds to LFDFT-PBE results and the text in parentheses to LFDFT-PBE0.

E (cm^{-1})	J	$^{2S+1}$L Composition
0<br>(0)	3.5<br>(3.5)	^{8}S 67% + ^{6}P 23% + Other 10%<br>(^{8}S 71% + ^{6}P 22% + Other 7%)
125<br>(69)	3.5<br>(3.5)	^{8}S 69% + ^{6}P 23% + Other 8%<br>(^{8}S 72% + ^{6}P 22% + Other 6%)
196<br>(114)	3.5<br>(3.5)	^{8}S 70% + ^{6}P 23% + Other 7%<br>(^{8}S 73% + ^{6}P 21% + Other 6%)
9,663<br>(11,738)	3.7<br>(3.6)	^{8}S 12% + ^{6}I 8% + ^{6}D 6% + ^{4}D 8% + ^{4}H 11% + ^{4}F 9% + ^{4}G 7% + ^{2}G 7% + ^{2}F 7% + Other 26%<br>(^{8}S 11% + ^{6}I 8% + ^{6}D 7% + ^{4}D 11% + ^{4}H 10% + ^{4}F 9% + ^{4}G 6% + ^{2}G 6% + ^{2}F 7% + Other 24%)
11,854<br>(13,294)	3.8<br>(3.7)	^{8}S 12% + ^{6}I 6% + ^{6}D 10% + ^{4}D 12% + ^{4}H 8% + ^{4}F 8% + ^{2}G 6% + ^{2}F 7% + Other 31%<br>(^{8}S 12% + ^{6}P 6% + ^{6}I 7% + ^{6}D 10% + ^{4}D 13% + ^{4}H 8% + ^{4}F 8% + ^{2}G 6% + ^{2}F 8% + Other 22%)
13,747<br>(14,891)	4.0<br>(3.8)	^{8}S 15% + ^{6}P 8% + ^{6}D 15% + ^{6}F 5% + ^{4}D 15% + ^{4}F 7% + ^{2}F 6% + Other 27%<br>(^{8}S 14% + ^{6}P 10% + ^{6}D 14% + ^{4}D 17% + ^{4}F 8% + ^{2}F 7% + Other 29%)
14,042<br>(15,926)	3.2<br>(3.0)	^{6}P 23% + ^{6}D 24% + ^{6}F 8% + ^{4}D 10% + ^{4}F 7% + Other 27%<br>(^{6}P 29% + ^{6}D 26% + ^{6}F 7% + ^{4}D 10% + ^{4}F 6% + Other 20%)
14,723<br>(16,782)	4.2<br>(3.9)	^{6}I 28% + ^{6}D 9% + ^{4}H 19% + ^{6}H 7% + ^{4}G 7% + Other 30%<br>(^{6}P 11% + ^{6}I 26% + ^{6}D 12% + ^{4}H 16% + Other 36%)
16,018<br>(17,549)	3.6<br>(3.6)	^{6}P 30% + ^{6}I 9% + ^{6}D 22% + ^{4}D 11% + Other 28%<br>(^{6}P 28% + ^{6}I 12% + ^{6}D 22% + ^{4}D 7% + ^{4}H 5% + Other 25%)
16,289<br>(17,651)	3.8<br>(3.6)	^{8}S 8% + ^{6}P 10% + ^{6}I 29% + ^{6}D 10% + ^{4}D 6% + ^{4}H 18% + Other 19%<br>(^{8}S 7% + ^{6}P 10% + ^{6}I 33% + ^{6}D 9% + ^{4}D 6% + ^{4}H 19% + Other 15%)
16,507<br>(18,053)	4.4<br>(4.0)	^{6}I 33% + ^{6}D 8% + ^{6}F 7% + ^{4}H 21% + ^{2}G 6% + Other 25%<br>(^{6}I 41% + ^{6}D 7% + ^{4}H 25% + ^{2}G 7% + Other 21%)
16,584<br>(18,321)	4.5<br>(4.1)	^{6}P 16% + ^{6}I 21% + ^{6}D 13% + ^{6}F 7% + ^{4}H 8% + Other 35%<br>(^{6}P 18% + ^{6}I 25% + ^{6}D 12% + ^{4}H 11% + Other 33%)
17,675<br>(19,201)	5.6<br>(4.7)	^{6}P 5% + ^{6}I 36% + ^{6}D 6% + ^{4}H 12% + ^{4}K 7% + Other 34%<br>(^{6}P 13% + ^{6}I 34% + ^{6}D 8% + ^{4}H 14% + Other 31%)
17,690<br>(19,567)	6.5<br>(6.6)	^{6}I 36% + ^{6}D 7% + ^{6}F 8% + ^{4}H 7% + ^{4}K 11% + Other 31%<br>(^{6}I 48% + ^{4}H 9% + ^{4}K 11% + Other 32%)
17,850<br>(19,765)	4.9<br>(4.2)	^{6}I 41% + ^{6}D 12% + ^{6}F 6% + ^{4}H 14% + ^{6}H 7% + Other 20%<br>(^{6}P 21% + ^{6}I 26% + ^{6}D 13% + ^{4}H 11% + Other 28%)
18,288<br>(19,841)	5.8<br>(5.4)	^{6}I 39% + ^{6}D 11% + ^{4}H 8% + ^{6}H 9% + ^{4}K 5% + Other 27%<br>(^{6}I 31% + ^{6}D 21% + ^{6}F 11% + ^{4}H 7% + ^{4}F 5% + Other 25%)
18,490<br>(20,143)	4.6<br>(6.6)	^{6}P 23% + ^{6}I 24% + ^{6}D 11% + ^{4}H 9% + Other 33%<br>(^{6}I 47% + ^{6}D 5% + ^{6}H 6% + ^{4}H 8% + ^{4}K 11% + Other 24%)
18,640<br>(20,305)	7.4<br>(7.3)	^{6}I 46% + ^{4}H 6% + ^{4}K 18% + ^{2}L 5% + Other 25%<br>(^{6}I 52% + ^{4}H 7% + ^{4}K 17% + Other 23%)
18,707<br>(20,386)	7.7<br>(8.2)	^{6}I 50% + ^{6}H 6% + ^{4}K 18% + Other 26%<br>(^{6}I 57% + ^{4}K 22% + Other 21%)
18,836<br>(20,484)	7.1<br>(6.8)	^{6}I 46% + ^{4}H 7% + ^{4}K 16% + Other 31%<br>(^{6}I 52% + ^{4}H 7% + ^{4}K 13% + Other 27%)
19,275<br>(20,720)	6.8<br>(7.7)	^{6}P 6% + ^{6}I 44% + ^{4}H 7% + ^{4}K 14% + Other 30%<br>(^{6}I 54% + ^{4}K 19% + Other 27%)
19,418<br>(20,741)	7.8<br>(5.9)	^{6}I 45% + ^{4}K 21% + ^{2}L 6% + Other 28%<br>(^{6}I 58% + ^{4}H 15% + ^{4}K 7% + Other 21%)
19,562<br>(20,887)	5.4<br>(8.1)	^{6}I 51% + ^{4}H 18% + Other 31%<br>(^{6}I 54% + ^{4}K 23% + ^{2}L 6% + Other 18%)

19,782<br>(21,348)	5.7<br>(5.7)	^{6}I 47% + ^{4}H 14% + Other 39%<br>(^{6}I 56% + ^{4}H 15% + Other 29%)
20,182<br>(21,468)	7.3<br>(7.2)	^{6}I 57% + ^{4}K 16% + Other 26%<br>(^{6}I 61% + ^{4}H 5% + ^{4}K 15% + Other 19%)
20,354<br>(21,878)	6.4<br>(6.4)	^{6}I 50% + ^{4}H 5% + ^{6}H 7% + ^{4}K 8% + Other 30%<br>(^{6}I 60% + ^{6}H 5% + ^{4}H 7% + ^{4}K 8% + Other 20%)
20,508<br>(21,978)	6.8<br>(6.9)	^{6}I 57% + ^{4}H 5% + ^{6}H 5% + ^{4}K 12% + Other 21%<br>(^{6}I 59% + ^{6}H 5% + ^{4}K 11% + Other 25%)
20,824<br>(22,100)	6.0<br>(5.7)	^{6}I 54% + ^{4}H 12% + ^{4}K 6% + Other 29%<br>(^{6}I 62% + ^{4}H 14% + Other 24%)
21,005<br>(22,391)	6.8<br>(6.5)	^{6}I 47% + ^{4}H 7% + ^{4}K 11% + Other 35%<br>(^{6}I 61% + ^{4}H 9% + ^{4}K 8% + Other 22%)
21,019<br>(22,403)	6.0<br>(7.0)	^{6}I 42% + ^{6}D 6% + ^{6}F 7% + ^{4}K 9% + Other 36%<br>(^{6}I 62% + ^{4}K 13% + Other 25%)
21,096<br>(22,477)	6.2<br>(5.8)	^{6}I 51% + ^{4}H 8% + ^{4}K 7% + Other 33%<br>(^{6}I 44% + ^{6}D 12% + ^{6}F 6% + ^{4}K 7% + Other 31%)
21,132<br>(22,501)	6.9<br>(6.5)	^{6}I 54% + ^{4}H 6% + ^{4}K 13% + Other 27%<br>(^{6}I 56% + ^{4}H 9% + ^{4}K 8% + Other 27%)
21,210<br>(22,516)	6.3<br>(6.3)	^{6}I 54% + ^{4}H 9% + ^{4}K 7% + Other 30%<br>(^{6}I 58% + ^{4}H 10% + ^{4}K 7% + Other 25%)
21,346<br>(22,679)	6.3<br>(6.7)	^{6}I 43% + ^{6}D 13% + ^{6}F 6% + ^{4}K 9% + Other 29%<br>(^{6}I 55% + ^{6}D 7% + ^{4}K 11% + Other 26%)
21,597<br>(22,889)	6.5<br>(7.1)	^{6}P 6% + ^{6}I 52% + ^{4}K 11% + Other 31%<br>(^{6}I 67% + ^{4}K 12% + Other 20%)
21,646<br>(22,908)	6.6<br>(6.9)	^{6}I 56% + ^{4}K 11% + Other 33%<br>(^{6}I 60% + ^{4}K 13% + Other 27%)
22,258<br>(23,462)	5.5<br>(5.5)	^{6}I 32% + ^{6}D 26% + ^{6}F 7% + ^{4}K 6% + ^{4}F 10% + Other 19%<br>(^{6}I 35% + ^{6}D 29% + ^{6}F 8% + ^{4}F 8% + Other 20%)
22,765<br>(23,920)	5.1<br>(5.1)	^{6}I 33% + ^{6}D 31% + ^{4}F 13% + Other 23%<br>(^{6}I 31% + ^{6}D 35% + ^{6}F 6% + ^{4}F 12% + Other 16%)
22,852	5.0	^{6}I 30% + ^{6}D 23% + ^{4}D 6% + ^{6}H 5% + ^{2}F 8% + Other 28%
23,467	5.1	^{6}I 24% + ^{6}D 21% + ^{6}F 5% + ^{4}D 9% + ^{2}F 6% + Other 36%
23,538	4.9	^{6}I 21% + ^{6}D 26% + ^{4}D 9% + ^{2}F 7% + ^{2}O, ^{4}D 6% + Other 31%
23,902	4.4	^{6}I 17% + ^{6}D 19% + ^{6}H 5% + ^{6}F 7% + ^{4}D 9% + ^{4}F 5% + Other 38%

Table S26. Energy (cm^{-1}) and composition of the excited states less than **39,000 cm^{-1}** for **[Gd^{III}Cl$_6$]$^{3-}$**. The top text corresponds to LFDFT-PBE results and the text in parentheses to LFDFT-PBE0.

**E (cm^{-1})**	**J**	**$^{2S+1}$L Composition**
0<br>(0)	3.5<br>(3.5)	^{8}S 97% + Other 3%<br>(^{8}S 97% + Other 3%)
29,655<br>(29,922)	3.5<br>(3.5)	^{6}P 75% + ^{6}D 12% + ^{4}D 8% + Other 5%<br>(^{6}P 76% + ^{6}D 12% + ^{4}D 8% + Other 4%)
29,688<br>(30,482)	3.5<br>(2.5)	^{6}P 75% + ^{6}D 12% + ^{4}D 8% + Other 5%<br>(^{6}P 84% + ^{6}D 13% + Other 3%)
29,712<br>(31,011)	3.5<br>(1.5)	^{6}P 75% + ^{6}D 12% + ^{4}D 8% + Other 5%<br>(^{6}P 90% + ^{6}D 7% + Other 2%)

30,226<br>(32,848)	2.5<br>(3.5)	^{6}P 83% + ^{6}D 13% + Other 4%<br>(^{6}I 92% + ^{4}H 7% + Other 1%)
30,268<br>(32,853)	2.5<br>(3.5)	^{6}P 83% + ^{6}D 13% + Other 4%<br>(^{6}I 92% + ^{4}H 7% + Other 1%)
30,793<br>(33,214)	1.6<br>(4.5)	^{6}P 89% + ^{6}D 7% + Other 3%<br>(^{6}I 94% + Other 6%)
32,550<br>(33,220)	3.5<br>(4.5)	^{6}I 91% + ^{4}H 7% + Other 2%<br>(^{6}I 94% + Other 6%)
32,637<br>(33,430)	3.5<br>(8.5)	^{6}I 92% + ^{4}H 7% + Other 1%<br>(^{6}I 96% + Other 4%)
32,688<br>(33,517)	3.5<br>(5.5)	^{6}I 92% + ^{4}H 7% + Other 1%<br>(^{6}I 96% + Other 4%)
32,934<br>(33,524)	4.5<br>(5.5)	^{6}I 93% + Other 7%<br>(^{6}I 96% + Other 4%)
33,034<br>(33,703)	4.5<br>(6.6)	^{6}I 94% + Other 6%<br>(^{6}I 97% + Other 3%)
33,068<br>(33,712)	4.5<br>(7.5)	^{6}I 94% + Other 6%<br>(^{6}I 97% + Other 3%)
33,181<br>(33,718)	7.4<br>(7.5)	^{6}I 95% + Other 5%<br>(^{6}I 97% + Other 3%)
33,198<br>(37,300)	8.4<br>(4.5)	^{6}I 95% + Other 5%<br>(^{6}D 93% + Other 7%)
33,220<br>(37,314)	8.4<br>(4.5)	^{6}I 95% + Other 5%<br>(^{6}D 93% + Other 7%)
33,229<br>(38,119)	8.4<br>(0.5)	^{6}I 95% + Other 5%<br>(^{6}D 99% + Other 1%)
33,252<br>(38,234)	6.9<br>(3.5)	^{6}I 95% + Other 5%<br>(^{6}P 11% + ^{6}D 81% + Other 8%)
33,274<br>(38,356)	5.8<br>(1.5)	^{6}I 95% + Other 5%<br>(^{6}P 7% + ^{6}D 90% + Other 3%)
33,344<br>(38,485)	5.5<br>(2.5)	^{6}I 96% + Other 4%<br>(^{6}P 13% + ^{6}D 82% + Other 5%)
33,371<br>(38,497)	5.6<br>(2.5)	^{6}I 96% + Other 4%<br>(^{6}P 13% + ^{6}D 82% + Other 5%)
33,417	6.6	^{6}I 96% + Other 4%
33,453	7.1	^{6}I 96% + Other 4%
33,471	6.8	^{6}I 96% + Other 4%
33,496	7.5	^{6}I 97% + Other 3%
33,502	6.5	^{6}I 97% + Other 3%
33,529	7.4	^{6}I 97% + Other 3%

33,568	7.5	^{6}I 97% + Other 3%
36,886	4.5	^{6}D 91% + Other 9%
36,986	4.5	^{6}D 91% + Other 9%
37,154	4.5	^{6}D 92% + Other 8%
37,850	1.5	^{6}D 95% + Other 5%
37,923	3.0	^{6}P 10% + ^{6}D 82% + Other 8%
37,934	3.3	^{6}P 12% + ^{6}D 79% + Other 9%
38,038	3.2	^{6}P 9% + ^{6}D 84% + Other 7%
38,163	2.7	^{6}P 13% + ^{6}D 80% + Other 6%
38,185	2.3	^{6}P 8% + ^{6}D 87% + Other 4%
38,349	2.5	^{6}P 12% + ^{6}D 83% + Other 5%

Table S27. Energy (cm^{-1}) and composition of the excited states less than **24,000 cm^{-1}** for $[\mathbf{Tb^{IV}Cl_6}]^{2-}$. The top text corresponds to LFDFT-PBE results and the text in parentheses to LFDFT-PBE0.

**E (cm^{-1})**	**J**	**$^{2S+1}$L Composition**
1<br>(1)	3.5<br>(3.5)	^{8}S 94% + ^{6}P 6% + Other 0%<br>(^{8}S 94% + ^{6}P 6% + Other 0%)
16,957<br>(16,677)	3.7<br>(3.9)	^{6}P 50% + ^{6}D 17% + ^{4}D 12% + Other 20%<br>(^{6}P 51% + ^{6}I 11% + ^{6}D 13% + ^{4}D 12% + Other 13%)
17,146<br>(16,835)	3.7<br>(3.9)	^{6}P 54% + ^{6}D 18% + ^{4}D 12% + Other 17%<br>(^{6}P 55% + ^{6}I 9% + ^{6}D 14% + ^{4}D 12% + Other 11%)
17,291<br>(16,894)	3.8<br>(3.9)	^{6}P 56% + ^{6}D 17% + ^{4}D 12% + Other 14%<br>(^{6}P 55% + ^{6}I 9% + ^{6}D 14% + ^{4}D 12% + Other 10%)
17,630<br>(17,366)	3.0<br>(3.3)	^{6}P 62% + ^{6}D 21% + Other 16%<br>(^{6}P 62% + ^{6}I 15% + ^{6}D 15% + Other 8%)
17,903<br>(17,434)	2.9<br>(3.4)	^{6}P 68% + ^{6}I 5% + ^{6}D 18% + Other 9%<br>(^{6}P 63% + ^{6}I 16% + ^{6}D 13% + Other 7%)
18,416<br>(17,943)	2.3<br>(2.9)	^{6}P 74% + ^{6}I 6% + ^{6}D 12% + Other 8%<br>(^{6}P 69% + ^{6}I 17% + ^{6}D 8% + Other 5%)
18,882<br>(19,004)	3.6<br>(3.7)	^{6}P 6% + ^{6}I 73% + ^{4}H 13% + Other 9%<br>(^{6}I 79% + ^{4}H 13% + Other 8%)
19,159<br>(19,231)	4.2<br>(3.8)	^{6}I 77% + ^{4}H 11% + Other 12%<br>(^{6}I 81% + ^{4}H 12% + Other 7%)

19,503<br>(19,471)	4.6<br>(4.3)	^{6}I 82% + ^{4}H 9% + Other 10%<br>(^{6}I 80% + ^{4}H 11% + Other 9%)
19,594<br>(19,551)	4.4<br>(5.1)	^{6}I 80% + ^{4}H 10% + Other 10%<br>(^{6}I 84% + ^{4}H 8% + Other 8%)
19,668<br>(19,640)	5.0<br>(5.2)	^{6}I 80% + ^{4}H 8% + Other 12%<br>(^{6}I 87% + ^{4}H 8% + Other 6%)
19,714<br>(19,767)	6.2<br>(6.5)	^{6}I 81% + Other 19%<br>(^{6}I 86% + ^{4}H 5% + Other 9%)
19,739<br>(19,816)	6.8<br>(6.7)	^{6}I 79% + ^{6}D 6% + Other 15%<br>(^{6}I 88% + Other 12%)
19,847<br>(19,890)	8.0<br>(8.2)	^{6}I 86% + ^{4}K 6% + Other 8%<br>(^{6}I 90% + ^{4}K 7% + Other 3%)
19,990<br>(19,932)	6.7<br>(8.0)	^{6}I 83% + Other 17%<br>(^{6}I 90% + ^{4}K 7% + Other 3%)
20,033<br>(19,977)	5.8<br>(5.7)	^{6}I 87% + ^{4}H 6% + Other 7%<br>(^{6}I 90% + Other 10%)
20,132<br>(19,985)	7.9<br>(7.7)	^{6}I 86% + ^{4}K 6% + Other 7%<br>(^{6}I 90% + ^{4}K 6% + Other 4%)
20,165<br>(20,031)	6.2<br>(6.4)	^{6}I 86% + ^{4}H 5% + Other 9%<br>(^{6}I 89% + Other 11%)
20,212<br>(20,106)	7.4<br>(7.0)	^{6}I 89% + ^{4}K 6% + Other 6%<br>(^{6}I 92% + Other 8%)
20,270<br>(20,142)	7.6<br>(7.2)	^{6}I 88% + ^{4}K 6% + Other 7%<br>(^{6}I 85% + Other 15%)
20,351<br>(20,153)	6.8<br>(6.9)	^{6}I 85% + Other 15%<br>(^{6}I 91% + Other 9%)
20,402<br>(20,215)	5.9<br>(7.6)	^{6}I 89% + Other 11%<br>(^{6}I 80% + ^{6}D 6% + Other 14%)
20,413<br>(20,233)	7.1<br>(6.5)	^{6}I 90% + Other 10%<br>(^{6}I 87% + Other 13%)
20,490<br>(20,240)	5.9<br>(6.7)	^{6}I 91% + Other 9%<br>(^{6}I 92% + Other 8%)
20,620<br>(20,269)	7.3<br>(7.1)	^{6}I 91% + Other 9%<br>(^{6}I 90% + Other 10%)
20,626<br>(20,336)	7.4<br>(7.1)	^{6}I 91% + Other 9%<br>(^{6}I 90% + Other 10%)
20,652<br>(20,362)	7.0<br>(6.5)	^{6}I 91% + Other 9%<br>(^{6}I 92% + Other 8%)
20,663<br>(20,387)	6.6<br>(6.5)	^{6}I 91% + Other 9%<br>(^{6}I 91% + Other 9%)
20,706<br>(20,400)	7.3<br>(6.9)	^{6}I 93% + Other 7%<br>(^{6}I 84% + ^{6}D 6% + Other 10%)
20,720<br>(20,501)	6.4<br>(6.8)	^{6}I 91% + Other 9%<br>(^{6}I 83% + ^{6}D 6% + Other 10%)
21,874<br>(20,748)	4.8<br>(6.2)	^{6}I 17% + ^{6}D 65% + ^{6}F 8% + Other 10%<br>(^{6}P 10% + ^{6}I 76% + ^{6}D 7% + Other 7%)

22,161<br>(21,911)	4.8<br>(4.6)	^{6}I 12% + ^{6}D 71% + ^{6}F 7% + ^{4}F 6% + Other 5%<br>(^{6}D 80% + ^{6}F 8% + ^{4}F 6% + Other 6%)
22,469<br>(22,106)	4.6<br>(4.6)	^{6}I 7% + ^{6}D 75% + ^{4}F 7% + Other 11%<br>(^{6}D 81% + ^{4}F 7% + Other 11%)
23,005<br>(22,267)	3.3<br>(4.5)	^{6}P 20% + ^{6}I 6% + ^{6}D 55% + ^{6}F 9% + Other 10%<br>(^{6}D 84% + ^{4}F 7% + Other 9%)
23,031<br>(23,044)	3.4<br>(2.1)	^{6}P 14% + ^{6}D 65% + ^{6}F 6% + Other 14%<br>(^{6}D 85% + Other 15%)
23,098<br>(23,068)	3.0<br>(3.5)	^{6}P 6% + ^{6}D 78% + Other 16%<br>(^{6}P 17% + ^{6}D 62% + ^{6}F 6% + Other 16%)
23,437<br>(23,092)	2.7<br>(3.7)	^{6}P 7% + ^{6}D 80% + Other 13%<br>(^{6}P 16% + ^{6}D 63% + ^{6}F 5% + Other 16%)
23,500<br>(23,282)	3.2<br>(3.1)	^{6}P 17% + ^{6}D 66% + ^{6}F 5% + Other 11%<br>(^{6}P 10% + ^{6}D 75% + Other 14%)
23,689<br>(23,349)	2.6<br>(2.4)	^{6}P 10% + ^{6}D 78% + Other 12%<br>(^{6}P 18% + ^{6}D 69% + Other 13%)
23,868<br>(23,555)	2.9<br>(2.6)	^{6}P 15% + ^{6}D 72% + Other 12%<br>(^{6}P 16% + ^{6}D 72% + Other 11%)
(23,686)	(2.7)	(^{6}P 19% + ^{6}D 69% + Other 12%)

Table S28. Energy (cm^{-1}) and composition of the excited states less than **30,000 cm^{-1}** for [**Cm^{III}Cl$_6$**]$^{3-}$. The top text corresponds to LFDFT-PBE results and the text in parentheses to LFDFT-PBE0.

**E (cm^{-1})**	**J**	**$^{2S+1}$L Composition**
0<br>(0)	3.5<br>(3.5)	^{8}S 81% + ^{6}P 16% + Other 3%<br>(^{8}S 82% + ^{6}P 16% + Other 3%)
10<br>(9)	3.5<br>(3.5)	^{8}S 81% + ^{6}P 16% + Other 3%<br>(^{8}S 82% + ^{6}P 16% + Other 3%)
17<br>(18,250)	3.5<br>(3.5)	^{8}S 81% + ^{6}P 16% + Other 3%<br>(^{8}S 9% + ^{6}P 17% + ^{6}I 5% + ^{6}D 14% + ^{4}D 19% + ^{4}H 6% + ^{4}F 8% + ^{2}F 6% + Other 17%)
17,471<br>(18,536)	3.5<br>(3.5)	^{8}S 9% + ^{6}P 14% + ^{6}I 6% + ^{6}D 13% + ^{4}N 18% + ^{4}D 6% + ^{4}K 8% + ^{4}S 7% + Other 19%<br>(^{8}S 10% + ^{6}P 18% + ^{6}I 5% + ^{6}D 14% + ^{4}D 19% + ^{4}H 5% + ^{4}F 7% + ^{2}F 6% + Other 15%)
17,946<br>(18,874)	3.5<br>(3.5)	^{8}S 10% + ^{6}P 16% + ^{6}I 6% + ^{6}D 13% + ^{4}N 19% + ^{4}D 6% + ^{4}K 7% + ^{4}S 7% + Other 16%<br>(^{8}S 10% + ^{6}P 20% + ^{6}D 15% + ^{4}D 20% + ^{4}F 7% + ^{2}F 6% + Other 21%)
18,490<br>(21,300)	3.5<br>(2.5)	^{8}S 11% + ^{6}P 19% + ^{6}D 15% + ^{4}N 21% + ^{4}K 7% + ^{4}S 6% + Other 21%<br>(^{6}P 48% + ^{6}D 29% + ^{4}D 11% + Other 12%)
20,695<br>(21,558)	2.6<br>(2.6)	^{6}P 45% + ^{6}D 29% + ^{4}N 11% + Other 14%<br>(^{6}P 49% + ^{6}D 28% + ^{4}D 11% + Other 12%)
21,100<br>(22,369)	2.7<br>(3.5)	^{6}P 46% + ^{6}D 27% + ^{4}N 11% + Other 16%<br>(^{6}P 9% + ^{6}I 47% + ^{6}D 6% + ^{4}H 22% + Other 16%)
21,805<br>(22,542)	3.5<br>(3.5)	^{6}P 9% + ^{6}I 45% + ^{6}D 7% + ^{4}D 22% + Other 17%<br>(^{6}P 9% + ^{6}I 47% + ^{6}D 7% + ^{4}H 22% + Other 15%)
22,050<br>(22,569)	3.5<br>(3.5)	^{6}P 10% + ^{6}I 43% + ^{6}D 9% + ^{4}D 21% + Other 17%<br>(^{6}P 6% + ^{6}I 51% + ^{4}H 24% + Other 18%)
22,110<br>(23,406)	3.5<br>(1.9)	^{6}P 5% + ^{6}I 51% + ^{4}D 25% + Other 19%<br>(^{6}P 60% + ^{6}D 21% + ^{4}S 7% + Other 13%)
22,902<br>(23,739)	2.6<br>(4.4)	^{6}P 47% + ^{6}I 14% + ^{6}D 17% + ^{4}G 5% + Other 16%<br>(^{6}I 54% + ^{4}H 19% + Other 26%)

23,152<br>(24,032)	4.1<br>(4.5)	^{6}P 11% + ^{6}I 44% + ^{6}D 8% + ^{4}D 17% + Other 20%<br>(^{6}I 56% + ^{6}D 5% + ^{4}H 20% + Other 19%)
23,599<br>(24,186)	4.4<br>(4.5)	^{6}I 52% + ^{6}D 6% + ^{4}D 19% + Other 22%<br>(^{6}I 58% + ^{4}H 20% + Other 22%)
23,839<br>(25,083)	4.6<br>(8.4)	^{6}I 56% + ^{6}D 6% + ^{4}D 20% + Other 18%<br>(^{6}I 71% + ^{4}K 20% + Other 8%)
24,525<br>(25,114)	7.7<br>(8.5)	^{6}I 69% + ^{6}H 16% + Other 16%<br>(^{6}I 72% + ^{4}K 20% + Other 8%)
24,571<br>(25,156)	8.2<br>(8.4)	^{6}I 69% + ^{6}H 20% + Other 11%<br>(^{6}I 72% + ^{4}K 20% + Other 8%)
24,609<br>(25,175)	8.3<br>(8.5)	^{6}I 69% + ^{6}H 21% + Other 10%<br>(^{6}I 72% + ^{4}K 21% + Other 7%)
24,626<br>(25,490)	8.5<br>(5.9)	^{6}I 70% + ^{6}H 21% + Other 8%<br>(^{6}I 74% + ^{4}H 12% + Other 14%)
24,697<br>(25,541)	8.3<br>(5.7)	^{6}I 70% + ^{6}H 20% + Other 10%<br>(^{6}I 73% + ^{4}H 12% + Other 14%)
24,740<br>(25,705)	8.4<br>(5.5)	^{6}I 71% + ^{6}H 21% + Other 8%<br>(^{6}I 75% + ^{4}H 13% + Other 12%)
24,987<br>(25,811)	6.5<br>(5.5)	^{6}I 72% + ^{4}D 9% + ^{6}H 8% + Other 11%<br>(^{6}I 75% + ^{4}H 13% + Other 12%)
25,026<br>(26,228)	5.9<br>(6.4)	^{6}I 70% + ^{4}D 11% + Other 18%<br>(^{6}I 74% + ^{4}H 6% + Other 20%)
25,291<br>(26,324)	5.6<br>(6.5)	^{6}I 73% + ^{4}D 14% + Other 13%<br>(^{6}I 77% + ^{4}H 6% + ^{4}K 5% + Other 11%)
25,468<br>(26,400)	5.5<br>(6.5)	^{6}I 74% + ^{4}D 13% + Other 13%<br>(^{6}I 78% + ^{4}H 7% + ^{4}K 5% + Other 10%)
25,610<br>(26,476)	6.2<br>(7.4)	^{6}I 66% + ^{6}D 8% + ^{4}D 6% + Other 20%<br>(^{6}I 79% + ^{4}K 11% + Other 10%)
25,827<br>(26,518)	6.5<br>(6.5)	^{6}I 74% + ^{4}D 6% + ^{6}H 6% + Other 14%<br>(^{6}I 78% + ^{4}H 7% + ^{4}K 5% + Other 10%)
25,972<br>(26,534)	7.2<br>(6.5)	^{6}I 77% + ^{6}H 10% + Other 13%<br>(^{6}I 78% + ^{4}H 7% + ^{4}K 5% + Other 10%)
25,983<br>(26,543)	6.5<br>(7.4)	^{6}I 77% + ^{4}D 7% + ^{6}H 6% + Other 11%<br>(^{6}I 80% + ^{4}K 11% + Other 9%)
26,084<br>(26,650)	7.4<br>(7.5)	^{6}I 77% + ^{6}H 11% + Other 12%<br>(^{6}I 82% + ^{4}K 11% + Other 7%)
26,150<br>(26,744)	6.5<br>(7.5)	^{6}I 76% + ^{4}D 7% + ^{6}H 6% + Other 11%<br>(^{6}I 82% + ^{4}K 11% + Other 7%)
26,165<br>(27,077)	6.5<br>(4.7)	^{6}I 76% + ^{4}D 7% + ^{6}H 6% + Other 11%<br>(^{6}I 18% + ^{6}D 48% + ^{6}F 13% + ^{4}F 10% + Other 11%)
26,271<br>(27,341)	7.5<br>(4.7)	^{6}I 81% + ^{6}H 12% + Other 8%<br>(^{6}I 18% + ^{6}D 50% + ^{6}F 12% + ^{4}F 10% + Other 10%)
26,379<br>(27,617)	7.4<br>(4.6)	^{6}I 79% + ^{6}H 12% + Other 9%<br>(^{6}I 17% + ^{6}D 52% + ^{6}F 10% + ^{4}F 12% + Other 10%)
26,395<br>(29,651)	7.4<br>(3.6)	^{6}I 80% + ^{6}H 12% + Other 8%<br>(^{6}P 11% + ^{6}D 37% + ^{6}G 5% + ^{6}F 13% + ^{4}D 19% + Other 15%)
26,504<br>(29,676)	5.0<br>(3.5)	^{6}I 27% + ^{6}D 39% + ^{6}F 11% + ^{4}K 8% + Other 14%<br>(^{6}P 10% + ^{6}D 40% + ^{6}F 12% + ^{4}D 19% + ^{2}F 5% + Other 13%)
26,917	4.9	^{6}I 23% + ^{6}D 45% + ^{6}F 10% + ^{4}K 10% + Other 12%
27,294	4.7	^{6}I 19% + ^{6}D 49% + ^{6}F 9% + ^{4}K 12% + Other 11%

29,145	3.5	^{6}P 8% + ^{6}D 40% + ^{6}F 12% + ^{4}N 20% + ^{4}S 7% + Other 12%
29,153	3.7	^{6}P 11% + ^{6}D 35% + ^{6}G 6% + ^{6}F 12% + ^{4}N 18% + Other 18%
29,177	3.6	^{6}P 10% + ^{6}D 38% + ^{6}F 12% + ^{4}N 19% + ^{4}S 5% + Other 16%

Table S29. Energy (cm^{-1}) and composition of the excited states less than **19,000 cm^{-1}** for [$\mathbf{Bk^{IV}Cl_6}$]$^{2-}$. The top text corresponds to LFDFT-PBE results and the text in parentheses to LFDFT-PBE0.

E (cm^{-1})	J	$^{2S+1}$L Composition
0<br>(0)	3.5<br>(3.5)	^{8}S 58% + ^{6}P 26% + ^{4}D 7% + Other 9%<br>(^{8}S 64% + ^{6}P 25% + ^{4}D 6% + Other 5%)
156<br>(60)	3.5<br>(3.5)	^{8}S 62% + ^{6}P 26% + ^{4}D 6% + Other 6%<br>(^{8}S 65% + ^{6}P 25% + ^{4}D 5% + Other 5%)
247<br>(106)	3.5<br>(3.5)	^{8}S 64% + ^{6}P 26% + ^{4}D 5% + Other 5%<br>(^{8}S 66% + ^{6}P 25% + ^{4}D 5% + Other 4%)
7,060<br>(8,525)	3.7<br>(3.6)	^{8}S 16% + ^{6}I 7% + ^{4}D 7% + ^{4}H 14% + ^{4}F 8% + ^{4}G 7% + ^{2}G 8% + ^{2}F 7% + Other 25%<br>(^{8}S 14% + ^{6}I 7% + ^{6}D 5% + ^{4}D 10% + ^{4}H 11% + ^{4}F 9% + ^{4}G 6% + ^{2}G 8% + ^{2}F 8% + Other 20%)
8,699<br>(9,270)	3.6<br>(3.5)	^{8}S 15% + ^{6}I 7% + ^{6}D 6% + ^{4}D 10% + ^{4}H 12% + ^{4}F 8% + ^{4}G 6% + ^{2}G 8% + ^{2}F 9% + Other 20%<br>(^{8}S 14% + ^{6}I 7% + ^{6}D 6% + ^{4}D 11% + ^{4}H 11% + ^{4}F 8% + ^{4}G 5% + ^{2}G 8% + ^{2}F 9% + Other 19%)
10,270<br>(10,274)	3.8<br>(3.6)	^{8}S 17% + ^{6}D 10% + ^{4}D 14% + ^{4}H 7% + ^{4}F 8% + ^{2}G 6% + ^{2}F 9% + Other 29%<br>(^{8}S 15% + ^{6}I 6% + ^{6}D 8% + ^{4}D 14% + ^{4}H 9% + ^{4}F 9% + ^{2}G 7% + ^{2}F 9% + Other 24%)
11,061<br>(12,324)	3.0<br>(2.7)	^{6}P 23% + ^{6}D 24% + ^{6}F 8% + ^{4}D 11% + ^{4}F 8% + Other 26%<br>(^{6}P 30% + ^{6}D 27% + ^{6}F 7% + ^{4}D 12% + ^{4}F 7% + Other 17%)
11,562<br>(12,942)	4.2<br>(3.4)	^{6}I 25% + ^{6}D 10% + ^{4}H 21% + ^{6}H 6% + ^{4}F 6% + ^{4}G 8% + Other 25%<br>(^{6}P 21% + ^{6}I 14% + ^{6}D 18% + ^{4}D 9% + ^{4}H 9% + ^{4}F 6% + Other 23%)
12,642<br>(13,403)	3.4<br>(3.7)	^{6}P 29% + ^{6}I 6% + ^{6}D 23% + ^{6}F 5% + ^{4}D 12% + Other 25%<br>(^{6}P 16% + ^{6}I 17% + ^{6}D 19% + ^{6}F 5% + ^{4}H 12% + ^{4}F 5% + Other 25%)
12,807<br>(13,522)	4.4<br>(3.6)	^{6}I 30% + ^{6}D 10% + ^{6}F 7% + ^{4}H 22% + ^{2}G 6% + Other 24%<br>(^{8}S 9% + ^{6}P 7% + ^{6}I 30% + ^{6}D 11% + ^{4}D 6% + ^{4}H 21% + Other 16%)
12,927<br>(13,558)	3.7<br>(3.8)	^{8}S 10% + ^{6}P 8% + ^{6}I 27% + ^{6}D 11% + ^{4}D 6% + ^{4}H 20% + Other 19%<br>(^{6}I 34% + ^{6}D 10% + ^{4}H 24% + ^{2}G 5% + Other 27%)
13,077<br>(13,943)	4.4<br>(4.2)	^{6}P 11% + ^{6}I 21% + ^{6}D 12% + ^{6}F 7% + ^{4}H 13% + ^{4}F 5% + ^{4}G 6% + Other 26%<br>(^{6}P 7% + ^{6}I 29% + ^{6}D 10% + ^{4}H 19% + ^{4}F 6% + ^{4}G 6% + Other 24%)
13,894<br>(14,489)	5.6<br>(4.5)	^{6}I 33% + ^{6}D 5% + ^{4}H 15% + ^{4}K 7% + ^{4}G 6% + Other 33%<br>(^{6}I 32% + ^{6}D 9% + ^{4}H 19% + ^{4}F 6% + ^{4}G 5% + Other 29%)
13,953<br>(14,864)	6.7<br>(5.0)	^{6}I 34% + ^{6}F 6% + ^{4}H 8% + ^{4}K 13% + ^{4}G 6% + Other 32%<br>(^{6}I 30% + ^{6}D 12% + ^{6}F 5% + ^{4}H 17% + ^{4}F 8% + ^{4}G 6% + Other 21%)
14,344<br>(14,959)	5.1<br>(2.7)	^{6}I 31% + ^{6}D 14% + ^{6}F 9% + ^{4}H 12% + Other 34%<br>(^{6}P 39% + ^{6}I 10% + ^{6}D 18% + ^{4}H 5% + ^{4}S 8% + Other 19%)
14,483<br>(15,261)	5.2<br>(6.9)	^{6}P 12% + ^{6}I 26% + ^{6}D 10% + ^{4}H 11% + ^{4}K 5% + Other 36%<br>(^{6}I 44% + ^{4}H 10% + ^{4}K 15% + Other 31%)
14,539<br>(15,363)	5.8<br>(7.9)	^{6}P 16% + ^{6}I 27% + ^{6}D 9% + ^{4}H 7% + ^{4}K 10% + Other 31%<br>(^{6}I 45% + ^{4}K 24% + ^{2}L 7% + Other 24%)
14,592<br>(15,422)	7.4<br>(8.0)	^{6}I 38% + ^{4}H 7% + ^{4}K 19% + ^{2}L 7% + Other 30%<br>(^{6}I 46% + ^{4}K 25% + ^{2}L 8% + Other 21%)
14,748<br>(15,545)	7.4<br>(8.3)	^{6}I 39% + ^{4}K 19% + ^{2}L 6% + Other 36%<br>(^{6}I 49% + ^{4}K 27% + ^{2}L 8% + Other 16%)
14,831<br>(15,637)	7.1<br>(7.8)	^{6}I 42% + ^{4}H 7% + ^{4}K 17% + ^{2}L 5% + Other 29%<br>(^{6}I 46% + ^{4}K 23% + ^{2}L 7% + Other 24%)

15,143<br>(15,749)	7.8<br>(8.2)	^{6}I 39% + ^{4}K 23% + ^{2}L 8% + Other 31%<br>(^{6}I 49% + ^{4}K 26% + ^{2}L 8% + Other 18%)
15,180<br>(16,027)	7.1<br>(6.5)	^{6}I 45% + ^{4}H 6% + ^{4}K 17% + Other 33%<br>(^{6}I 52% + ^{4}H 10% + ^{4}K 11% + Other 27%)
15,305<br>(16,052)	5.8<br>(6.7)	^{6}I 48% + ^{4}H 17% + ^{4}K 7% + Other 28%<br>(^{6}I 52% + ^{4}H 9% + ^{4}K 13% + Other 27%)
15,651<br>(16,329)	5.9<br>(5.8)	^{6}I 44% + ^{4}H 15% + ^{4}K 6% + Other 35%<br>(^{6}I 52% + ^{4}H 16% + ^{4}K 5% + Other 27%)
15,937<br>(16,428)	7.1<br>(6.2)	^{6}I 52% + ^{4}H 7% + ^{4}K 16% + Other 25%<br>(^{6}I 48% + ^{6}D 8% + ^{6}F 6% + ^{4}H 9% + ^{4}K 8% + Other 22%)
16,152<br>(16,702)	6.3<br>(5.4)	^{6}I 47% + ^{4}H 8% + ^{6}H 7% + ^{4}K 8% + Other 31%<br>(^{6}I 52% + ^{4}H 17% + Other 31%)
16,297<br>(16,846)	6.8<br>(6.4)	^{6}I 53% + ^{4}H 8% + ^{6}H 5% + ^{4}K 12% + Other 22%<br>(^{6}I 54% + ^{4}H 10% + ^{4}K 8% + Other 29%)
16,458<br>(17,046)	5.9<br>(6.5)	^{6}I 46% + ^{4}H 14% + ^{4}K 6% + Other 34%<br>(^{6}I 54% + ^{4}H 11% + ^{4}K 9% + ^{4}I 5% + Other 22%)
16,483<br>(17,075)	6.7<br>(6.9)	^{6}I 41% + ^{4}H 12% + ^{4}K 11% + ^{4}I 6% + Other 30%<br>(^{6}I 56% + ^{4}K 12% + Other 31%)
16,639<br>(17,152)	6.8<br>(6.6)	^{6}I 47% + ^{4}H 10% + ^{4}K 13% + ^{4}I 5% + Other 25%<br>(^{6}I 53% + ^{4}H 10% + ^{4}K 9% + ^{4}I 5% + Other 22%)
16,714<br>(17,253)	6.3<br>(5.5)	^{6}I 48% + ^{4}H 12% + ^{4}K 8% + Other 32%<br>(^{6}I 42% + ^{6}D 14% + ^{6}F 7% + ^{4}H 8% + Other 29%)
16,754<br>(17,270)	5.7<br>(6.9)	^{6}I 34% + ^{6}D 7% + ^{6}F 6% + ^{4}D 6% + ^{4}H 5% + ^{4}K 8% + Other 34%<br>(^{6}I 57% + ^{6}D 5% + ^{4}K 12% + Other 25%)
16,801<br>(17,558)	6.5<br>(6.8)	^{6}I 49% + ^{6}D 5% + ^{4}H 9% + ^{4}K 10% + Other 27%<br>(^{6}I 52% + ^{4}K 14% + Other 33%)
16,894<br>(17,627)	6.1<br>(7.3)	^{6}I 41% + ^{6}D 9% + ^{6}F 6% + ^{4}H 5% + ^{4}K 9% + Other 29%<br>(^{6}I 64% + ^{4}K 16% + Other 20%)
17,173<br>(17,663)	6.3<br>(7.0)	^{6}P 5% + ^{6}I 43% + ^{4}K 12% + Other 40%<br>(^{6}I 56% + ^{4}K 15% + Other 29%)
17,203<br>(17,848)	6.6<br>(5.5)	^{6}I 49% + ^{4}K 12% + Other 39%<br>(^{6}I 37% + ^{6}D 23% + ^{6}F 9% + ^{4}K 5% + ^{4}F 6% + Other 20%)
17,713<br>(18,192)	5.5<br>(5.1)	^{6}I 35% + ^{6}D 22% + ^{6}F 8% + ^{4}K 6% + ^{4}F 8% + Other 22%<br>(^{6}I 31% + ^{6}D 29% + ^{6}F 9% + ^{4}F 9% + Other 21%)
18,164<br>(18,807)	5.2<br>(3.9)	^{6}I 33% + ^{6}D 28% + ^{4}F 12% + Other 26%<br>(^{6}I 8% + ^{6}D 25% + ^{6}F 11% + ^{4}D 21% + ^{2}F 11% + Other 24%)
18,242<br>(18,908)	4.9<br>(4.8)	^{6}I 26% + ^{6}D 21% + ^{6}F 6% + ^{4}D 9% + ^{2}F 9% + Other 30%<br>(^{6}P 5% + ^{6}I 18% + ^{6}D 14% + ^{6}G 7% + ^{6}F 8% + ^{4}D 12% + ^{2}F 6% + Other 30%)
18,623<br>(18,954)	5.3<br>(4.5)	^{6}I 25% + ^{6}D 17% + ^{4}D 8% + ^{4}K 7% + ^{2}F 6% + Other 37%<br>(^{6}P 5% + ^{6}I 14% + ^{6}D 20% + ^{6}G 6% + ^{6}F 9% + ^{4}D 17% + ^{2}F 7% + Other 21%)
18,639	5.5	^{6}I 28% + ^{6}D 14% + ^{4}D 8% + ^{4}K 8% + ^{2}F 6% + Other 36%

Table S30. Energy (cm^{-1}) and composition of the excited states less than **37,000 cm^{-1}** for **$[Gd^{III}Br_6]^{3-}$**. The top text corresponds to LFDFT-PBE results and the text in parentheses to LFDFT-PBE0.

**E (cm^{-1})**	**J**	**$^{2S+1}$L Composition**
0<br>(0)	3.5<br>(3.5)	^{8}S 97% + Other 3%<br>(^{8}S 97% + Other 3%)

28,420<br>(28,466)	3.5<br>(3.5)	^{6}P 74% + ^{6}D 13% + ^{4}D 8% + Other 5%<br>(^{6}P 75% + ^{6}D 12% + ^{4}D 8% + Other 5%)
28,453<br>(28,476)	3.5<br>(3.5)	^{6}P 74% + ^{6}D 12% + ^{4}D 8% + Other 5%<br>(^{6}P 75% + ^{6}D 12% + ^{4}D 8% + Other 5%)
28,478<br>(28,481)	3.5<br>(3.5)	^{6}P 75% + ^{6}D 12% + ^{4}D 8% + Other 5%<br>(^{6}P 75% + ^{6}D 12% + ^{4}D 8% + Other 5%)
28,989<br>(29,025)	2.5<br>(2.6)	^{6}P 82% + ^{6}D 14% + Other 4%<br>(^{6}P 83% + ^{6}D 13% + Other 4%)
29,031<br>(29,032)	2.5<br>(2.6)	^{6}P 83% + ^{6}D 13% + Other 4%<br>(^{6}P 83% + ^{6}D 13% + Other 4%)
29,552<br>(29,551)	1.6<br>(1.6)	^{6}P 89% + ^{6}D 8% + Other 3%<br>(^{6}P 89% + ^{6}D 7% + Other 3%)
31,218<br>(31,294)	3.5<br>(3.5)	^{6}I 91% + ^{4}H 7% + Other 2%<br>(^{6}I 92% + ^{4}H 7% + Other 1%)
31,305<br>(31,316)	3.5<br>(3.5)	^{6}I 91% + ^{4}H 7% + Other 1%<br>(^{6}I 92% + ^{4}H 7% + Other 1%)
31,356<br>(31,334)	3.5<br>(3.5)	^{6}I 92% + ^{4}H 7% + Other 1%<br>(^{6}I 91% + ^{4}H 7% + Other 1%)
31,603<br>(31,662)	4.5<br>(4.5)	^{6}I 93% + ^{4}H 5% + Other 2%<br>(^{6}I 94% + ^{4}H 5% + Other 1%)
31,701<br>(31,676)	4.5<br>(4.5)	^{6}I 94% + ^{4}H 5% + Other 1%<br>(^{6}I 94% + ^{4}H 5% + Other 1%)
31,736<br>(31,693)	4.5<br>(4.5)	^{6}I 94% + ^{4}H 5% + Other 1%<br>(^{6}I 94% + ^{4}H 5% + Other 1%)
31,850<br>(31,885)	7.4<br>(8.5)	^{6}I 95% + Other 5%<br>(^{6}I 96% + Other 4%)
31,865<br>(31,897)	8.4<br>(8.5)	^{6}I 95% + Other 5%<br>(^{6}I 95% + Other 5%)
31,887<br>(31,966)	8.4<br>(5.5)	^{6}I 95% + Other 5%<br>(^{6}I 96% + Other 4%)
31,894<br>(31,976)	8.4<br>(5.5)	^{6}I 95% + Other 5%<br>(^{6}I 96% + Other 4%)
31,919<br>(31,993)	6.9<br>(5.5)	^{6}I 95% + Other 5%<br>(^{6}I 96% + Other 4%)
31,942<br>(32,147)	5.8<br>(6.5)	^{6}I 95% + Other 5%<br>(^{6}I 97% + Other 3%)
32,010<br>(32,157)	5.5<br>(7.5)	^{6}I 96% + Other 4%<br>(^{6}I 97% + Other 3%)
32,037<br>(32,166)	5.6<br>(6.8)	^{6}I 96% + Other 4%<br>(^{6}I 97% + Other 3%)
32,084<br>(32,178)	6.6<br>(7.1)	^{6}I 96% + Other 4%<br>(^{6}I 97% + Other 3%)
32,120<br>(32,183)	7.1<br>(7.4)	^{6}I 96% + Other 4%<br>(^{6}I 97% + Other 3%)
32,136<br>(32,194)	6.8<br>(7.3)	^{6}I 96% + Other 4%<br>(^{6}I 96% + Other 4%)

32,162<br>(35,547)	7.5<br>(4.5)	^{6}I 97% + Other 3%<br>(^{6}D 92% + Other 8%)
32,168<br>(35,582)	6.5<br>(4.5)	^{6}I 97% + Other 3%<br>(^{6}D 92% + Other 8%)
32,194<br>(35,609)	7.4<br>(4.5)	^{6}I 97% + Other 3%<br>(^{6}D 92% + Other 8%)
32,234	7.5	^{6}I 97% + Other 3%
35,395	4.5	^{6}D 90% + Other 10%
35,493	4.5	^{6}D 91% + Other 9%
35,658	4.5	^{6}D 92% + Other 8%
36,353	1.5	^{6}D 95% + Other 5%
36,422	3.1	^{6}P 11% + ^{6}D 81% + Other 8%
36,433	3.3	^{6}P 13% + ^{6}D 78% + Other 9%
36,534	3.2	^{6}P 9% + ^{6}D 84% + Other 7%
36,661	2.7	^{6}P 14% + ^{6}D 80% + Other 7%
36,682	2.3	^{6}P 8% + ^{6}D 87% + Other 4%
36,844	2.5	^{6}P 13% + ^{6}D 82% + Other 5%

Table S31. Energy (cm^{-1}) and composition of the excited states less than **22,000 cm^{-1}** for [**Tb^{IV}Br$_6$**]$^{2-}$. The top text corresponds to LFDFT-PBE results and the text in parentheses to LFDFT-PBE0.

**E (cm^{-1})**	**J**	**$^{2S+1}$L Composition**
1<br>(2)	3.5<br>(3.5)	^{8}S 94% + ^{6}P 6% + ^{4}F% + Other 0%<br>(^{8}S 93% + ^{6}P 6% + Other 0%)
15,708<br>(14,273)	3.6<br>(4.3)	^{6}P 48% + ^{6}D 17% + ^{4}D 13% + 0% + Other 22%<br>(^{6}P 41% + ^{6}I 18% + ^{6}D 10% + ^{4}D 12% + Other 19%)
15,905<br>(14,580)	3.7<br>(4.4)	^{6}P 52% + ^{6}D 18% + ^{4}D 12% + 0% + Other 17%<br>(^{6}P 47% + ^{6}I 17% + ^{6}D 11% + ^{4}D 11% + Other 14%)
16,054<br>(14,700)	3.7<br>(4.4)	^{6}P 55% + ^{6}D 18% + ^{4}D 12% + 0% + Other 15%<br>(^{6}P 48% + ^{6}I 18% + ^{6}D 10% + ^{4}D 11% + Other 13%)
16,384<br>(15,030)	2.9<br>(3.8)	^{6}P 61% + ^{6}D 22% + 0% + Other 17%<br>(^{6}P 54% + ^{6}I 24% + ^{6}D 12% + Other 11%)
16,662<br>(15,145)	2.9<br>(3.9)	^{6}P 67% + ^{6}D 18% + 0% + Other 14%<br>(^{6}P 56% + ^{6}I 26% + ^{6}D 10% + Other 9%)
17,165<br>(15,600)	2.3<br>(3.5)	^{6}P 73% + ^{6}I 6% + ^{6}D 12% + 0% + Other 9%<br>(^{6}P 61% + ^{6}I 26% + ^{6}D 6% + Other 6%)
17,549<br>(16,791)	3.6<br>(4.0)	^{6}P 6% + ^{6}I 72% + ^{4}H 13% + 0% + Other 9%<br>(^{6}I 72% + ^{4}H 14% + Other 14%)

17,814<br>(17,152)	4.1<br>(4.8)	^{6}I 76% + ^{4}H 11% + 0% + Other 13%<br>(^{6}I 76% + ^{4}H 10% + Other 14%)
18,159<br>(17,305)	4.5<br>(6.1)	^{6}I 81% + ^{4}H 10% + 0% + Other 9%<br>(^{6}I 78% + ^{6}F 6% + Other 16%)
18,242<br>(17,385)	4.3<br>(6.1)	^{6}I 78% + ^{4}H 11% + 0% + Other 11%<br>(^{6}I 82% + ^{4}H 6% + Other 13%)
18,313<br>(17,661)	5.0<br>(5.4)	0% + Other 100%<br>(^{6}I 83% + ^{4}H 8% + Other 9%)
18,371<br>(17,787)	6.2<br>(6.1)	^{6}I 78% + ^{4}H 8% + 0% + Other 14%<br>(^{6}I 84% + ^{4}H 6% + Other 10%)
18,397<br>(17,889)	6.9<br>(7.0)	^{6}I 81% + 0% + ^{6}H 5% + Other 14%<br>(^{6}I 82% + Other 18%)
18,502<br>(17,940)	8.0<br>(7.3)	^{6}I 79% + ^{6}D 6% + 0% + Other 15%<br>(^{6}I 85% + ^{4}K 6% + Other 10%)
18,621<br>(17,946)	6.7<br>(7.1)	^{6}I 85% + ^{4}K 7% + 0% + Other 8%<br>(^{6}I 84% + Other 16%)
18,677<br>(18,004)	5.8<br>(7.2)	^{6}I 82% + 0% + Other 18%<br>(^{6}I 87% + ^{4}K 5% + Other 8%)
18,759<br>(18,008)	7.8<br>(6.1)	^{6}I 86% + ^{4}H 7% + 0% + Other 7%<br>(^{6}I 84% + ^{4}H 6% + Other 9%)
18,766<br>(18,065)	7.9<br>(7.6)	^{6}I 85% + ^{4}K 6% + 0% + Other 9%<br>(^{6}I 86% + ^{4}K 6% + Other 9%)
18,794<br>(18,106)	6.3<br>(6.8)	^{6}I 86% + ^{4}K 7% + 0% + Other 7%<br>(^{6}I 82% + Other 18%)
18,842<br>(18,146)	7.5<br>(6.8)	^{6}I 86% + ^{4}H 5% + 0% + Other 9%<br>(^{6}I 83% + Other 17%)
18,897<br>(18,254)	7.6<br>(6.6)	^{6}I 88% + ^{4}K 6% + 0% + Other 6%<br>(^{6}I 81% + ^{6}D 7% + Other 12%)
18,973<br>(18,321)	6.8<br>(6.4)	^{6}I 88% + ^{4}K 6% + 0% + Other 7%<br>(^{6}I 84% + Other 16%)
19,029<br>(18,462)	5.9<br>(6.6)	^{6}I 85% + 0% + Other 15%<br>(^{6}I 89% + Other 11%)
19,039<br>(18,542)	7.1<br>(6.6)	^{6}I 89% + ^{4}H 5% + 0% + Other 6%<br>(^{6}I 91% + Other 9%)
19,120<br>(18,594)	5.8<br>(6.2)	^{6}I 89% + 0% + Other 11%<br>(^{6}I 89% + Other 11%)
19,240<br>(18,613)	7.3<br>(6.5)	^{6}I 91% + 0% + Other 9%<br>(^{6}I 92% + Other 8%)
19,258<br>(18,753)	7.4<br>(6.0)	^{6}I 91% + 0% + Other 9%<br>(^{6}I 63% + ^{6}D 18% + Other 18%)
19,275<br>(18,764)	7.0<br>(6.4)	^{6}I 91% + 0% + Other 9%<br>(^{6}P 6% + ^{6}I 64% + ^{6}D 17% + Other 14%)
19,291<br>(18,857)	6.6<br>(6.4)	^{6}I 90% + 0% + Other 10%<br>(^{6}P 11% + ^{6}I 58% + ^{6}D 18% + ^{4}K 5% + Other 8%)
19,332<br>(18,872)	7.3<br>(6.2)	^{6}I 91% + 0% + Other 9%<br>(^{6}P 5% + ^{6}I 66% + ^{6}D 17% + Other 12%)

19,340<br>(18,996)	6.4<br>(6.4)	^{6}I 92% + 0% + Other 8%<br>(^{6}I 66% + ^{6}D 19% + Other 15%)
20,381<br>(19,435)	4.8<br>(5.3)	^{6}I 91% + 0% + Other 9%<br>(^{6}P 10% + ^{6}I 56% + ^{6}D 24% + Other 10%)
20,654<br>(19,749)	4.8<br>(4.8)	^{6}I 17% + ^{6}D 63% + ^{6}F 8% + 0% + Other 11%<br>(^{6}I 10% + ^{6}D 70% + ^{6}F 7% + ^{4}F 7% + Other 6%)
20,949<br>(20,021)	4.6<br>(4.8)	^{6}I 12% + ^{6}D 70% + ^{6}F 7% + 0% + Other 6%<br>(^{6}I 9% + ^{6}D 71% + ^{4}F 8% + Other 12%)
21,481<br>(20,158)	3.4<br>(4.5)	^{6}I 7% + ^{6}D 74% + 6% + Other 12%<br>(^{6}I 6% + ^{6}D 79% + ^{4}F 6% + Other 9%)
21,501<br>(20,970)	3.4<br>(2.7)	^{6}P 21% + ^{6}I 5% + ^{6}D 54% + ^{6}F 9% + 7% + Other 11%<br>(^{6}I 5% + ^{6}D 81% + Other 14%)
21,567<br>(21,042)	3.0<br>(4.1)	^{6}P 14% + ^{6}D 64% + ^{6}F 7% + 0% + Other 15%<br>(^{6}P 18% + ^{6}I 14% + ^{6}D 47% + ^{4}D 8% + Other 14%)
21,893<br>(21,055)	2.7<br>(4.1)	^{6}P 6% + ^{6}D 76% + 0% + Other 17%<br>(^{6}P 20% + ^{6}I 16% + ^{6}D 44% + ^{4}D 5% + Other 14%)
21,957<br>(21,299)	3.2<br>(3.3)	^{6}P 6% + ^{6}D 80% + 0% + Other 13%<br>(^{6}P 22% + ^{6}I 10% + ^{6}D 52% + Other 16%)

Table S32. Energy (cm^{-1}) and composition of the excited states less than **28,000 cm^{-1}** for [**Cm^{III}Br$_6$**]$^{3-}$. The top text corresponds to LFDFT-PBE results and the text in parentheses to LFDFT-PBE0.

E (cm^{-1})	J	$^{2S+1}$L Composition
0<br>(0)	3.5<br>(3.5)	^{8}S 80% + ^{6}P 17% + Other 3%<br>(^{8}S 81% + ^{6}P 16% + Other 3%)
10<br>(8)	3.5<br>(3.5)	^{8}S 80% + ^{6}P 17% + Other 3%<br>(^{8}S 81% + ^{6}P 16% + Other 3%)
17<br>(17,236)	3.5<br>(3.5)	^{8}S 80% + ^{6}P 17% + Other 3%<br>(^{8}S 10% + ^{6}P 15% + ^{6}I 5% + ^{6}D 13% + ^{4}D 19% + ^{4}H 6% + ^{4}F 8% + ^{2}F 7% + Other 17%)
16,515<br>(17,462)	3.5<br>(3.5)	^{8}S 10% + ^{6}P 13% + ^{6}I 6% + ^{6}D 12% + ^{4}D 16% + ^{4}H 7% + ^{4}F 8% + ^{2}F 7% + Other 21%<br>(^{8}S 10% + ^{6}P 16% + ^{6}I 5% + ^{6}D 13% + ^{4}D 19% + ^{4}H 6% + ^{4}F 8% + ^{2}F 7% + Other 16%)
16,967<br>(17,717)	3.5<br>(3.5)	^{8}S 10% + ^{6}P 15% + ^{6}I 6% + ^{6}D 13% + ^{4}D 17% + ^{4}H 6% + ^{4}F 8% + ^{2}F 7% + Other 19%<br>(^{8}S 10% + ^{6}P 18% + ^{6}D 14% + ^{4}D 20% + ^{4}F 8% + ^{2}F 7% + Other 24%)
17,481<br>(20,273)	3.5<br>(2.5)	^{8}S 11% + ^{6}P 17% + ^{6}D 15% + ^{4}D 19% + ^{4}F 8% + ^{2}F 7% + Other 24%<br>(^{6}P 47% + ^{6}D 29% + ^{4}D 11% + Other 12%)
19,738<br>(20,477)	2.6<br>(2.5)	^{6}P 44% + ^{6}D 29% + ^{4}D 10% + Other 16%<br>(^{6}P 49% + ^{6}D 29% + ^{4}D 11% + Other 11%)
20,124<br>(21,267)	2.7<br>(3.5)	^{6}P 46% + ^{6}D 27% + ^{4}D 10% + Other 17%<br>(^{6}P 9% + ^{6}I 46% + ^{6}D 7% + ^{4}H 23% + Other 16%)
20,793<br>(21,389)	3.5<br>(3.5)	^{6}P 9% + ^{6}I 44% + ^{6}D 7% + ^{4}H 22% + Other 18%<br>(^{6}P 9% + ^{6}I 46% + ^{6}D 7% + ^{4}H 23% + Other 15%)
21,008<br>(21,409)	3.5<br>(3.5)	^{6}P 10% + ^{6}I 42% + ^{6}D 9% + ^{4}H 21% + Other 17%<br>(^{6}P 7% + ^{6}I 49% + ^{6}D 6% + ^{4}H 24% + Other 14%)
21,058<br>(22,320)	3.5<br>(1.8)	^{6}P 5% + ^{6}I 49% + ^{6}D 5% + ^{4}H 25% + Other 15%<br>(^{6}P 60% + ^{6}D 21% + ^{4}S 7% + Other 12%)
21,893<br>(22,575)	2.9<br>(4.5)	^{6}P 41% + ^{6}I 18% + ^{6}D 16% + ^{4}H 7% + Other 18%<br>(^{6}I 53% + ^{6}D 5% + ^{4}H 20% + ^{4}G 5% + Other 16%)
22,083<br>(22,799)	3.9<br>(4.5)	^{6}P 17% + ^{6}I 38% + ^{6}D 9% + ^{4}H 15% + Other 21%<br>(^{6}I 54% + ^{6}D 5% + ^{4}H 20% + Other 20%)

22,490<br>(22,912)	4.4<br>(4.5)	^{6}I 50% + ^{6}D 7% + ^{4}H 19% + Other 24%<br>(^{6}I 56% + ^{6}D 5% + ^{4}H 20% + Other 19%)
22,707<br>(23,892)	4.6<br>(8.4)	^{6}I 54% + ^{6}D 6% + ^{4}H 20% + Other 20%<br>(^{6}I 70% + ^{4}K 21% + Other 8%)
23,443<br>(23,920)	7.8<br>(8.5)	^{6}I 68% + ^{4}K 17% + Other 15%<br>(^{6}I 71% + ^{4}K 21% + Other 8%)
23,476<br>(23,947)	8.3<br>(8.5)	^{6}I 68% + ^{4}K 21% + Other 11%<br>(^{6}I 71% + ^{4}K 21% + Other 8%)
23,512<br>(23,959)	8.3<br>(8.5)	^{6}I 68% + ^{4}K 21% + Other 11%<br>(^{6}I 71% + ^{4}K 21% + Other 7%)
23,530<br>(24,317)	8.5<br>(5.7)	^{6}I 69% + ^{4}K 22% + Other 9%<br>(^{6}I 73% + ^{4}H 13% + Other 14%)
23,595<br>(24,360)	8.3<br>(5.6)	^{6}I 69% + ^{4}K 21% + Other 10%<br>(^{6}I 73% + ^{4}H 13% + Other 14%)
23,633<br>(24,489)	8.4<br>(5.5)	^{6}I 70% + ^{4}K 21% + Other 9%<br>(^{6}I 74% + ^{4}H 14% + Other 13%)
23,899<br>(24,565)	6.4<br>(5.5)	^{6}I 71% + ^{4}H 10% + ^{4}K 7% + Other 12%<br>(^{6}I 74% + ^{4}H 14% + Other 12%)
23,940<br>(25,074)	5.9<br>(6.4)	^{6}I 70% + ^{4}H 12% + Other 19%<br>(^{6}I 75% + ^{4}H 6% + ^{4}K 5% + Other 14%)
24,187<br>(25,136)	5.6<br>(6.5)	^{6}I 72% + ^{4}H 14% + Other 14%<br>(^{6}I 77% + ^{4}H 7% + ^{4}K 5% + Other 11%)
24,351<br>(25,187)	5.5<br>(6.5)	^{6}I 73% + ^{4}H 14% + Other 14%<br>(^{6}I 77% + ^{4}H 7% + ^{4}K 5% + Other 10%)
24,533<br>(25,278)	6.2<br>(6.5)	^{6}I 66% + ^{6}D 7% + ^{4}H 6% + Other 21%<br>(^{6}I 77% + ^{4}H 7% + ^{4}K 6% + Other 10%)
24,731<br>(25,291)	6.5<br>(6.5)	^{6}I 73% + ^{4}H 6% + ^{4}K 6% + Other 15%<br>(^{6}I 77% + ^{4}H 7% + ^{4}K 6% + Other 10%)
24,871<br>(25,308)	6.5<br>(7.4)	^{6}I 76% + ^{4}H 7% + ^{4}K 6% + Other 11%<br>(^{6}I 79% + ^{4}K 11% + Other 10%)
24,881<br>(25,357)	7.2<br>(7.4)	^{6}I 76% + ^{4}K 10% + Other 14%<br>(^{6}I 80% + ^{4}K 11% + Other 9%)
24,988<br>(25,433)	7.3<br>(7.5)	^{6}I 77% + ^{4}K 11% + Other 12%<br>(^{6}I 81% + ^{4}K 12% + Other 7%)
25,025<br>(25,510)	6.5<br>(7.5)	^{6}I 75% + ^{4}H 7% + ^{4}K 6% + Other 11%<br>(^{6}I 81% + ^{4}K 12% + Other 7%)
25,042<br>(25,516)	6.5<br>(7.5)	^{6}I 75% + ^{4}H 8% + ^{4}K 6% + Other 12%<br>(^{6}I 81% + ^{4}K 12% + Other 7%)
25,163<br>(25,840)	7.5<br>(4.6)	^{6}I 80% + ^{4}K 12% + Other 8%<br>(^{6}I 18% + ^{6}D 48% + ^{6}F 13% + ^{4}F 10% + Other 11%)
25,269<br>(26,032)	7.3<br>(4.6)	^{6}I 77% + ^{4}K 12% + Other 11%<br>(^{6}I 18% + ^{6}D 49% + ^{6}F 12% + ^{4}F 11% + Other 11%)
25,282<br>(26,252)	7.4<br>(4.6)	^{6}I 79% + ^{4}K 12% + Other 8%<br>(^{6}I 17% + ^{6}D 51% + ^{6}F 11% + ^{4}F 11% + Other 10%)
25,352	5.1	^{6}I 29% + ^{6}D 37% + ^{6}F 11% + ^{4}F 8% + Other 15%
25,730	4.9	^{6}I 24% + ^{6}D 44% + ^{6}F 11% + ^{4}F 10% + Other 12%
26,086	4.7	^{6}I 20% + ^{6}D 48% + ^{6}F 9% + ^{4}F 12% + Other 11%
27,865	3.6	^{6}P 9% + ^{6}D 37% + ^{6}F 12% + ^{4}D 19% + ^{2}F 6% + Other 16%

27,890	3.6	^{6}P 10% + ^{6}D 37% + ^{6}F 12% + ^{4}D 19% + ^{2}F 6% + Other 17%

Table S33. Energy (cm^{-1}) and composition of the excited states less than **17,000 cm^{-1}** for [**Bk^{IV}Br$_6$**]$^{2-}$. The top text corresponds to LFDFT-PBE results and the text in parentheses to LFDFT-PBE0.

E (cm^{-1})	J	$^{2S+1}$L Composition
0<br>(0)	3.5<br>(3.5)	^{8}S 55% + ^{6}P 27% + ^{4}D 8% + Other 10%<br>(^{8}S 60% + ^{6}P 27% + ^{4}D 7% + Other 6%)
179<br>(51)	3.5<br>(3.5)	^{8}S 59% + ^{6}P 28% + ^{4}D 7% + Other 7%<br>(^{8}S 62% + ^{6}P 27% + ^{4}D 6% + Other 5%)
281<br>(93)	3.5<br>(3.5)	^{8}S 61% + ^{6}P 28% + ^{4}D 6% + Other 5%<br>(^{8}S 62% + ^{6}P 27% + ^{4}D 6% + Other 5%)
6,227<br>(7,589)	3.6<br>(3.5)	^{8}S 17% + ^{6}I 7% + ^{4}D 6% + ^{4}H 15% + ^{4}F 8% + ^{4}G 7% + ^{2}G 8% + ^{2}F 7% + Other 24%<br>(^{8}S 15% + ^{6}I 7% + ^{4}D 11% + ^{4}H 14% + ^{4}F 10% + ^{4}G 8% + ^{2}G 9% + ^{2}F 9% + Other 19%)
7,755<br>(8,119)	3.6<br>(3.5)	^{8}S 16% + ^{6}I 7% + ^{6}D 5% + ^{4}D 9% + ^{4}H 13% + ^{4}F 8% + ^{4}G 6% + ^{2}G 8% + ^{2}F 9% + Other 19%<br>(^{8}S 15% + ^{6}I 7% + ^{6}D 5% + ^{4}D 12% + ^{4}H 13% + ^{4}F 9% + ^{4}G 7% + ^{2}G 9% + ^{2}F 9% + Other 13%)
9,214<br>(8,827)	3.7<br>(3.5)	^{8}S 17% + ^{6}I 5% + ^{6}D 8% + ^{4}D 12% + ^{4}H 9% + ^{4}F 9% + ^{2}G 7% + ^{2}F 9% + Other 24%<br>(^{8}S 15% + ^{6}I 6% + ^{6}D 6% + ^{4}D 14% + ^{4}H 12% + ^{4}F 10% + ^{4}G 6% + ^{2}G 8% + ^{2}F 10% + Other 13%)
10,081<br>(11,239)	3.0<br>(2.6)	^{6}P 22% + ^{6}D 23% + ^{6}G 5% + ^{6}F 8% + ^{4}D 11% + ^{4}F 9% + Other 21%<br>(^{6}P 30% + ^{6}D 27% + ^{6}F 7% + ^{4}D 15% + ^{4}F 9% + Other 12%)
10,513<br>(11,759)	4.3<br>(3.0)	^{6}I 24% + ^{6}D 10% + ^{4}H 21% + ^{6}H 6% + ^{4}F 6% + ^{4}G 8% + Other 25%<br>(^{6}P 27% + ^{6}I 7% + ^{6}D 22% + ^{4}D 14% + ^{4}F 8% + Other 22%)
11,583<br>(12,142)	3.3<br>(3.9)	^{6}P 29% + ^{6}D 23% + ^{6}F 5% + ^{4}D 12% + ^{4}F 6% + Other 25%<br>(^{6}P 9% + ^{6}I 22% + ^{6}D 15% + ^{4}H 18% + ^{4}F 6% + ^{4}G 6% + Other 24%)
11,686<br>(12,306)	4.5<br>(3.7)	^{6}I 28% + ^{6}D 11% + ^{6}F 8% + ^{4}H 21% + ^{2}G 5% + Other 27%<br>(^{8}S 6% + ^{6}I 31% + ^{6}D 11% + ^{4}D 5% + ^{4}H 24% + Other 22%)
11,901<br>(12,315)	3.7<br>(3.5)	^{8}S 10% + ^{6}P 7% + ^{6}I 26% + ^{6}D 11% + ^{4}D 6% + ^{4}H 20% + Other 20%<br>(^{8}S 10% + ^{6}P 6% + ^{6}I 29% + ^{6}D 12% + ^{4}D 9% + ^{4}H 22% + Other 13%)
11,949<br>(12,605)	4.5<br>(4.2)	^{6}P 9% + ^{6}I 20% + ^{6}D 12% + ^{6}F 7% + ^{4}H 14% + ^{4}F 5% + ^{4}G 6% + Other 26%<br>(^{6}I 29% + ^{6}D 9% + ^{4}H 22% + ^{4}F 7% + ^{4}G 8% + Other 26%)
12,697<br>(12,997)	5.7<br>(4.3)	^{6}I 32% + ^{6}D 5% + ^{4}H 15% + ^{4}K 7% + ^{4}G 7% + Other 34%<br>(^{6}I 30% + ^{6}D 10% + ^{4}H 22% + ^{4}F 7% + ^{4}G 7% + Other 25%)
12,742<br>(13,237)	6.7<br>(4.6)	^{6}I 32% + ^{6}F 6% + ^{4}H 8% + ^{4}K 14% + ^{4}G 6% + Other 34%<br>(^{6}I 28% + ^{6}D 11% + ^{4}H 21% + ^{4}F 9% + ^{4}G 8% + Other 22%)
13,174<br>(13,599)	5.2<br>(2.3)	^{6}I 31% + ^{6}D 12% + ^{6}F 8% + ^{4}H 12% + Other 37%<br>(^{6}P 44% + ^{6}I 5% + ^{6}D 21% + ^{4}S 10% + Other 20%)
13,240<br>(13,885)	6.3<br>(7.8)	^{6}I 30% + ^{6}D 6% + ^{4}H 10% + ^{4}K 11% + Other 42%<br>(^{6}I 44% + ^{4}K 23% + ^{2}L 7% + Other 26%)
13,315<br>(13,917)	7.2<br>(8.2)	^{6}I 34% + ^{4}H 8% + ^{4}K 17% + ^{2}L 7% + Other 34%<br>(^{6}I 44% + ^{4}K 27% + ^{2}L 9% + Other 21%)
13,335<br>(13,978)	5.0<br>(8.0)	^{6}P 22% + ^{6}I 20% + ^{6}D 11% + ^{4}H 7% + ^{4}K 7% + Other 33%<br>(^{6}I 44% + ^{4}K 25% + ^{2}L 8% + Other 23%)
13,471<br>(14,052)	7.2<br>(8.4)	^{6}I 34% + ^{4}K 18% + ^{2}L 6% + Other 41%<br>(^{6}I 46% + ^{4}K 29% + ^{2}L 9% + Other 16%)
13,584<br>(14,127)	7.1<br>(8.1)	^{6}I 40% + ^{4}H 7% + ^{4}K 17% + ^{2}L 5% + Other 31%<br>(^{6}I 45% + ^{4}K 26% + ^{2}L 8% + Other 21%)
13,821<br>(14,195)	7.7<br>(8.3)	^{6}I 37% + ^{4}K 23% + ^{2}L 8% + Other 32%<br>(^{6}I 47% + ^{4}K 28% + ^{2}L 9% + Other 17%)
13,938<br>(14,586)	7.1<br>(6.6)	^{6}I 43% + ^{4}H 6% + ^{4}K 16% + Other 34%<br>(^{6}I 50% + ^{4}H 13% + ^{4}K 10% + Other 27%)

14,032<br>(14,803)	6.0<br>(5.7)	^{6}I 46% + ^{4}H 16% + ^{4}K 8% + Other 29%<br>(^{6}I 50% + ^{4}H 17% + ^{4}K 5% + ^{4}G 6% + Other 22%)
14,355<br>(15,048)	6.0<br>(5.9)	^{6}I 42% + ^{4}H 15% + ^{4}K 7% + Other 37%<br>(^{6}I 48% + ^{4}H 15% + Other 37%)
14,641<br>(15,333)	7.1<br>(6.4)	^{6}I 50% + ^{4}H 8% + ^{4}K 16% + Other 26%<br>(^{6}I 51% + ^{4}H 11% + ^{4}K 8% + Other 29%)
14,829<br>(15,452)	6.3<br>(6.5)	^{6}I 45% + ^{4}H 9% + ^{6}H 6% + ^{4}K 8% + Other 32%<br>(^{6}I 51% + ^{4}H 13% + ^{4}K 9% + ^{4}I 6% + Other 21%)
14,986<br>(15,535)	6.7<br>(6.5)	^{6}I 51% + ^{4}H 9% + ^{6}H 5% + ^{4}K 11% + Other 24%<br>(^{6}I 51% + ^{4}H 14% + ^{4}K 9% + ^{4}I 6% + Other 21%)
15,081<br>(15,539)	6.7<br>(6.5)	^{6}I 38% + ^{4}H 12% + ^{4}K 11% + Other 39%<br>(^{6}I 50% + ^{4}H 14% + ^{4}K 9% + ^{4}I 6% + Other 22%)
15,120<br>(15,613)	5.9<br>(6.8)	^{6}I 43% + ^{6}F 5% + ^{4}H 13% + ^{4}K 6% + Other 33%<br>(^{6}I 54% + ^{6}D 5% + ^{4}K 13% + Other 28%)
15,251<br>(15,687)	6.8<br>(5.4)	^{6}I 44% + ^{4}H 11% + ^{4}K 13% + Other 33%<br>(^{6}I 39% + ^{6}D 15% + ^{6}F 8% + ^{4}H 10% + ^{4}F 5% + Other 23%)
15,359<br>(15,762)	6.3<br>(6.9)	^{6}I 46% + ^{4}H 12% + ^{4}K 8% + Other 33%<br>(^{6}I 56% + ^{6}D 5% + ^{4}K 14% + Other 25%)
15,424<br>(16,031)	5.6<br>(7.3)	^{6}I 30% + ^{6}D 5% + ^{6}F 6% + ^{4}D 7% + ^{4}K 8% + Other 43%<br>(^{6}I 57% + ^{4}K 16% + Other 27%)
15,462<br>(16,079)	6.5<br>(7.0)	^{6}I 48% + ^{6}D 6% + ^{4}H 8% + ^{4}K 10% + Other 28%<br>(^{6}I 55% + ^{4}K 16% + Other 30%)
15,533<br>(16,135)	6.0<br>(5.7)	^{6}I 39% + ^{6}D 9% + ^{6}F 6% + ^{4}H 7% + ^{4}K 8% + Other 30%<br>(^{6}I 38% + ^{6}D 19% + ^{6}F 9% + ^{4}K 6% + ^{4}F 6% + Other 22%)
15,806<br>(16,425)	6.2<br>(5.2)	^{6}P 5% + ^{6}I 39% + ^{4}K 12% + Other 44%<br>(^{6}I 31% + ^{6}D 26% + ^{6}F 10% + ^{4}F 9% + Other 23%)
15,833	6.5	^{6}I 44% + ^{6}D 5% + ^{4}K 12% + Other 38%
16,333	5.5	^{6}I 35% + ^{6}D 21% + ^{6}F 8% + ^{4}K 6% + ^{4}F 7% + Other 24%
16,780	5.2	^{6}I 33% + ^{6}D 27% + ^{6}F 5% + ^{4}F 12% + Other 23%
16,805	5.0	^{6}I 26% + ^{6}D 19% + ^{6}F 6% + ^{4}D 9% + ^{4}K 5% + ^{2}F 9% + Other 27%

Table S34. Energy (cm^{-1}) and composition of the excited states less than **44,000 cm^{-1}** for **$[Gd^{III}(H_2O)_9]^{3+}$**. The top text corresponds to LFDFT-PBE results and the text in parentheses to LFDFT-PBE0.

E (cm^{-1})	J	$^{2S+1}$L Composition
1<br>(0)	3.5<br>(3.5)	^{8}S 97% + Other 3%<br>(^{8}S 97% + Other 3%)
32,885<br>(33,816)	3.5<br>(3.5)	^{6}P 76% + ^{6}D 12% + ^{4}D 7% + Other 4%<br>(^{6}P 77% + ^{6}D 12% + ^{4}D 7% + Other 4%)
32,948<br>(33,852)	3.5<br>(3.5)	^{6}P 77% + ^{6}D 12% + ^{4}D 7% + Other 5%<br>(^{6}P 78% + ^{6}D 11% + ^{4}D 7% + Other 4%)
32,991<br>(33,874)	3.5<br>(3.5)	^{6}P 77% + ^{6}D 11% + ^{4}D 7% + Other 4%<br>(^{6}P 78% + ^{6}D 11% + ^{4}D 7% + Other 4%)
33,010<br>(33,884)	3.5<br>(3.5)	^{6}P 77% + ^{6}D 11% + ^{4}D 7% + Other 5%<br>(^{6}P 78% + ^{6}D 11% + ^{4}D 7% + Other 4%)

33,481<br>(34,395)	2.6<br>(2.5)	^{6}P 83% + ^{6}D 12% + Other 4%<br>(^{6}P 85% + ^{6}D 12% + Other 4%)
33,538<br>(34,430)	2.6<br>(2.5)	^{6}P 84% + ^{6}D 12% + Other 4%<br>(^{6}P 85% + ^{6}D 12% + Other 3%)
33,567<br>(34,443)	2.6<br>(2.5)	^{6}P 84% + ^{6}D 12% + Other 4%<br>(^{6}P 85% + ^{6}D 12% + Other 3%)
34,057<br>(34,953)	1.6<br>(1.6)	^{6}P 90% + ^{6}D 7% + Other 3%<br>(^{6}P 91% + ^{6}D 7% + Other 3%)
34,110<br>(34,981)	1.6<br>(1.6)	^{6}P 90% + ^{6}D 6% + Other 3%<br>(^{6}P 91% + ^{6}D 6% + Other 2%)
36,152<br>(37,088)	3.5<br>(3.5)	^{6}I 92% + ^{4}H 7% + Other 1%<br>(^{6}I 93% + ^{4}H 6% + Other 1%)
36,164<br>(37,101)	3.5<br>(3.5)	^{6}I 92% + ^{4}H 7% + Other 1%<br>(^{6}I 93% + ^{4}H 6% + Other 1%)
36,176<br>(37,449)	3.5<br>(4.5)	^{6}I 92% + ^{4}H 7% + Other 1%<br>(^{6}I 95% + Other 5%)
36,518<br>(37,469)	4.5<br>(4.5)	^{6}I 94% + Other 6%<br>(^{6}I 95% + Other 5%)
36,535<br>(37,676)	4.5<br>(8.5)	^{6}I 94% + Other 6%<br>(^{6}I 96% + Other 4%)
36,547<br>(37,683)	4.5<br>(8.5)	^{6}I 95% + Other 5%<br>(^{6}I 96% + Other 4%)
36,551<br>(37,750)	4.5<br>(5.5)	^{6}I 95% + Other 5%<br>(^{6}I 96% + Other 4%)
36,748<br>(37,766)	8.5<br>(5.5)	^{6}I 96% + Other 4%<br>(^{6}I 96% + Other 4%)
36,756<br>(37,774)	8.5<br>(5.5)	^{6}I 96% + Other 4%<br>(^{6}I 96% + Other 4%)
36,762<br>(37,932)	8.5<br>(6.7)	^{6}I 96% + Other 4%<br>(^{6}I 97% + Other 3%)
36,773<br>(37,946)	8.4<br>(6.7)	^{6}I 95% + Other 5%<br>(^{6}I 97% + Other 3%)
36,822<br>(37,965)	5.5<br>(7.5)	^{6}I 96% + Other 4%<br>(^{6}I 97% + Other 3%)
36,841<br>(37,975)	5.5<br>(7.4)	^{6}I 96% + Other 4%<br>(^{6}I 97% + Other 3%)
36,851<br>(42,041)	5.5<br>(4.5)	^{6}I 96% + Other 4%<br>(^{6}D 93% + Other 7%)
36,858<br>(42,046)	5.5<br>(4.5)	^{6}I 96% + Other 4%<br>(^{6}D 93% + Other 7%)
36,863<br>(42,060)	5.5<br>(4.5)	^{6}I 96% + Other 4%<br>(^{6}D 93% + Other 7%)
37,000<br>(42,084)	6.8<br>(4.5)	^{6}I 97% + Other 3%<br>(^{6}D 93% + Other 7%)
37,020<br>(42,102)	6.8<br>(4.5)	^{6}I 97% + Other 3%<br>(^{6}D 94% + Other 6%)

37,041<br>(42,883)	7.3<br>(0.6)	^{6}I 97% + Other 3%<br>(^{6}D 99% + Other 1%)
37,051<br>(43,014)	7.5<br>(3.5)	^{6}I 97% + Other 3%<br>(^{6}P 10% + ^{6}D 83% + Other 7%)
37,058<br>(43,026)	6.9<br>(3.5)	^{6}I 97% + Other 3%<br>(^{6}P 11% + ^{6}D 82% + Other 7%)
37,068<br>(43,112)	7.3<br>(1.6)	^{6}I 97% + Other 3%<br>(^{6}P 7% + ^{6}D 91% + Other 3%)
37,074<br>(43,150)	7.2<br>(1.5)	^{6}I 97% + Other 3%<br>(^{6}P 7% + ^{6}D 90% + Other 3%)
40,976<br>(43,269)	4.5<br>(2.5)	^{6}D 93% + Other 7%<br>(^{6}P 12% + ^{6}D 83% + Other 5%)
40,995<br>(43,277)	4.5<br>(2.5)	^{6}D 93% + Other 7%<br>(^{6}P 12% + ^{6}D 83% + Other 5%)
41,020<br>(43,290)	4.5<br>(2.5)	^{6}D 93% + Other 7%<br>(^{6}P 12% + ^{6}D 83% + Other 5%)
41,057	4.5	^{6}D 93% + Other 7%
41,086	4.5	^{6}D 93% + Other 7%
41,853	0.6	^{6}D 98% + Other 2%
41,984	3.5	^{6}P 11% + ^{6}D 82% + Other 7%
41,997	3.5	^{6}P 11% + ^{6}D 81% + Other 8%
42,006	3.5	^{6}P 12% + ^{6}D 80% + Other 8%
42,075	1.7	^{6}P 7% + ^{6}D 90% + Other 3%
42,148	1.5	^{6}P 7% + ^{6}D 90% + Other 3%
42,251	2.5	^{6}P 13% + ^{6}D 82% + Other 5%
42,261	2.5	^{6}P 12% + ^{6}D 83% + Other 5%
42,278	2.5	^{6}P 12% + ^{6}D 83% + Other 5%

Table S35. Energy (cm^{-1}) and composition of the excited states less than **24,000 cm^{-1}** for **[Tb^{IV}(H$_2$O)$_9$]$^{4+}$**. The text corresponds to LFDFT-PBE results.

**E (cm^{-1})**	**J**	**$^{2S+1}$L Composition**
0	3.5	^{8}S 95% + ^{6}P 5%
9	3.5	^{8}S 95% + Other 5%
19,593	3.6	^{6}P 57% + ^{6}D 19% + ^{4}D 11% + Other 12%
19,880	3.6	^{6}P 60% + ^{6}D 17% + ^{4}D 11% + Other 12%

20,160	3.7	^{6}P 62% + ^{6}D 14% + ^{4}D 12% + Other 12%
20,277	3.7	^{6}P 63% + ^{6}D 13% + ^{4}D 12% + Other 13%
20,446	3.0	^{6}P 67% + ^{6}I 8% + ^{6}D 17% + Other 8%
20,587	2.8	^{6}P 70% + ^{6}I 6% + ^{6}D 17% + Other 6%
20,789	2.8	^{6}P 72% + ^{6}I 6% + ^{6}D 16% + Other 6%
21,201	2.3	^{6}P 77% + ^{6}I 7% + ^{6}D 11% + Other 5%
21,492	2.5	^{6}P 79% + ^{6}I 9% + ^{6}D 7% + Other 5%
22,308	3.6	^{6}I 82% + ^{4}H 12% + Other 6%
22,381	3.6	^{6}I 82% + ^{4}H 12% + Other 6%
22,471	3.6	^{6}I 84% + ^{4}H 12% + Other 4%
22,576	3.6	^{6}I 83% + ^{4}H 12% + Other 5%
22,762	4.6	^{6}I 86% + ^{4}H 8% + Other 6%
22,849	4.6	^{6}I 87% + ^{4}H 8% + Other 5%
22,936	4.6	^{6}I 89% + ^{4}H 8% + Other 3%
22,989	4.6	^{6}I 89% + ^{4}H 8% + Other 3%
23,023	5.2	^{6}I 89% + ^{4}H 7% + Other 4%
23,060	7.9	^{6}I 90% + ^{4}K 6% + Other 4%
23,090	8.0	^{6}I 91% + ^{4}K 6% + Other 3%
23,116	8.1	^{6}I 91% + ^{4}K 6% + Other 3%
23,133	6.8	^{6}I 90% + Other 10%
23,156	8.2	^{6}I 91% + ^{4}K 7% + Other 3%
23,173	7.9	^{6}I 91% + ^{4}K 6% + Other 4%
23,198	8.3	^{6}I 91% + ^{4}K 7% + Other 3%
23,221	6.8	^{6}I 90% + Other 10%
23,240	8.0	^{6}I 90% + ^{4}K 6% + Other 4%
23,253	7.9	^{6}I 90% + ^{4}K 6% + Other 5%
23,269	7.1	^{6}I 90% + Other 10%
23,300	7.0	^{6}I 89% + Other 11%
23,331	6.4	^{6}I 92% + Other 8%

23,346	6.3	^{6}I 92% + Other 8%
23,385	6.6	^{6}I 92% + Other 8%
23,391	6.0	^{6}I 92% + Other 8%
23,431	6.1	^{6}I 92% + Other 8%
23,453	6.8	^{6}I 93% + Other 7%
23,508	6.9	^{6}I 93% + Other 7%
23,537	7.2	^{6}I 93% + Other 7%
23,552	7.1	^{6}I 94% + Other 6%
23,565	6.9	^{6}I 94% + Other 6%

Table S36. Energy (cm^{-1}) and composition of the excited states less than **30,000 cm^{-1}** for [**Cm^{III}(H$_2$O)$_9$**]$^{3+}$. The top text corresponds to LFDFT-PBE results and the text in parentheses to LFDFT-PBE0.

E (cm^{-1})	J	$^{2S+1}$L Composition
0<br>(0)	3.5<br>(3.5)	^{8}S 83% + ^{6}P 15% + Other 2%<br>(^{8}S 83% + ^{6}P 14% + Other 2%)
9<br>(13)	3.5<br>(3.5)	^{8}S 83% + ^{6}P 15% + Other 2%<br>(^{8}S 84% + ^{6}P 14% + Other 2%)
16<br>(20,934)	3.5<br>(3.5)	^{8}S 83% + ^{6}P 15% + Other 2%<br>(^{8}S 9% + ^{6}P 20% + ^{6}I 5% + ^{6}D 15% + ^{4}D 18% + ^{4}F 7% + ^{2}F 6% + Other 20%)
20,181<br>(21,167)	3.5<br>(3.5)	^{8}S 9% + ^{6}P 18% + ^{6}I 6% + ^{6}D 14% + ^{4}D 17% + ^{4}H 5% + ^{4}F 7% + ^{2}F 6% + Other 17%<br>(^{8}S 9% + ^{6}P 21% + ^{6}D 15% + ^{4}D 18% + ^{4}F 7% + ^{2}F 6% + Other 24%)
20,453<br>(21,251)	3.5<br>(3.5)	^{8}S 9% + ^{6}P 19% + ^{6}I 5% + ^{6}D 14% + ^{4}D 18% + ^{4}H 5% + ^{4}F 7% + ^{2}F 6% + Other 16%<br>(^{8}S 9% + ^{6}P 22% + ^{6}D 16% + ^{4}D 18% + ^{4}F 7% + ^{2}F 6% + Other 22%)
20,562<br>(21,374)	3.5<br>(3.5)	^{8}S 10% + ^{6}P 20% + ^{6}D 15% + ^{4}D 18% + ^{4}F 7% + ^{2}F 6% + Other 24%<br>(^{8}S 10% + ^{6}P 23% + ^{6}D 16% + ^{4}D 19% + ^{4}F 7% + ^{2}F 6% + Other 21%)
20,712<br>(23,924)	3.5<br>(2.7)	^{8}S 10% + ^{6}P 21% + ^{6}D 15% + ^{4}D 19% + ^{4}F 7% + ^{2}F 6% + Other 22%<br>(^{6}P 48% + ^{6}D 27% + ^{4}D 9% + Other 16%)
23,293<br>(24,116)	2.7<br>(2.6)	^{6}P 47% + ^{6}D 27% + ^{4}D 9% + Other 18%<br>(^{6}P 50% + ^{6}D 28% + ^{4}D 9% + Other 13%)
23,520<br>(24,161)	2.7<br>(2.6)	^{6}P 49% + ^{6}D 27% + ^{4}D 9% + Other 15%<br>(^{6}P 51% + ^{6}D 28% + ^{4}D 9% + Other 12%)
23,572<br>(25,253)	2.6<br>(3.5)	^{6}P 50% + ^{6}D 28% + ^{4}D 9% + Other 14%<br>(^{6}P 7% + ^{6}I 51% + ^{6}D 5% + ^{4}H 23% + Other 13%)
24,608<br>(25,297)	3.5<br>(3.5)	^{6}P 8% + ^{6}I 49% + ^{6}D 6% + ^{4}H 23% + Other 14%<br>(^{6}P 7% + ^{6}I 53% + ^{4}H 23% + Other 17%)
24,663<br>(25,383)	3.5<br>(3.5)	^{6}P 7% + ^{6}I 51% + ^{4}H 23% + Other 18%<br>(^{6}P 9% + ^{6}I 50% + ^{6}D 6% + ^{4}H 22% + Other 13%)
24,764<br>(25,415)	3.5<br>(3.5)	^{6}P 9% + ^{6}I 48% + ^{6}D 7% + ^{4}H 22% + Other 14%<br>(^{6}P 9% + ^{6}I 50% + ^{6}D 6% + ^{4}H 22% + Other 13%)

24,817<br>(26,027)	3.5<br>(1.9)	^{6}P 10% + ^{6}I 47% + ^{6}D 7% + ^{4}H 22% + Other 14%<br>(^{6}P 62% + ^{6}D 20% + ^{4}S 6% + Other 12%)
25,444<br>(26,085)	2.1<br>(2.1)	^{6}P 59% + ^{6}I 6% + ^{6}D 20% + ^{4}S 6% + Other 9%<br>(^{6}P 60% + ^{6}I 7% + ^{6}D 19% + ^{4}S 6% + Other 9%)
25,510<br>(26,777)	2.3<br>(4.4)	^{6}P 56% + ^{6}I 10% + ^{6}D 18% + ^{4}S 6% + Other 11%<br>(^{6}I 56% + ^{6}D 5% + ^{4}H 18% + Other 20%)
26,119<br>(26,858)	4.3<br>(4.5)	^{6}P 5% + ^{6}I 52% + ^{6}D 6% + ^{4}H 18% + Other 18%<br>(^{6}I 60% + ^{4}H 19% + Other 21%)
26,209<br>(26,906)	4.5<br>(4.5)	^{6}I 58% + ^{4}H 19% + Other 23%<br>(^{6}I 59% + ^{4}H 19% + Other 22%)
26,281<br>(26,947)	4.4<br>(4.5)	^{6}I 56% + ^{6}D 5% + ^{4}H 19% + Other 20%<br>(^{6}I 60% + ^{4}H 19% + Other 21%)
26,318<br>(26,986)	4.5<br>(4.5)	^{6}I 57% + ^{4}H 19% + Other 24%<br>(^{6}I 61% + ^{4}H 19% + Other 19%)
26,365<br>(28,055)	4.5<br>(8.5)	^{6}I 59% + ^{4}H 20% + Other 22%<br>(^{6}I 74% + ^{4}K 20% + Other 6%)
27,448<br>(28,066)	8.5<br>(8.5)	^{6}I 73% + ^{4}K 20% + Other 7%<br>(^{6}I 75% + ^{4}K 19% + Other 6%)
27,453<br>(28,080)	8.5<br>(8.5)	^{6}I 73% + ^{4}K 20% + Other 7%<br>(^{6}I 75% + ^{4}K 19% + Other 6%)
27,466<br>(28,086)	8.5<br>(8.5)	^{6}I 73% + ^{4}K 20% + Other 7%<br>(^{6}I 75% + ^{4}K 19% + Other 6%)
27,484<br>(28,096)	8.5<br>(8.5)	^{6}I 73% + ^{4}K 20% + Other 7%<br>(^{6}I 75% + ^{4}K 19% + Other 6%)
27,490<br>(28,106)	8.5<br>(8.5)	^{6}I 73% + ^{4}K 20% + Other 7%<br>(^{6}I 74% + ^{4}K 19% + Other 6%)
27,504<br>(28,486)	8.5<br>(5.5)	^{6}I 73% + ^{4}K 20% + Other 7%<br>(^{6}I 76% + ^{4}H 12% + Other 12%)
27,511<br>(28,526)	8.4<br>(5.5)	^{6}I 72% + ^{4}K 20% + Other 8%<br>(^{6}I 76% + ^{4}H 13% + Other 11%)
27,517<br>(28,567)	8.5<br>(5.5)	^{6}I 73% + ^{4}K 20% + Other 7%<br>(^{6}I 76% + ^{4}H 13% + Other 11%)
27,889<br>(28,603)	5.5<br>(5.5)	^{6}I 74% + ^{4}H 13% + Other 13%<br>(^{6}I 77% + ^{4}H 13% + Other 11%)
27,933<br>(28,631)	5.5<br>(5.5)	^{6}I 75% + ^{4}H 13% + Other 12%<br>(^{6}I 77% + ^{4}H 12% + Other 11%)
27,987<br>(28,671)	5.5<br>(5.5)	^{6}I 75% + ^{4}H 13% + Other 12%<br>(^{6}I 77% + ^{4}H 12% + Other 10%)
28,028<br>(29,282)	5.5<br>(6.5)	^{6}I 75% + ^{4}H 13% + Other 12%<br>(^{6}I 80% + ^{4}H 6% + Other 14%)
28,063<br>(29,343)	5.5<br>(6.5)	^{6}I 75% + ^{4}H 13% + Other 12%<br>(^{6}I 80% + ^{4}H 6% + Other 14%)
28,108<br>(29,361)	5.5<br>(6.5)	^{6}I 76% + ^{4}H 13% + Other 11%<br>(^{6}I 80% + ^{4}H 6% + ^{4}K 5% + Other 9%)
28,701<br>(29,370)	6.5<br>(6.5)	^{6}I 79% + ^{4}H 6% + ^{4}K 5% + Other 10%<br>(^{6}I 81% + ^{4}H 6% + Other 14%)

28,773<br>(29,393)	6.5<br>(6.5)	^{6}I 79% + ^{4}H 6% + ^{4}K 5% + Other 10%<br>(^{6}I 81% + ^{4}H 6% + ^{4}K 5% + Other 9%)
28,795<br>(29,408)	6.5<br>(6.5)	^{6}I 79% + ^{4}H 6% + ^{4}K 5% + Other 10%<br>(^{6}I 80% + ^{4}H 6% + Other 14%)
28,808<br>(29,422)	6.5<br>(6.5)	^{6}I 79% + ^{4}H 6% + ^{4}K 5% + Other 9%<br>(^{6}I 80% + ^{4}H 6% + Other 13%)
28,832<br>(29,494)	6.5<br>(7.4)	^{6}I 79% + ^{4}H 6% + ^{4}K 5% + Other 9%<br>(^{6}I 82% + ^{4}K 10% + Other 8%)
28,849<br>(29,510)	6.5<br>(7.4)	^{6}I 79% + ^{4}H 6% + ^{4}K 5% + Other 9%<br>(^{6}I 83% + ^{4}K 10% + Other 7%)
28,865<br>(29,545)	6.5<br>(7.5)	^{6}I 79% + ^{4}H 6% + ^{4}K 5% + Other 9%<br>(^{6}I 83% + ^{4}K 10% + Other 7%)
28,937<br>(29,565)	7.4<br>(7.5)	^{6}I 81% + ^{4}K 10% + Other 9%<br>(^{6}I 83% + ^{4}K 10% + Other 7%)
28,954<br>(29,578)	7.4<br>(7.5)	^{6}I 82% + ^{4}K 10% + Other 8%<br>(^{6}I 83% + ^{4}K 10% + Other 7%)
28,996<br>(29,600)	7.5<br>(7.5)	^{6}I 82% + ^{4}K 11% + Other 7%<br>(^{6}I 84% + ^{4}K 10% + Other 6%)
29,022<br>(29,619)	7.5<br>(7.4)	^{6}I 82% + ^{4}K 11% + Other 7%<br>(^{6}I 83% + ^{4}K 10% + Other 7%)
29,036<br>(29,637)	7.4<br>(7.4)	^{6}I 82% + ^{4}K 11% + Other 7%<br>(^{6}I 83% + ^{4}K 10% + Other 7%)
29,061<br>(30,358)	7.5<br>(4.5)	^{6}I 83% + ^{4}K 11% + Other 7%<br>(^{6}I 12% + ^{6}D 55% + ^{6}F 13% + ^{4}F 11% + Other 10%)
29,082<br>(30,407)	7.4<br>(4.5)	^{6}I 82% + ^{4}K 11% + Other 7%<br>(^{6}I 13% + ^{6}D 54% + ^{6}F 12% + ^{4}F 11% + Other 9%)
29,104<br>(30,551)	7.4<br>(4.5)	^{6}I 82% + ^{4}K 11% + Other 7%<br>(^{6}I 13% + ^{6}D 56% + ^{6}F 12% + ^{4}F 11% + Other 9%)

Table S37. Energy (cm^{-1}) and composition of the excited states less than **21,000 cm^{-1}** for **[$Bk^{IV}(H_2O)_9$]$^{4+}$**. The top text corresponds to LFDFT-PBE results and the text in parentheses to LFDFT-PBE0.

E (cm^{-1})	J	$^{2S+1}$L Composition
0<br>(0)	3.5<br>(3.5)	^{8}S 69% + ^{6}P 23% + Other 8%<br>(^{8}S 72% + ^{6}P 22% + Other 6%)
58<br>(37)	3.5<br>(3.5)	^{8}S 70% + ^{6}P 23% + Other 7%<br>(^{8}S 72% + ^{6}P 22% + Other 6%)
96<br>(53)	3.5<br>(3.5)	^{8}S 70% + ^{6}P 23% + Other 7%<br>(^{8}S 73% + ^{6}P 22% + Other 6%)
102<br>(60)	3.5<br>(3.5)	^{8}S 70% + ^{6}P 23% + Other 7%<br>(^{8}S 73% + ^{6}P 22% + Other 6%)
11,343<br>(12,767)	3.6<br>(3.5)	^{8}S 12% + ^{6}I 8% + ^{6}D 7% + ^{4}D 12% + ^{4}H 11% + ^{4}F 8% + ^{4}G 5% + ^{2}G 8% + ^{2}F 9% + Other 20%<br>(^{8}S 11% + ^{6}P 5% + ^{6}I 8% + ^{6}D 8% + ^{4}D 13% + ^{4}H 10% + ^{4}F 8% + ^{2}G 7% + ^{2}F 9% + Other 20%)
11,991<br>(13,306)	3.6<br>(3.6)	^{8}S 12% + ^{6}I 8% + ^{6}D 7% + ^{4}D 13% + ^{4}H 11% + ^{4}F 8% + ^{2}G 8% + ^{2}F 9% + Other 24%<br>(^{8}S 11% + ^{6}P 6% + ^{6}I 8% + ^{6}D 8% + ^{4}D 14% + ^{4}H 10% + ^{4}F 8% + ^{2}G 7% + ^{2}F 9% + Other 19%)

12,360<br>(13,589)	3.6<br>(3.6)	^{8}S 13% + ^{6}P 5% + ^{6}I 7% + ^{6}D 8% + ^{4}D 14% + ^{4}H 9% + ^{4}F 9% + ^{2}G 7% + ^{2}F 9% + Other 20%<br>(^{8}S 12% + ^{6}P 7% + ^{6}I 6% + ^{6}D 10% + ^{4}D 15% + ^{4}H 8% + ^{4}F 9% + ^{2}G 6% + ^{2}F 8% + Other 19%)
12,729<br>(13,913)	3.6<br>(3.6)	^{8}S 13% + ^{6}P 6% + ^{6}I 6% + ^{6}D 9% + ^{4}D 15% + ^{4}H 8% + ^{4}F 9% + ^{2}G 7% + ^{2}F 9% + Other 19%<br>(^{8}S 12% + ^{6}P 8% + ^{6}I 6% + ^{6}D 10% + ^{4}D 16% + ^{4}H 7% + ^{4}F 9% + ^{2}G 6% + ^{2}F 8% + Other 18%)
15,367<br>(16,684)	3.0<br>(3.0)	^{6}P 32% + ^{6}I 7% + ^{6}D 24% + ^{6}F 5% + ^{4}D 11% + ^{4}F 5% + Other 15%<br>(^{6}P 34% + ^{6}I 8% + ^{6}D 24% + ^{4}D 11% + Other 23%)
15,935<br>(17,129)	3.0<br>(2.9)	^{6}P 34% + ^{6}I 8% + ^{6}D 24% + ^{4}D 11% + Other 23%<br>(^{6}P 36% + ^{6}I 7% + ^{6}D 24% + ^{4}D 11% + Other 22%)
16,014<br>(17,264)	3.0<br>(2.9)	^{6}P 34% + ^{6}I 8% + ^{6}D 23% + ^{4}D 11% + Other 24%<br>(^{6}P 38% + ^{6}I 6% + ^{6}D 26% + ^{4}D 11% + Other 19%)
16,503<br>(17,840)	3.6<br>(3.6)	^{6}P 8% + ^{6}I 32% + ^{6}D 11% + ^{4}H 22% + Other 27%<br>(^{6}P 9% + ^{6}I 35% + ^{6}D 10% + ^{4}H 22% + Other 24%)
16,664<br>(17,906)	3.8<br>(3.6)	^{6}P 8% + ^{6}I 33% + ^{6}D 10% + ^{4}H 21% + Other 28%<br>(^{6}P 9% + ^{6}I 37% + ^{6}D 9% + ^{4}H 22% + Other 24%)
16,992<br>(18,272)	3.8<br>(3.7)	^{8}S 6% + ^{6}P 9% + ^{6}I 32% + ^{6}D 12% + ^{4}H 20% + Other 21%<br>(^{8}S 5% + ^{6}P 11% + ^{6}I 33% + ^{6}D 12% + ^{4}H 20% + Other 19%)
17,211<br>(18,406)	4.0<br>(3.6)	^{6}P 7% + ^{6}I 30% + ^{6}D 12% + ^{4}H 19% + Other 33%<br>(^{8}S 5% + ^{6}P 12% + ^{6}I 32% + ^{6}D 13% + ^{4}D 5% + ^{4}H 19% + Other 14%)
17,300<br>(18,636)	3.8<br>(4.2)	^{6}P 10% + ^{6}I 30% + ^{6}D 13% + ^{4}H 19% + Other 28%<br>(^{6}P 7% + ^{6}I 32% + ^{6}D 10% + ^{4}H 18% + ^{4}F 6% + ^{4}G 6% + Other 21%)
17,572<br>(18,840)	3.7<br>(3.7)	^{6}P 23% + ^{6}I 26% + ^{6}D 12% + ^{4}H 13% + Other 26%<br>(^{6}P 22% + ^{6}I 28% + ^{6}D 12% + ^{4}H 13% + Other 25%)
17,715<br>(19,117)	4.4<br>(4.4)	^{6}I 33% + ^{6}D 10% + ^{4}H 20% + ^{4}F 6% + ^{4}G 6% + Other 24%<br>(^{6}I 38% + ^{6}D 9% + ^{4}H 20% + ^{4}F 6% + ^{4}G 6% + Other 21%)
17,848<br>(19,175)	4.3<br>(4.3)	^{6}P 8% + ^{6}I 35% + ^{6}D 9% + ^{4}H 19% + ^{4}F 6% + ^{4}G 6% + Other 17%<br>(^{6}P 7% + ^{6}I 37% + ^{6}D 9% + ^{4}H 19% + ^{4}F 6% + ^{4}G 6% + Other 17%)
17,978<br>(19,340)	4.4<br>(4.4)	^{6}I 36% + ^{6}D 9% + ^{4}H 21% + ^{4}F 7% + ^{4}G 7% + Other 21%<br>(^{6}I 38% + ^{6}D 9% + ^{4}H 20% + ^{4}F 7% + ^{4}G 7% + Other 19%)
18,274<br>(19,463)	3.6<br>(3.6)	^{6}P 28% + ^{6}I 21% + ^{6}D 15% + ^{4}H 10% + Other 27%<br>(^{6}P 28% + ^{6}I 22% + ^{6}D 15% + ^{4}H 11% + Other 25%)
18,305<br>(19,520)	3.4<br>(3.2)	^{6}P 35% + ^{6}I 15% + ^{6}D 17% + ^{4}H 6% + ^{4}S 6% + Other 21%<br>(^{6}P 37% + ^{6}I 14% + ^{6}D 18% + ^{4}H 6% + ^{4}S 6% + Other 19%)
19,101<br>(20,499)	8.4<br>(8.4)	^{6}I 54% + ^{4}K 27% + ^{2}L 7% + Other 13%<br>(^{6}I 57% + ^{4}K 26% + ^{2}L 6% +Other 11%)
19,125<br>(20,511)	8.4<br>(8.4)	^{6}I 54% + ^{4}K 27% + ^{2}L 7% + Other 13%<br>(^{6}I 57% + ^{4}K 26% + ^{2}L 6% +Other 11%)
19,150<br>(20,520)	8.4<br>(8.4)	^{6}I 54% + ^{4}K 27% + ^{2}L 7% + Other 12%<br>(^{6}I 57% + ^{4}K 26% + ^{2}L 6% +Other 10%)
19,203<br>(20,556)	8.3<br>(8.4)	^{6}I 54% + ^{4}K 26% + ^{2}L 7% + Other 13%<br>(^{6}I 58% + ^{4}K 26% + ^{2}L 6% +Other 10%)
19,236<br>(20,606)	8.2<br>(8.4)	^{6}I 53% + ^{4}K 25% + ^{2}L 6% + Other 16%<br>(^{6}I 58% + ^{4}K 25% + ^{2}L 6% +Other 11%)
19,301<br>(20,630)	8.3<br>(8.4)	^{6}I 54% + ^{4}K 26% + ^{2}L 7% + Other 13%<br>(^{6}I 58% + ^{4}K 25% + ^{2}L 6% +Other 11%)
19,348<br>(20,680)	8.3<br>(8.3)	^{6}I 53% + ^{4}K 26% + ^{2}L 7% + Other 15%<br>(^{6}I 58% + ^{4}K 25% + ^{2}L 6% +Other 11%)
19,372<br>(20,699)	8.3<br>(8.3)	^{6}I 54% + ^{4}K 26% + ^{2}L 7% + Other 13%<br>(^{6}I 56% + ^{4}K 25% + ^{2}L 6% +Other 13%)
19,420<br>(20,726)	8.3<br>(8.3)	^{6}I 55% + ^{4}K 26% + ^{2}L 7% + Other 12%<br>(^{6}I 59% + ^{4}K 25% + ^{2}L 6% +Other 10%)

19,578<br>(21,002)	5.6<br>(5.6)	^{6}I 53% + ^{4}H 15% + Other 32%<br>(^{6}I 58% + ^{4}H 15% + Other 27%)
19,649<br>(21,072)	5.6<br>(5.6)	^{6}I 54% + ^{4}H 16% + Other 31%<br>(^{6}I 58% + ^{4}H 16% + Other 26%)
19,840<br>(21,234)	5.6<br>(5.6)	^{6}I 55% + ^{4}H 16% + Other 29%<br>(^{6}I 60% + ^{4}H 16% + Other 25%)
19,980<br>(21,270)	5.5<br>(5.6)	^{6}I 56% + ^{4}H 16% + Other 27%<br>(^{6}I 60% + ^{4}H 16% + Other 25%)
20,070<br>(21,392)	5.6<br>(5.6)	^{6}I 58% + ^{4}H 16% + Other 26%<br>(^{6}I 61% + ^{4}H 16% + Other 23%)
20,146<br>(21,476)	5.6<br>(5.6)	^{6}I 59% + ^{4}H 16% + Other 25%<br>(^{6}I 63% + ^{4}H 16% + Other 21%)
20,449<br>(21,868)	6.5<br>(6.4)	^{6}I 60% + ^{4}H 8% + ^{4}K 8% + Other 25%<br>(^{6}I 64% + ^{4}H 8% + ^{4}K 7% + Other 21%)
20,629<br>(22,023)	6.4<br>(6.5)	^{6}I 58% + ^{4}H 8% + ^{4}K 7% + Other 27%<br>(^{6}I 63% + ^{4}H 8% + ^{4}K 7% + Other 22%)
20,659<br>(22,039)	6.3<br>(6.5)	^{6}I 57% + ^{4}H 8% + ^{4}K 7% + Other 28%<br>(^{6}I 63% + ^{4}H 8% + ^{4}K 7% + Other 21%)
20,735<br>(22,101)	6.4<br>(6.5)	^{6}I 61% + ^{4}H 9% + ^{4}K 8% + Other 23%<br>(^{6}I 65% + ^{4}H 9% + ^{4}K 7% + Other 19%)
20,767<br>(22,128)	6.3<br>(6.5)	^{6}I 56% + ^{4}H 9% + ^{4}K 7% + Other 28%<br>(^{6}I 64% + ^{4}H 9% + ^{4}K 7% + Other 20%)
20,799<br>(22,136)	6.4<br>(6.4)	^{6}I 58% + ^{4}H 9% + ^{4}K 7% + Other 26%<br>(^{6}I 62% + ^{4}H 8% + ^{4}K 7% + Other 23%)
20,818<br>(22,176)	6.4<br>(6.4)	^{6}I 58% + ^{4}H 9% + ^{4}K 7% + Other 25%<br>(^{6}I 62% + ^{4}H 9% + ^{4}K 7% + Other 21%)
20,906<br>(22,271)	5.9<br>(6.2)	^{6}I 48% + ^{6}D 13% + ^{6}F 6% + ^{4}H 6% + ^{4}K 6% + Other 20%<br>(^{6}I 51% + ^{6}D 14% + ^{6}F 6% + ^{4}K 8% + Other 21%)
20,968<br>(22,289)	5.8<br>(6.2)	^{6}I 44% + ^{6}D 17% + ^{6}F 8% + ^{4}H 5% + ^{4}K 6% + Other 20%<br>(^{6}I 50% + ^{6}D 15% + ^{6}F 6% + ^{4}K 8% + Other 21%)

Structural Metrics

Table S38. Average key bond lengths (Å) and angle (°) for each of the optimized **tetra-arsine oxide** f^7 complexes.

	$[Gd^{III}(AsOPh_3)_4(NO_3)_2]^+$	$[Tb^{IV}(AsOPh_3)_4(NO_3)_2]^{2+}$	$[Cm^{III}(AsOPh_3)_4(NO_3)_2]^+$	$[Bk^{IV}(AsOPh_3)_4(NO_3)_2]^{2+}$
M–O (As)	2.285	2.172	2.328	2.207
M–O (NO_3)	2.542	2.471	2.596	2.498
As–O	1.693	1.716	1.695	1.720
M–O–As	141.0	146.5	139.5	145.8

Table S39. Average key bond length (Å) for each of the optimized **hexahalide** f^7 complexes. Experimental values are shown in brackets below, when available.

**M–X**	$[Gd^{III}X_6]^{3-}$	$[Tb^{IV}X_6]^{2-}$	$[Cm^{III}X_6]^{3-}$	$[Bk^{IV}X_6]^{2-}$
X = F	2.289	2.094	2.344	2.148
X = Cl	2.758<br>[2.673][a]	2.563	2.796	2.592
X = Br	2.910	2.712	2.947	2.741

a. See ref [41]

Table S40. Average key bond length (Å) for each of the optimized **nona-aquo** f^7 complexes. Experimental values are shown in brackets below, when available.

	$[Gd^{III}(H_2O)_9]^{3+}$	$[Tb^{IV}(H_2O)_9]^{4+}$	$[Cm^{III}(H_2O)_9]^{3+}$	$[Bk^{IV}(H_2O)_9]^{4+}$
M–O	2.476<br>[2.443][a]	2.354	2.495<br>[2.490][b]	2.323

a. See ref 40
b. See ref 37

Table S41. Cm–O bond lengths (Å) of $\mathbf{[Cm^{III}(H_2O)_9]^{3+}}$ optimized in the gas phase (gas), modeling water as the solvent with COSMO (water), and modeling acetonitrile as the solvent with COSMO (MeCN). All calculations were performed using the PBE-D3(BJ)/TZP level of theory in the ADF program.

	Gas	Water	MeCN	std deviation
O2	2.541	2.489	2.493	0.029
O3	2.519	2.473	2.472	0.027
O7	2.519	2.474	2.474	0.026
O8	2.520	2.472	2.475	0.027
O9	2.536	2.484	2.485	0.030
O10	2.519	2.469	2.472	0.028

O11	2.514	2.469	2.468	0.026
O12	2.535	2.482	2.485	0.030
O13	2.521	2.476	2.474	0.027
Average	2.525	2.476	2.478	0.028

Table S42. Cm–F bond lengths (Å) of **$[Cm^{III}F_6]^{3-}$** optimized in the gas phase (gas), modeling water as the solvent with COSMO (water), and modeling acetonitrile as the solvent with COSMO (MeCN). All calculations were performed using the PBE-D3(BJ)/TZP level of theory in the ADF program.

	Gas	Water	MeCN	std deviation
F2	2.364	2.306	2.306	0.033
F3	2.365	2.306	2.306	0.034
F4	2.365	2.306	2.305	0.034
F5	2.364	2.306	2.305	0.034
F6	2.364	2.306	2.305	0.034
F7	2.365	2.306	2.305	0.034
Average	2.365	2.306	2.305	0.034

Table S43. Cm–Cl bond lengths (Å) of **$[Cm^{III}Cl_6]^{3-}$** optimized in the gas phase (gas), modeling water as the solvent with COSMO (water), and modeling acetonitrile as the solvent with COSMO (MeCN). All calculations were performed using the PBE-D3(BJ)/TZP level of theory in the ADF program.

	Gas	Water	MeCN	std deviation
Cl2	2.810	2.734	2.734	0.044
Cl3	2.810	2.730	2.730	0.046
Cl4	2.810	2.728	2.728	0.047
Cl5	2.810	2.737	2.736	0.042
Cl6	2.810	2.734	2.734	0.044
Cl7	2.810	2.730	2.730	0.046
Average	2.810	2.732	2.732	0.045

Table S44. Cm–Br bond lengths (Å) of $[Cm^{III}Br_6]^{3-}$ optimized in the gas phase (gas), modeling water as the solvent with COSMO (water), and modeling acetonitrile as the solvent with COSMO (MeCN). All calculations were performed using the PBE-D3(BJ)/TZP level of theory in the ADF program.

	Gas	Water	MeCN	std deviation
Br2	2.951	2.876	2.875	0.044
Br3	2.951	2.873	2.873	0.045
Br4	2.952	2.873	2.873	0.046
Br5	2.952	2.876	2.875	0.044
Br6	2.953	2.874	2.874	0.046
Br7	2.953	2.874	2.874	0.046
Average	2.952	2.874	2.874	0.045

Optimized Geometries

Table S45. Optimized coordinates of $[Gd^{III}(AsOPh_3)_4(NO_3)_2]^+$.

Atom	x	y	z
64	0.029611	5.917232	4.778321
33	-1.926568	9.119875	4.413118
33	-3.213496	4.066008	4.337122
33	2.761545	3.501994	3.941660
33	2.727930	7.847101	6.512640
8	-1.615054	7.457461	4.482670
8	-1.541536	4.324975	4.270001
8	2.051999	4.977512	4.365411
8	1.133642	7.370154	6.184646
8	-0.006247	5.777799	2.266714
8	1.129379	7.463101	3.078527
8	1.048591	7.153441	0.894023
8	-1.219527	5.681798	6.995605
8	0.328854	4.181415	6.620148
8	-0.882936	4.067771	8.465762
7	0.736110	6.813699	2.050184
7	-0.602121	4.630497	7.388823
6	-0.540399	10.152115	3.532253
6	0.489524	10.709758	4.295396
1	0.497204	10.605201	5.379855
6	1.514598	11.408058	3.652799
1	2.316635	11.852954	4.244342
6	1.509037	11.534682	2.259716

1	2.311681	12.080242	1.760054
6	0.474753	10.971758	1.504800
1	0.469539	11.073737	0.418407
6	-0.559276	10.279365	2.139006
1	-1.369494	9.849289	1.547550
1	1.446873	5.904922	8.322736
6	4.934573	8.328785	2.972433
1	4.902069	8.982289	2.099090
6	5.923673	7.345398	3.078831
6	4.033573	7.629771	5.095960
6	5.005638	6.628567	5.201342
1	3.196554	9.228379	3.887315
6	3.979472	8.474641	3.981299
1	6.666708	7.234618	2.287451
6	5.954370	6.491513	4.185468
1	6.714355	5.711954	4.258694
6	-2.168343	9.828192	6.194405
6	-2.235663	11.211110	6.404364
1	-2.135624	11.911678	5.573057
6	-2.418351	11.690891	7.704015
1	-2.473858	12.766216	7.880745
6	-2.520184	10.793995	8.773212
1	-2.658296	11.173413	9.787181
6	-2.443081	9.415163	8.549728
1	-2.519427	8.719403	9.387245
6	-2.268682	8.921302	7.254104
1	-2.200120	7.848471	7.070741
6	-3.537890	9.299498	3.347695
6	-3.721492	8.373637	2.311985
1	-2.985440	7.584890	2.142642
6	-4.872627	8.449905	1.524453
1	-5.025387	7.726767	0.722146
6	-5.828171	9.440536	1.776645
1	-6.730348	9.493699	1.164987
6	-5.635822	10.359310	2.813730
1	-6.385675	11.126473	3.012674
6	-4.484888	10.294770	3.605880
1	-4.344268	11.006172	4.421709
6	-4.173536	5.193791	5.594022
6	-4.319343	4.774154	6.920542
1	-3.941347	3.802521	7.243567
6	-4.953370	5.617872	7.836353
1	-5.070711	5.299127	8.873392
6	-5.433223	6.865739	7.425517
1	-5.923607	7.523228	8.145450
6	-5.288131	7.273818	6.095518

1	-5.661985	8.247628	5.774229
6	-4.656508	6.437001	5.173522
1	-4.538262	6.756123	4.138932
6	-3.980914	4.380794	2.586977
6	-5.369038	4.448708	2.415583
1	-6.046833	4.340865	3.264598
6	-5.884782	4.680802	1.137635
1	-6.964728	4.737589	0.992728
6	-5.017910	4.849677	0.052403
1	-5.425345	5.034646	-0.943111
6	-3.632567	4.794880	0.238828
1	-2.959117	4.940767	-0.607562
6	-3.104672	4.557928	1.511412
1	-2.026089	4.539327	1.677017
6	-3.478918	2.243339	4.938517
6	-2.450345	1.653766	5.680093
1	-1.507900	2.186094	5.827436
6	-2.641616	0.371594	6.203196
1	-1.846640	-0.099474	6.783759
6	-3.841736	-0.308093	5.973938
1	-3.986139	-1.309972	6.382172
6	-4.858010	0.286548	5.217082
1	-5.790238	-0.249639	5.031034
6	-4.683078	1.572319	4.698903
1	-5.478864	2.034682	4.112048
6	1.485719	2.052710	3.802359
6	0.462133	2.172922	2.853967
1	0.381797	3.067250	2.232860
6	-0.464119	1.137947	2.721059
1	-1.271284	1.223285	1.991542
6	-0.362873	-0.000160	3.527761
1	-1.092642	-0.804755	3.423991
6	0.660066	-0.108694	4.474477
1	0.732256	-0.996051	5.105587
6	1.592576	0.923011	4.619621
1	2.393594	0.841912	5.356758
6	4.098746	3.083881	5.275544
6	5.384159	2.681130	4.901004
6	1.506356	10.151692	7.685209
6	6.345610	2.470226	5.894313
1	7.354156	2.161309	5.614268
6	6.016834	2.664696	7.239642
1	6.772398	2.505399	8.010811
6	4.726795	3.067764	7.601851
1	4.475335	3.228269	8.650978
6	3.757503	3.282696	6.619575

1	2.754963	3.619099	6.893134
6	3.581078	3.714279	2.204603
6	3.963031	2.596995	1.452535
1	3.798657	1.585009	1.828652
6	4.547948	2.793754	0.198852
1	4.850977	1.932433	-0.398589
6	4.736358	4.092257	-0.289798
1	5.188416	4.240733	-1.272214
1	4.586992	10.263527	6.180828
1	5.024659	5.957767	6.061102
6	3.584430	11.943288	7.103731
6	2.652162	9.722131	7.002532
1	4.390518	12.642432	6.875377
1	2.355082	13.430857	8.073293
1	0.693088	9.451698	7.888503
6	1.404998	11.489627	8.071332
1	0.511884	11.833762	8.594818
6	2.440731	12.384071	7.776927
6	3.697846	10.604228	6.714057
6	4.341969	5.198877	0.469191
1	4.484567	6.209371	0.082375
6	3.760934	5.014912	1.726326
1	3.442665	5.867014	2.329035
6	3.351160	6.881372	8.077725
6	4.643329	7.085399	8.576945
1	5.341896	7.757015	8.073901
6	5.036776	6.414994	9.738078
1	6.042903	6.563749	10.133110
6	4.142086	5.559836	10.392497
1	4.454353	5.039778	11.299728
6	2.850610	5.370993	9.890300
1	2.152292	4.706831	10.402326
6	2.449546	6.034929	8.727440
1	5.643919	2.546591	3.849382

Table S46. optimized coordinates for **$[Tb^{IV}(AsOPh_3)_4(NO_3)_2]^{2+}$**.

Atom	x	y	z
65	0.000000	0.000000	0.000000
33	-2.818089	-2.191717	0.998818
33	-2.423870	2.622490	-1.121805
33	2.431671	-2.744749	-0.609592
33	2.737164	2.509794	0.387785
8	-1.757038	1.191219	-0.453094
8	1.786477	-1.166306	-0.403081
8	1.209188	1.734878	0.500007

8	-1.276560	-1.710368	0.417538
8	-0.837928	0.683884	2.218459
8	0.663191	0.860997	-2.206798
8	-0.538797	-0.953280	-2.215001
8	0.817144	-0.729167	2.228661
8	0.207001	-0.130040	-4.125645
7	0.115558	-0.074017	-2.899326
6	1.135397	-4.136278	-0.272403
7	0.000000	0.000000	2.912532
6	-2.485016	-3.663667	2.202819
6	3.819149	1.869628	-1.078770
6	4.952604	1.099051	-0.794326
1	5.211199	0.843075	0.234383
6	3.022552	-2.873377	-2.437319
6	-4.432582	0.180293	1.266684
1	-4.559546	0.095956	0.187564
6	3.477165	2.209744	-2.394250
1	2.579170	2.790084	-2.609342
6	-3.374147	-2.756846	-1.773320
1	-2.319887	-2.527728	-1.926218
6	-4.324763	0.854397	-2.342595
1	-3.468785	0.185392	-2.451246
8	0.017783	0.026568	4.145240
6	3.684702	2.230254	2.045816
6	-5.002123	1.250469	1.957898
1	-5.574091	2.002830	1.412538
6	-3.699508	-0.776561	1.978526
6	-2.322904	3.642394	1.570130
1	-2.122138	2.608762	1.852250
6	-2.504459	3.995063	0.228848
6	3.675872	-2.706353	1.986660
1	2.681496	-2.442771	2.353001
6	-3.417199	-4.696656	2.340706
1	-4.326976	-4.720828	1.739147
6	-1.362298	3.268078	-2.602455
6	-3.938918	-2.669403	-0.497108
6	5.182320	-3.224185	0.133312
1	5.351645	-3.376273	-0.934093
6	3.876001	-3.041231	-5.068330
1	4.209509	-3.106749	-6.105137
6	3.137006	5.188404	-0.677317
1	3.971342	4.766292	-1.239205
6	3.908660	-2.912938	0.619730
6	-1.306002	-3.632935	2.956490
1	-0.572677	-2.838604	2.807865
6	3.247071	-4.135288	-3.001010

1	3.080174	-5.046797	-2.423868
6	-4.179126	2.112579	-1.743031
6	2.365538	4.390480	0.174073
6	-6.102425	-3.282442	-1.361744
1	-7.163125	-3.481461	-1.203961
6	-4.190304	-3.115523	-2.849110
1	-3.764257	-3.181115	-3.851448
6	-1.068292	-4.653455	3.879827
1	-0.153614	-4.642006	4.474304
6	-5.591402	0.453373	-2.771772
1	-5.717879	-0.526541	-3.233156
6	-0.755626	-6.118898	0.170496
1	-1.501759	-6.894659	0.347773
6	-2.387461	4.645003	2.541293
1	-2.240850	4.388479	3.591631
6	-5.547279	-3.385066	-2.641656
1	-6.179484	-3.667946	-3.484947
6	-5.299931	-2.917243	-0.277996
1	-5.738991	-2.817589	0.716482
6	-1.993765	-5.690309	4.031745
1	-1.801462	-6.488010	4.751129
6	-3.538162	-0.688301	3.365397
1	-2.974250	-1.443920	3.914809
6	3.214395	-1.695633	-3.165495
1	3.026993	-0.724185	-2.705731
6	-2.749228	5.318664	-0.159212
1	-2.872436	5.584367	-1.210621
6	3.682097	-4.211828	-4.326063
1	3.863106	-5.187470	-4.778987
6	3.643876	0.969092	2.651245
1	3.063119	0.157934	2.211034
6	1.744502	7.082653	-0.089204
1	1.496845	8.139622	-0.197852
6	-0.913763	-5.234468	-0.902424
1	-1.778283	-5.321401	-1.562129
6	0.033515	-4.236463	-1.132248
1	-0.084152	-3.544410	-1.967902
6	4.343005	0.772074	3.843626
1	4.316214	-0.203634	4.330426
6	4.742751	-2.830640	2.878381
1	4.574530	-2.686889	3.946785
6	-4.837443	1.356012	3.343440
1	-5.280255	2.196983	3.879585
6	6.021845	-3.143037	2.404379
1	6.851745	-3.235506	3.106606
6	-6.691071	1.300636	-2.597204

1	-7.680160	0.980112	-2.927892
6	-0.296747	4.139433	-2.346358
1	-0.102545	4.505328	-1.337776
6	-5.269284	2.969337	-1.564040
1	-5.147660	3.942069	-1.084507
6	0.988831	6.275994	0.769905
1	0.155191	6.699561	1.331167
6	-6.531883	2.553274	-1.996361
1	-7.392212	3.209044	-1.855594
6	1.308762	-5.018899	0.798520
1	2.176889	-4.943073	1.454406
6	3.641190	-1.788947	-4.492205
1	3.789242	-0.879597	-5.076231
6	5.762493	0.670781	-1.848883
1	6.648258	0.069474	-1.639432
6	1.294466	4.920833	0.906241
1	0.698205	4.283033	1.561004
6	4.407319	3.290298	2.605067
1	4.424336	4.271965	2.128207
6	0.352779	-6.015623	1.014175
1	0.475783	-6.708105	1.847594
6	-4.113538	0.387567	4.045991
1	-3.993688	0.467940	5.127283
6	-3.160664	-5.716645	3.260618
1	-3.876318	-6.532050	3.374441
6	5.443107	1.021750	-3.163833
1	6.084484	0.693393	-3.983096
6	6.242681	-3.335783	1.037554
1	7.241665	-3.576065	0.670879
6	4.303000	1.785537	-3.436838
1	4.053581	2.052064	-4.465151
6	-0.798581	3.200942	-4.943077
1	-0.992090	2.836841	-5.952869
6	5.070795	1.821685	4.413970
1	5.612310	1.661046	5.347658
6	-2.638664	5.970295	2.170307
1	-2.689524	6.747533	2.934780
6	2.816103	6.543551	-0.807057
1	3.405256	7.175562	-1.472945
6	-1.628395	2.799778	-3.893765
1	-2.470650	2.133446	-4.088403
6	5.103674	3.076894	3.797743
1	5.667515	3.895693	4.246904
6	0.518140	4.541304	-3.406286
1	1.344884	5.228932	-3.220572
6	-2.820623	6.307480	0.824837

1	-3.010892	7.342182	0.536753
6	0.272430	4.065922	-4.699081
1	0.916474	4.377804	-5.522959

Table S47. optimized coordinates for **[Cm^{III}(AsOPh$_3$)$_4$(NO$_3$)$_2$]$^+$**.

Atom	x	y	z
96	0.075889	5.798202	4.676630
33	-1.916205	9.013872	4.328616
33	-3.353688	4.208053	4.432329
33	2.953501	3.523340	3.843424
33	2.661600	7.828543	6.528239
8	-1.619263	7.345317	4.340232
8	-1.670729	4.309496	4.260864
8	2.203127	4.983497	4.259661
8	1.086854	7.383240	6.076302
8	0.049243	5.548071	2.125954
8	1.102602	7.335679	2.835458
8	1.013907	6.906251	0.672264
8	-1.017709	5.391441	7.021060
8	0.427500	3.873169	6.379528
8	-0.741711	3.563369	8.229001
7	0.733300	6.612985	1.850005
7	-0.454107	4.258112	7.234180
6	-0.525396	10.065263	3.478277
6	0.486213	10.631614	4.259283
1	0.480318	10.519667	5.343170
6	1.509013	11.350196	3.634738
1	2.295895	11.804034	4.239722
6	1.520702	11.485266	2.242560
1	2.321445	12.046336	1.757364
6	0.506020	10.910612	1.469678
1	0.514830	11.019030	0.383915
6	-0.526308	10.199770	2.085471
1	-1.321761	9.760752	1.480524
1	1.233089	5.904413	8.235736
6	4.923787	8.335600	3.034696
1	4.874640	8.969029	2.147409
6	5.955682	7.401758	3.176602
6	4.021081	7.637541	5.158764
6	5.036942	6.686216	5.300068
1	3.131655	9.170998	3.903261
6	3.947273	8.456661	4.025687
1	6.715836	7.309551	2.399154
6	6.007520	6.573451	4.301492
1	6.801059	5.831652	4.403179

6	-2.144470	9.649820	6.139157
6	-2.238939	11.020274	6.410950
1	-2.164074	11.759268	5.610780
6	-2.413623	11.437804	7.732950
1	-2.490581	12.502799	7.957734
6	-2.480110	10.492839	8.763006
1	-2.612039	10.824298	9.794423
6	-2.376960	9.127372	8.477573
1	-2.427967	8.392577	9.282907
6	-2.211663	8.696294	7.159090
1	-2.130440	7.633665	6.927697
6	-3.533081	9.238585	3.280511
6	-3.742160	8.325297	2.239443
1	-3.020602	7.527058	2.055994
6	-4.902712	8.422570	1.468366
1	-5.074749	7.708212	0.662134
6	-5.843028	9.421314	1.743239
1	-6.753341	9.490167	1.145241
6	-5.625096	10.327946	2.786091
1	-6.363344	11.101594	3.002868
6	-4.464081	10.242661	3.561228
1	-4.305327	10.943593	4.382633
6	-4.207403	5.440033	5.667399
6	-4.175677	5.149130	7.036046
1	-3.682875	4.247740	7.403700
6	-4.779789	6.032652	7.933432
1	-4.757067	5.815928	9.002857
6	-5.408228	7.190415	7.463019
1	-5.876497	7.878798	8.168550
6	-5.436482	7.471214	6.093751
1	-5.922799	8.376587	5.727041
6	-4.834087	6.594738	5.188146
1	-4.852604	6.814111	4.120774
6	-4.149460	4.473005	2.684252
6	-5.538670	4.498768	2.514217
1	-6.213975	4.388671	3.364806
6	-6.062471	4.683874	1.231999
1	-7.143814	4.707890	1.088216
6	-5.202450	4.843571	0.139724
1	-5.616559	4.990405	-0.859409
6	-3.815970	4.824121	0.323408
1	-3.147624	4.956901	-0.529316
6	-3.279943	4.636937	1.601071
1	-2.200141	4.635524	1.763620
6	-3.703232	2.429094	5.124622
6	-2.597551	1.634120	5.440791

1	-1.589388	2.016069	5.270801
6	-2.808786	0.357355	5.970413
1	-1.952646	-0.271137	6.222539
6	-4.110224	-0.111285	6.174322
1	-4.271520	-1.108772	6.587175
6	-5.209873	0.694140	5.853784
1	-6.224641	0.327207	6.015913
6	-5.010972	1.973945	5.329958
1	-5.871564	2.602917	5.093620
6	1.705024	2.048745	3.714834
6	0.664079	2.151669	2.782922
1	0.562948	3.039711	2.154994
6	-0.253358	1.105788	2.673908
1	-1.073539	1.177451	1.957685
6	-0.125518	-0.025954	3.486534
1	-0.846074	-0.840783	3.398202
6	0.914510	-0.116160	4.416444
1	1.008875	-0.998624	5.051650
6	1.837998	0.926755	4.537976
1	2.650677	0.860728	5.263629
6	4.287791	3.152752	5.195446
6	5.606054	2.851783	4.841199
6	1.437456	10.127658	7.722627
6	6.563745	2.706909	5.850004
1	7.597793	2.479369	5.585462
6	6.198821	2.865162	7.190805
1	6.951696	2.759467	7.973827
6	4.874937	3.162660	7.533257
1	4.593437	3.293903	8.578838
6	3.909601	3.311305	6.535303
1	2.879611	3.567235	6.793784
6	3.786330	3.744956	2.114828
6	4.202954	2.632330	1.374577
1	4.053867	1.618906	1.753219
6	4.804363	2.835769	0.129778
1	5.133862	1.978342	-0.459131
6	4.977533	4.135851	-0.360085
1	5.444993	4.289434	-1.334497
1	4.527618	10.234733	6.238249
1	5.071938	6.030358	6.170516
6	3.523929	11.915568	7.157793
6	2.586887	9.696456	7.047239
1	4.333046	12.613077	6.935283
1	2.290932	13.404566	8.120696
1	0.619080	9.431990	7.919886
6	1.337081	11.465459	8.109347

1	0.441348	11.811040	8.627058
6	2.376749	12.358004	7.823605
6	3.636356	10.576870	6.766857
6	4.548738	5.237707	0.387295
1	4.680493	6.249062	-0.001308
6	3.947576	5.047338	1.634029
1	3.600293	5.894541	2.227664
6	3.181744	6.812481	8.095607
6	4.454302	6.967646	8.658043
1	5.190886	7.636946	8.209228
6	4.777216	6.250758	9.812656
1	5.767738	6.360290	10.256968
6	3.832969	5.398122	10.396419
1	4.091653	4.840130	11.298067
6	2.559630	5.262572	9.833954
1	1.821507	4.604396	10.295400
6	2.226799	5.975880	8.678743
1	5.893385	2.747692	3.793377

Table S48. Optimized coordinates for **[Bk^{IV}(AsOPh$_3$)$_4$(NO$_3$)$_2$]$^{2+}$**.

Atom	x	y	z
97	0.036339	6.016702	4.835281
33	-1.917984	9.220576	4.390695
33	-3.147385	4.116152	4.374876
33	2.622632	3.434803	3.913605
33	2.832300	7.803285	6.552641
8	-1.507573	7.559797	4.561704
8	-1.463658	4.466164	4.334931
8	1.871519	4.914928	4.359036
8	1.221180	7.312771	6.197633
8	-0.068262	6.000137	2.351566
8	1.204312	7.536612	3.235617
8	1.070573	7.366221	1.037881
8	-1.233607	5.891881	6.980780
8	0.290797	4.355717	6.702322
8	-0.937580	4.355321	8.540604
7	0.751295	6.985370	2.163434
7	-0.639595	4.849207	7.451183
6	-0.521270	10.209204	3.496285
6	0.525952	10.732142	4.263471
1	0.529726	10.629662	5.348601
6	1.571578	11.395826	3.619711
1	2.386793	11.817458	4.210006
6	1.567690	11.523177	2.226327
1	2.387968	12.041217	1.726572

6	0.513510	11.000124	1.471023
1	0.509148	11.107473	0.385539
6	-0.543380	10.341185	2.103393
1	-1.371270	9.946014	1.512728
1	1.415818	6.105135	8.535335
6	5.054474	8.304131	3.034837
1	5.069211	9.001417	2.195892
6	5.975921	7.253341	3.093929
6	4.106861	7.563951	5.122572
6	5.009239	6.494229	5.179762
1	3.383152	9.277082	3.997151
6	4.109881	8.465314	4.050449
1	6.713519	7.133532	2.299175
6	5.948766	6.346613	4.157673
1	6.657896	5.518564	4.193979
6	-2.190713	9.968952	6.145777
6	-2.255963	11.359693	6.298925
1	-2.148231	12.027258	5.442060
6	-2.449728	11.887665	7.577811
1	-2.503794	12.968740	7.712379
6	-2.563411	11.032274	8.679112
1	-2.708278	11.450244	9.676601
6	-2.490949	9.645527	8.510478
1	-2.577226	8.982717	9.373047
6	-2.307732	9.101307	7.236831
1	-2.247562	8.021740	7.099119
6	-3.525571	9.226568	3.323731
6	-3.595048	8.349599	2.232357
1	-2.763198	7.683195	1.994980
6	-4.764064	8.313373	1.469840
1	-4.831474	7.632450	0.620931
6	-5.845189	9.136492	1.804377
1	-6.759695	9.097720	1.210790
6	-5.763703	10.004042	2.897791
1	-6.610873	10.639207	3.160136
6	-4.596983	10.056938	3.666277
1	-4.536691	10.727129	4.525497
6	-4.113886	5.204809	5.646313
6	-4.293684	4.732747	6.951275
1	-3.937635	3.743641	7.244269
6	-4.942937	5.547483	7.881893
1	-5.088794	5.190684	8.902315
6	-5.405015	6.812321	7.504920
1	-5.910079	7.445483	8.236110
6	-5.229271	7.268624	6.194107
1	-5.598074	8.252518	5.900347

6	-4.580545	6.464969	5.255405
1	-4.442919	6.817247	4.233612
6	-3.883285	4.410634	2.617136
6	-5.272153	4.495281	2.457483
1	-5.943343	4.430727	3.315814
6	-5.793093	4.682265	1.174715
1	-6.872780	4.751609	1.036166
6	-4.932170	4.789577	0.077569
1	-5.344290	4.941054	-0.921398
6	-3.546308	4.714329	0.253304
1	-2.877975	4.808770	-0.603881
6	-3.011006	4.519424	1.529038
1	-1.931852	4.477050	1.678821
6	-3.295900	2.275306	4.937602
6	-2.328463	1.762105	5.808218
1	-1.458776	2.359100	6.088858
6	-2.477091	0.457761	6.286060
1	-1.729457	0.043010	6.963718
6	-3.570251	-0.316738	5.885586
1	-3.679867	-1.335955	6.259604
6	-4.520929	0.203817	5.001287
1	-5.367227	-0.405769	4.681504
6	-4.391476	1.510454	4.523655
1	-5.134143	1.916055	3.834337
6	1.329049	2.015102	3.756032
6	0.328613	2.150800	2.784276
1	0.278220	3.042108	2.156069
6	-0.604543	1.125423	2.629711
1	-1.392124	1.217571	1.880242
6	-0.527031	-0.017266	3.433125
1	-1.259837	-0.815871	3.309230
6	0.476677	-0.141020	4.398052
1	0.530450	-1.033314	5.023157
6	1.414504	0.881145	4.570219
1	2.201387	0.787874	5.320503
6	3.943140	3.068754	5.269541
6	5.253916	2.756807	4.894941
6	1.509864	10.070914	7.676727
6	6.218824	2.591756	5.892665
1	7.246860	2.354047	5.615389
6	5.868216	2.741777	7.238183
1	6.627422	2.620458	8.012354
6	4.551749	3.049453	7.598061
1	4.283120	3.171708	8.647753
6	3.575982	3.217647	6.613685
1	2.553683	3.481411	6.891294

6	3.429935	3.727399	2.190376
6	3.765256	2.632347	1.385205
1	3.570028	1.610249	1.715302
6	4.347007	2.869579	0.137447
1	4.615810	2.027354	-0.501636
6	4.573848	4.182162	-0.291837
1	5.020928	4.361312	-1.271028
1	4.622969	10.257248	6.238276
1	4.986318	5.784460	6.007507
6	3.553925	11.913563	7.127034
6	2.680671	9.672599	7.017679
1	4.346881	12.631607	6.913717
1	2.267009	13.371477	8.064721
1	0.712694	9.352225	7.875150
6	1.367067	11.407468	8.053171
1	0.456659	11.730050	8.559866
6	2.385229	12.326544	7.774080
6	3.711749	10.577098	6.745818
6	4.227664	5.265436	0.522244
1	4.403023	6.287115	0.182069
6	3.654532	5.043623	1.776140
1	3.383249	5.879736	2.421977
6	3.412435	6.814435	8.111347
6	4.751434	6.875316	8.516722
1	5.492078	7.433070	7.941007
6	5.135785	6.204849	9.680391
1	6.177630	6.239303	10.002071
6	4.189269	5.495509	10.427792
1	4.495868	4.973882	11.335793
6	2.852126	5.454978	10.020213
1	2.113374	4.907807	10.607914
6	2.456084	6.119009	8.856558
1	5.528233	2.657765	3.843340

Table S49. Optimized coordinates of [**Gd**^{III}**F**$_6$]$^{3-}$.

Atom	x	y	z
64	-0.000006	0.000000	-0.000005
9	-0.000007	2.289286	0.000008
9	0.000000	-2.289307	0.000010
9	2.289295	-0.000008	0.000007
9	-2.289287	-0.000001	0.000002
9	0.000000	0.000016	-2.289297
9	0.000005	0.000014	2.289276

Table S50. Optimized coordinates of [$\mathbf{Tb^{IV}F_6}$]$^{2-}$.

Atom	x	y	z
65	0.000001	-0.000006	0.000006
9	-0.000001	0.000001	2.093656
9	-0.000001	0.000003	-2.093641
9	-2.093653	-0.000006	0.000002
9	2.093646	0.000002	-0.000020
9	-0.000010	2.093640	0.000011
9	0.000018	-2.093635	-0.000013

Table S51. Optimized coordinates of [$\mathbf{Cm^{III}F_6}$]$^{3-}$.

Atom	x	y	z
96	0.000002	0.000001	0.000002
9	-0.000025	2.344018	-0.000009
9	-0.000022	-2.344013	-0.000009
9	2.343994	0.000004	0.000008
9	-2.344001	0.000007	0.000005
9	0.000024	-0.000008	-2.343991
9	0.000027	-0.000008	2.343994

Table S52. Optimized coordinates of [$\mathbf{Bk^{IV}F_6}$]$^{2-}$.

Atom	x	y	z
97	0.000000	0.000002	0.000001
9	-0.000015	2.147916	-0.000001
9	-0.000016	-2.147945	0.000000
9	2.147913	0.000000	0.000009
9	-2.147886	-0.000001	0.000014
9	0.000004	0.000015	-2.147897
9	-0.000001	0.000013	2.147874

Table S53. Optimized coordinates of [$\mathbf{Gd^{III}Cl_6}$]$^{3-}$.

Atom	x	y	z
64	0.000000	0.000004	-0.000006
17	-0.000016	2.757869	-0.000028
17	-0.000022	-2.757877	-0.000026
17	2.757886	-0.000026	0.000036
17	-2.757858	-0.000032	0.000033
17	0.000004	0.000032	-2.757863
17	0.000007	0.000031	2.757854

Table S54. Optimized coordinates of [$\mathbf{Tb^{IV}Cl_6}$]$^{2-}$.

Atom	x	y	z
65	0.000002	-0.000004	0.000006
17	-0.000080	-0.000013	2.562664
17	0.000123	0.000019	-2.562660
17	-2.562800	0.000265	0.000102
17	2.562743	-0.000249	-0.000101
17	-0.000251	2.562620	-0.000022
17	0.000263	-2.562637	0.000010

Table S55. Optimized coordinates of [$\mathbf{Cm^{III}Cl_6}$]$^{3-}$.

Atom	x	y	z
96	0.000004	-0.000006	0.000002
17	-0.000010	2.795601	0.000048
17	-0.000009	-2.795610	0.000046
17	2.795606	0.000002	-0.000053
17	-2.795597	0.000003	-0.000046
17	0.000006	0.000005	-2.795586
17	0.000000	0.000006	2.795589

Table S56. Optimized coordinates of [$\mathbf{Bk^{IV}Cl_6}$]$^{2-}$.

Atom	x	y	z
97	0.000000	-0.000006	0.000007
17	-0.000010	2.592451	0.000013
17	0.000017	-2.592465	-0.000004
17	2.592454	-0.000001	-0.000011
17	-2.592451	0.000025	-0.000012
17	-0.000005	-0.000011	-2.592444
17	-0.000004	0.000006	2.592451

Table S57. Optimized coordinates of [$\mathbf{Gd^{III}Br_6}$]$^{3-}$.

Atom	x	y	z
64	0.000005	0.000001	0.000003
35	0.000063	2.910038	-0.000034
35	0.000049	-2.910027	-0.000043
35	2.910026	-0.000037	0.000008
35	-2.910029	-0.000051	0.000044
35	-0.000039	0.000034	-2.910015
35	-0.000074	0.000043	2.910037

Table S58. Optimized coordinates of [$\mathbf{Tb^{IV}Br_6}$]$^{2-}$.

Atom	x	y	z
65	0.000000	0.000000	0.000000
35	0.000001	2.712317	0.000002
35	-0.000002	-2.712318	-0.000002
35	2.712319	-0.000001	0.000000
35	-2.712317	-0.000005	0.000002
35	-0.000001	0.000006	2.712317
35	0.000001	0.000002	-2.712318

Table S59. Optimized coordinates of [$\mathbf{Cm^{III}Br_6}$]$^{3-}$.

Atom	x	y	z
96	0.000000	0.000021	-0.000005
35	0.000004	2.947312	-0.000053
35	0.000022	-2.947264	-0.000029
35	2.947308	0.000028	0.000054
35	-2.947302	0.000046	0.000042
35	-0.000022	-0.000059	-2.947294
35	-0.000010	-0.000084	2.947285

Table S60. Optimized coordinates of [$\mathbf{Bk^{IV}Br_6}$]$^{2-}$.

Atom	x	y	z
97	0.000009	-0.000005	-0.000004
35	-0.000005	2.740964	-0.000010
35	0.000009	-2.740977	-0.000010
35	2.740966	0.000017	0.000023
35	-2.740939	0.000031	0.000026
35	-0.000018	-0.000015	-2.740975
35	-0.000021	-0.000015	2.740950

Table S61. Optimized coordinates of [$\mathbf{Gd^{III}(H_2O)_9}$]$^{3+}$.

Atom	x	y	z
64	-0.000469	7.948563	1.831798
8	1.474918	5.961621	1.832001
1	1.654178	5.398834	2.596632
8	-0.813368	6.466273	3.635658
1	-0.343680	6.252963	4.452750
1	-1.548088	5.839607	3.586342
8	1.101029	10.175597	1.831782
8	-2.479208	7.703727	1.831881
1	1.655279	5.399107	1.067428

1	1.519975	10.590049	2.597264
1	1.520016	10.589831	1.066207
1	-3.053028	7.834492	2.597871
1	-3.054508	7.838553	1.067700
8	1.645142	7.985490	3.704670
8	-0.883509	9.430860	3.623079
8	-0.812959	6.466826	0.028111
8	1.645609	7.985520	-0.040787
8	-0.883825	9.430648	0.040495
1	1.565203	8.493720	4.522663
1	-1.355271	9.183848	4.429615
1	-0.342677	6.251845	-0.788203
1	1.565671	8.493320	-0.859052
1	-1.355143	9.183472	-0.766255
1	2.564619	7.687161	3.674531
1	-1.000942	10.386297	3.534953
1	-1.549663	5.842438	0.076580
1	2.565263	7.687780	-0.010198
1	-1.001257	10.386109	0.128383

Table S62. Optimized coordinates of [$\mathbf{Tb^{IV}(H_2O)_9}$]$^{4+}$.

Atom	x	y	z
65	0.001904	7.989910	1.864825
8	-2.392981	7.914777	1.901149
8	-0.751717	6.308933	3.298047
1	-2.956895	7.351010	2.458431
1	-1.176040	5.458345	3.087653
1	-0.619263	6.311337	4.262360
8	1.678931	6.360574	1.859797
8	1.573582	9.718432	1.822408
8	1.232826	8.038759	3.912109
8	-0.841184	9.637780	3.284383
8	-0.778986	6.296908	0.461550
8	1.176832	8.011446	-0.218910
8	-0.898787	9.608453	0.448624
1	-3.005646	8.440302	1.358168
1	1.727932	5.528844	2.363269
1	2.454630	6.364245	1.271850
1	2.365795	9.760175	2.386483
1	1.562196	10.548780	1.314344
1	2.021695	7.514794	4.134870
1	-0.448034	10.488813	3.547178
1	-1.677762	6.268594	0.089063
1	1.927001	8.578079	-0.469204
1	-1.363099	10.435331	0.669067

1	1.053729	8.586654	4.695889
1	-1.728576	9.616680	3.683843
1	-0.350753	5.464945	0.192597
1	1.006800	7.446619	-0.992470
1	-0.794818	9.608059	-0.519177

Table S63. Optimized coordinates of [$\mathbf{Cm^{III}(H_2O)_9}$]$^{3+}$.

Atom	x	y	z
96	0.004287	7.979870	1.862060
8	-2.508827	7.889211	1.873561
8	-0.793241	6.180601	3.379443
1	-3.069210	7.361672	2.457965
1	-1.227976	5.351840	3.138784
1	-0.670694	6.146332	4.337533
8	1.777943	6.235629	1.871674
8	1.646614	9.854973	1.821277
8	1.295018	8.045970	4.008426
8	-0.893531	9.754508	3.360049
8	-0.818256	6.160225	0.392148
8	1.246823	8.015078	-0.315406
8	-0.942429	9.733841	0.371029
1	-3.112605	8.419213	1.336551
1	1.807321	5.425596	2.397389
1	2.550007	6.201650	1.291487
1	2.430146	9.930663	2.381911
1	1.617359	10.664963	1.295541
1	2.083045	7.532328	4.229179
1	-0.473239	10.587726	3.610580
1	-1.727758	6.109191	0.069322
1	1.991898	8.586238	-0.544375
1	-1.430042	10.532443	0.612118
1	1.107449	8.591244	4.783731
1	-1.771311	9.770704	3.763780
1	-0.376979	5.366223	0.062814
1	1.093027	7.460568	-1.091533
1	-0.835526	9.769079	-0.588841

Table S64. Optimized coordinates of [$\mathbf{Bk^{IV}(H_2O)_9}$]$^{4+}$.

Atom	x	y	z
97	0.001698	7.990421	1.864483
8	-2.366266	7.915261	1.898920
8	-0.748077	6.335792	3.276582
1	-2.935387	7.353821	2.452929

1	-1.165493	5.481816	3.070115
1	-0.622263	6.339587	4.241160
8	1.658614	6.385546	1.865652
8	1.554714	9.692150	1.816572
8	1.219004	8.038655	3.890369
8	-0.825650	9.612027	3.270381
8	-0.764048	6.322558	0.476872
8	1.161887	8.011521	-0.196652
8	-0.891627	9.583495	0.471021
1	-2.982762	8.437319	1.357370
1	1.707107	5.550607	2.362357
1	2.437887	6.389496	1.283629
1	2.350722	9.733231	2.374043
1	1.543120	10.525859	1.315450
1	2.007943	7.517344	4.118344
1	-0.438657	10.465898	3.529996
1	-1.660123	6.294442	0.099803
1	1.912598	8.574418	-0.453218
1	-1.351412	10.412737	0.688439
1	1.044672	8.583602	4.677082
1	-1.709704	9.590003	3.675488
1	-0.341437	5.487942	0.210128
1	0.995226	7.449055	-0.972498
1	-0.792973	9.582975	-0.496619

References

1. Neese, F. The ORCA program system. *WIREs Comput. Mol. Sci.* **2**, 73–78 (2012).

2. Neese, F. Software Update: The ORCA Program System—Version 6.0. *WIREs Comput. Mol. Sci.* **15**, e70019 (2025).

3. Perdew, J. P., Ernzerhof, M. & Burke, K. Rationale for mixing exact exchange with density functional approximations. *J. Chem. Phys.* **105**, 9982–9985 (1996).

4. Pantazis, D. A. & Neese, F. All-Electron Scalar Relativistic Basis Sets for the Lanthanides. *J. Chem. Theory Comput.* **5**, 2229–2238 (2009).

5. Pantazis, D. A. & Neese, F. All-Electron Scalar Relativistic Basis Sets for the Actinides. *J. Chem. Theory Comput.* **7**, 677–684 (2011).

6. Weigend, F. & Ahlrichs, R. Balanced basis sets of split valence, triple zeta valence and quadruple zeta valence quality for H to Rn: Design and assessment of accuracy. *Phys. Chem. Chem. Phys.* **7**, 3297–3305 (2005).

7. Hess, B. A. Relativistic electronic-structure calculations employing a two-component no-pair formalism with external-field projection operators. *Phys. Rev. A* **33**, 3742–3748 (1986).

8. Jansen, G. & Hess, B. A. Revision of the Douglas-Kroll transformation. *Phys. Rev. A* **39**, 6016–6017 (1989).

9. Wolf, A., Reiher, M. & Hess, B. A. The generalized Douglas–Kroll transformation. *J. Chem. Phys.* **117**, 9215–9226 (2002).

10. Stoychev, G. L., Auer, A. A. & Neese, F. Automatic Generation of Auxiliary Basis Sets. *J. Chem. Theory Comput.* **13**, 554–562 (2017).

11. Helmich-Paris, B., De Souza, B., Neese, F. & Izsák, R. An improved chain of spheres for exchange algorithm. *J. Chem. Phys.* **155**, 104109 (2021).

12. Neese, F., Wennmohs, F., Hansen, A. & Becker, U. Efficient, approximate and parallel Hartree–Fock and hybrid DFT calculations. A 'chain-of-spheres' algorithm for the Hartree–Fock exchange. *Chem. Phys.* **356**, 98–109 (2009).

13. Neese, F. An improvement of the resolution of the identity approximation for the formation of the Coulomb matrix. *J. Comput. Chem.* **24**, 1740–1747 (2003).

14. Te Velde, G. *et al.* Chemistry with ADF. *J. Comput. Chem.* **22**, 931–967 (2001).

15. ADF. (2023).

16. Van Lenthe, E., Baerends, E. J. & Snijders, J. G. Relativistic regular two-component Hamiltonians. *J. Chem. Phys.* **99**, 4597–4610 (1993).

17. Van Lenthe, E., Baerends, E. J. & Snijders, J. G. Relativistic total energy using regular approximations. *J. Chem. Phys.* **101**, 9783–9792 (1994).

18. Van Lenthe, E., Ehlers, A. & Baerends, E.-J. Geometry optimizations in the zero order regular approximation for relativistic effects. *J. Chem. Phys.* **110**, 8943–8953 (1999).

19. Van Lenthe, E. & Baerends, E. J. Optimized Slater-type basis sets for the elements 1–118. *J. Comput. Chem.* **24**, 1142–1156 (2003).

20. Guo, Y., Sivalingam, K. & Neese, F. Approximations of density matrices in N-electron valence state second-order perturbation theory (NEVPT2). I. Revisiting the NEVPT2 construction. *J. Chem. Phys.* **154**, 214111 (2021).

21. Kollmar, C., Sivalingam, K., Guo, Y. & Neese, F. An efficient implementation of the NEVPT2 and CASPT2 methods avoiding higher-order density matrices. *J. Chem. Phys.* **155**, 234104 (2021).

22. Schapiro, I., Sivalingam, K. & Neese, F. Assessment of n-Electron Valence State Perturbation Theory for Vertical Excitation Energies. *J. Chem. Theory Comput.* **9**, 3567–3580 (2013).

23. Garcia-Ratés, M. & Neese, F. Effect of the Solute Cavity on the Solvation Energy and its Derivatives within the Framework of the Gaussian Charge Scheme. *J. Comput. Chem.* **41**, 922–939 (2020).

24. Barone, V. & Cossi, M. Quantum Calculation of Molecular Energies and Energy Gradients in Solution by a Conductor Solvent Model. *J. Phys. Chem. A* **102**, 1995–2001 (1998).

25. Pye, C. C. & Ziegler, T. An implementation of the conductor-like screening model of solvation within the Amsterdam density functional package. *Theor. Chem. Acc. Theory Comput. Model. Theor. Chim. Acta* **101**, 396–408 (1999).

26. Weinhold, F. & Landis, C. R. NATURAL BOND ORBITALS AND EXTENSIONS OF LOCALIZED BONDING CONCEPTS. *Chem Educ Res Pr.* **2**, 91–104 (2001).

27. Glendening, E. D., Landis, C. R. & Weinhold, F. *NBO 6.0* : Natural bond orbital analysis program. *J. Comput. Chem.* **34**, 1429–1437 (2013).

28. Rodríguez, J. I. *et al.* A high performance grid-based algorithm for computing QTAIM properties. *Chem. Phys. Lett.* **472**, 149–152 (2009).

29. Rodríguez, J. I. An efficient method for computing the QTAIM topology of a scalar field: The electron density case. *J. Comput. Chem.* **34**, 681–686 (2013).

30. Lu, T. & Chen, F. Multiwfn: A multifunctional wavefunction analyzer. *J. Comput. Chem.* **33**, 580–592 (2012).

31. Frisch, M. J. *et al.* Gaussian 16, Revision A.03, Gaussian Inc., Wallingford CT. (2016).

32. Aravena, D., Atanasov, M. & Neese, F. Periodic Trends in Lanthanide Compounds through the Eyes of Multireference ab Initio Theory. *Inorg. Chem.* **55**, 4457–4469 (2016).

33. Jung, J., Atanasov, M. & Neese, F. Ab Initio Ligand-Field Theory Analysis and Covalency Trends in Actinide and Lanthanide Free Ions and Octahedral Complexes. *Inorg. Chem.* **56**, 8802–8816 (2017).

34. Bickelhaupt, F. M. & Baerends, E. J. Kohn-Sham Density Functional Theory: Predicting and Understanding Chemistry. in *Reviews in Computational Chemistry* (eds Lipkowitz, K. B. & Boyd, D. B.) vol. 15 1–86 (Wiley, 2000).

35. Albrecht-Schmitt, T. E., Hobart, D. E., Páez-Hernández, D. & Celis-Barros, C. Theoretical examination of covalency in berkelium(IV) carbonate complexes. *Int. J. Quantum Chem.* **120**, e26254 (2020).

36. Walisinghe, A. J. & Chilton, N. F. Assessment of minimal active space CASSCF-SO methods for calculation of atomic Slater–Condon and spin–orbit coupling parameters in d- and f-block ions. *Dalton Trans.* **50**, 14130–14138 (2021).

37. Carnall, W. T. & Fields, P. R. A Study of the Complexes of Curium(III) by Absorption Spectrometry[1]. *J. Am. Chem. Soc.* **81**, 4445–4449 (1959).

38. Cary, S. K. *et al.* A series of dithiocarbamates for americium, curium, and californium. *Dalton Trans.* **47**, 14452–14461 (2018).

39. Vogt, T. B. *et al.* Probing the subtle differences between promethium and curium. *Nat. Commun.* **16**, 3220 (2025).

40. Nielsen, V. R. M. & Sørensen, T. J. Electronic energy levels and optical spectra of trivalent lanthanide ions in water – Ce(III), Pr(III), Nd(III), Sm(III), Eu(III), Gd(III), Tb(III), Dy(III), Ho(III), Er(III), Tm(III), and Yb(III). *Nat. Commun.* **16**, 10754 (2025).

41. Meyer, G. Na_3GdCl_6 : Einkristalle der Tieftemperaturform bei der metallothermischen Reduktion von $GdCl_3$ mit Na. *Z. Für Anorg. Allg. Chem.* **517**, 191–197 (1984).
